# Preferential gene retention increases the robustness of cold regulation in *Brassicaceae* and other plants after polyploidization

**DOI:** 10.1038/s41438-020-0253-0

**Published:** 2020-02-21

**Authors:** Xiao-Ming Song, Jin-Peng Wang, Peng-Chuan Sun, Xiao Ma, Qi-Hang Yang, Jing-Jing Hu, Sang-Rong Sun, Yu-Xian Li, Ji-Gao Yu, Shu-Yan Feng, Qiao-Ying Pei, Tong Yu, Nan-Shan Yang, Yin-Zhe Liu, Xiu-Qing Li, Andrew H. Paterson, Xi-Yin Wang

**Affiliations:** 1 School of Life Science, North China University of Science and Technology, Tangshan, 063210 China; 2 Center for Genomics and Computational Biology, North China University of Science and Technology, Tangshan, 063210 China; 3 Library, North China University of Science and Technology, Tangshan, 063210 China; 4 Fredericton Research and Development Centre, Agriculture and Agri-Food Canada, Fredericton, New Brunswick E3B 4Z7 Canada; 5 Plant Genome Mapping Laboratory, University of Georgia, Athens, GA 30605 USA

**Keywords:** Gene regulation, Abiotic, Plant evolution, Evolutionary biology

## Abstract

Cold stress profoundly affects plant growth and development and is a key factor affecting the geographic distribution and evolution of plants. Plants have evolved adaptive mechanisms to cope with cold stress. Here, through the genomic analysis of Arabidopsis, three *Brassica* species and 17 other representative species, we found that both cold-related genes (*CRGs*) and their collinearity were preferentially retained after polyploidization followed by genome instability, while genome-wide gene sets exhibited a variety of other expansion mechanisms. The cold-related regulatory network was increased in *Brassicaceae* genomes, which were recursively affected by polyploidization. By combining our findings regarding the selective retention of *CRGs* from this ecological genomics study with the available knowledge of cold-induced chromosome doubling, we hypothesize that cold stress may have contributed to the success of polyploid plants through both increasing polyploidization and selectively maintaining *CRGs* during evolution. This hypothesis requires further biological and ecological exploration to obtain solid supporting evidence, which will potentially contribute to understanding the generation of polyploids and to the field of ecological genomics.

## Introduction

A now-classical idea with much circumstantial support is that polyploids adapt better than their diploid progenitors to environmental extremes^1^. At increasingly northerly latitudes, there is an increasing abundance of polyploid plants^2^. The frequency and level of polyploidy peak in arctic regions, where 73% of the flora are polyploids, approximately two-thirds of which have five or more subgenomes^3^. Stebbins reported a correlation between polyploid frequency and glaciation and proposed the hypothesis “second contact” between plant populations that have diverged in different refugia, resulting in hybridization and polyploidy to produce new races, some of which become adapted to different ecological conditions in regions vacated by ice^4,5^.

The success of polyploids is thought to result at least partly from the heterozygosity accompanying polyploidization^6^. It has been proposed that the evolutionary success of allopolyploids in the Arctic may be due to their fixed-heterozygous genomes, which buffer against inbreeding and genetic drift during periods of dramatic climate change^2^. The potential effects of polyploidy on ecological adaptation mediated by altered morphology and physiology remain poorly understood. Since the middle of the last century, many hypotheses have been proposed to explain the abundance and distribution of polyploids^7–9^. Many ecological characteristics have been attributed to polyploids, such as a higher colonization ability, higher tolerance, and greater ecological tolerance or niche breadth, which are associated with invasiveness in novel habitats^10^. However, most of this evidence is anecdotal or comes from observational studies.

Genomics provides valuable opportunities for studying polyploids by allowing deductions to be made about their distant evolutionary past^11^. The sequencing of model plants such as *Arabidopsis* and rice, has revealed recursive polyploidization even though these species often exhibit small genomes^12^. Rich genomic evidence shows that polyploidization could be a major driver of plant evolution correlated with the origination and rapid diversification of major plant taxa^13^. Based on phylogenetic and evolutionary analysis, genomic evidence has been suggested to link bursts of polyploidy to ecological catastrophes such as the Cretaceous-Tertiary (K-T) mass extinction^14^. The K-T extinction, which is generally thought to have been triggered by a massive comet or asteroid impact 66 Mya, resulted in catastrophic effects on global ecology, killing 75% or more species^15^. This event is thought to have caused lingering effects for years, with the persistence of environmental conditions under which it was impossible for plants to carry out photosynthesis. However, the link between mass extinction and polyploidization is weakened by uncertainty in the estimation of the ages of polyploidization events^16^. Moreover, polyploidization events were by no means scarce either before or after the K-T extinction, with some such event being quite recent^17^. Nevertheless, these facts do not eliminate the possibility of a relationship between polyploidization events and ecological changes.

Temperature profoundly influences the metabolism of plants and is thus a key factor determining their geographic distribution and growing season^18,19^. Cold stress, including chilling and/or freezing, adversely affects plant growth, development, and crop productivity^20^. Plants have evolved a repertoire of adaptive mechanisms for coping with cold temperature, including seed and bud dormancy, vernalization, photoperiod sensitivity, supercooling, and especially cold acclimation^19,21^. Most plants can undergo cold acclimatization and acquire tolerance to extracellular ice formation in their vegetative tissues^22^. The exploration of the freezing tolerance of these species has contributed greatly to the understanding of cold stress^21,23^.

All species are the products of ecological systems and are continuously affected by ecological changes. Considering that selection may leave genomic signatures, we investigated whether there is nonrandom enrichment of *CRGs* after polyploidization in the present study. Cold stress induces the expression of transcription factors (TFs) such as C-repeat binding factors (*CBFs*), which can activate the expression of downstream cold-responsive (*COR*) genes^24^. *CBF* genes are regulated by several upstream TFs, including the basic helix-loop-helix (*bHLH*) and inducer of *CBF* expression 1 (*ICE1*) proteins, among others^20^.

In this study, we conducted comprehensive comparative analyses of *CRGs* to explore their retention, positive selection, expression and regulatory networks after polyploidy. By studying tens of sequenced plant genomes affected by multiple polyploidization events, we sought to minimize bias due to lineage-specific characteristics, elucidating general principles of the relationship between polyploidy and ecological adaptation.

## Results

### Recursive polyploidization and accumulation of *CRG* analogs in *Arabidopsis*

First, in the angiosperm model *Arabidopsis thaliana*, we systematically collected 115 *CRGs* based on previous reports (Table S1, Arabidopsis *CRGs* list). Then, using these 115 genes, we identified 420 *CRG* analogs via BlastP searches (*E*-value <1 × 10^−10^, amino acid identity >60%: Table S2). To determine whether the 420 *CRG* analogs could be related to ancestral polyploidization events, a program from the MCScanX packages that could detect different types of gene duplication was implemented as reported^25^. All *CRGs* were classified into five groups: singletons (5, 1.19%), dispersed genes (42, 10.00%), proximal duplicates (3, 0.71%), tandem duplicates (17, 4.05%), and polyploidy-related genes (353, 84.05%) (Figure S1a, Table S3-4). Colinear genes between homoeologous regions are those whose ancestral locations are preserved, which are often used to infer paleo-polyploidy events^6^. Here, polyploidy-related genes were inferred based on collinearity persisting in the regions surrounding the selected genes, albeit noting that the result may be an underestimate due to the erosion of gene collinearity and gene losses after polyploidization^26^. Compared with the total of 27,417 genes annotated in *Arabidopsis* classified into the above five types, *CRGs* were extremely overrepresented (Chisq-test *P*-value = 1.92E−151) in polyploidy-related groups and underrepresented in all other groups (Table S4). In addition, we used the Gene Ontology (GO) database to identify *CRGs*, and the results were similar to those of the above analysis (Table S5).

### Recursive polyploidization contributed to the expansion of rice *CRGs*

Using the sequences from the CRG library of *Arabidopsis* and rice, we identified 430 homologs by BLAST searches in rice, a model monocot plant (Table S2). The classification of rice cold-related homologs into singletons (5), dispersed genes (101), proximal or tandem duplicates (23), and polyploidy-related genes (301) showed significant overrepresentation in the polyploidy-related group (*P*-value ~ 0) (Figure S1, Table S3-4) and underrepresentation in other groups (Table S4). Specifically, ~70% (301 of 430) of cold-related colinear genes were associated with recursive polyploidization according to our previous classification^27^ (Table 1, Table S6). These findings suggested that polyploidization might be correlated with the accumulation of cold-related homologs in rice and possibly other grasses.

**Table 1:** Collinearity analysis of *CRGs* in 21 plants

Species	All genes			CRGs				
	Colinear gene	Total genes	Percent (%)	Colinear gene	Total genes	Percent (%)	*P*-value	Status
*M. truncatula*	3657	44,135	8.29	52	291	17.87	2.68E−09	I
*G. max*	36,986	56,044	65.99	704	813	86.59	8.59E−36	I
*M. domestica*	9417	30,294	31.09	176	380	46.32	1.08E−10	I
*A. thaliana*	7519	27,417	27.42	353	420	84.05	1.92E−151	I
*B. napus*	61,229	101,040	60.6	1290	1520	84.87	8.77E−85	I
*B. oleracea*	24,148	59,225	40.77	793	852	93.08	5.96E−215	I
*B. rapa*	23,489	41,019	57.26	720	750	96.00	7.07E−104	I
*G. hirsutum*	51,298	70,478	72.79	1093	1210	90.33	1.64E−43	I
*G. arboreum*	8353	40,134	20.81	449	600	74.83	1.54E−236	I
*G. raimondii*	16,150	37,505	43.06	562	618	90.94	8.96E−130	I
*P. trichocarpa*	22,059	41,335	53.37	490	502	97.61	6.40E−89	I
*V. vinifera*	3615	26,346	13.72	190	316	60.13	1.53E−128	I
*S. lycopersicum*	6580	34,725	18.95	359	400	89.75	3.45E−289	I
*Z. mays*	11,711	38,914	30.09	510	560	91.07	2.21E−220	I
*O. sativa*	5728	55,801	10.27	301	430	70.00	0.00E+00	I
*A. comosus*	4987	27,024	18.45	235	285	82.46	1.58E−172	I
*M. acuminata*	13,282	36,550	36.34	414	652	63.50	6.02E−48	I
*E. guineensis*	9321	30,752	30.31	229	292	78.42	3.08E−72	I
*A. trichopoda*	745	26,846	2.78	21	199	10.55	2.02E−11	I
*S. moellendorffii*	4263	22,285	19.13	26	124	20.97	6.02E−01	U
*P. patens*	3980	30,249	13.16	67	209	32.06	5.01E−16	I

The abundance of colinear genes was compared between CRGs and all genes via chi-squared tests, indicating a significant increase (I), no change (U), or a decrease (D), respectively

### Polyploidization contributed to the expansion of *CRGs* in other species

**Fig. 1:**
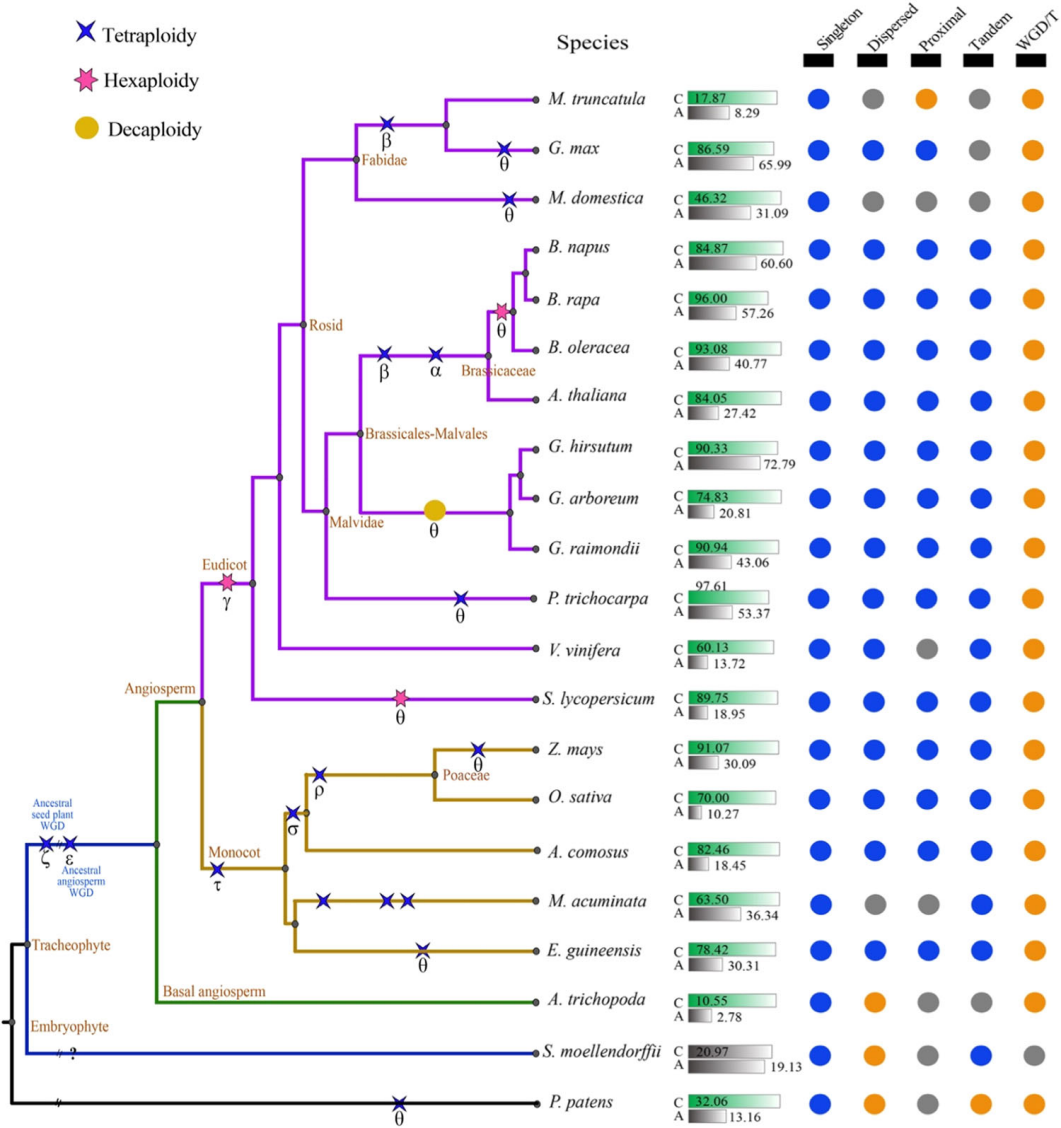
Plant phylogeny, paleopolyploidization, and enrichment of cold-related genes. Information regarding polyploidization was obtained from the Plant Genome Duplication Database (PGDD). The percentages of cold-related genes and all genes in colinear regions are displayed in parallel, gradient rectangles, where C: cold-related genes (*CRGs*), and A: all genes in each genome; each gene classification is displayed with solid circles, with significance levels relative to genome-wide averages shown in different colors: enriched (orange, *P* < 0.01), depleted (blue, *P* < 0.01), or not significantly different (gray)

In addition to *Arabidopsis* and rice, we investigated the relationship between the accumulation of *CRGs* and polyploidization in a total of 21 species, including model plants such as *Zea mays*, a basal angiosperm (*Amborella trichopoda*), a lycophyte (*Selaginella moellendorffii*), a moss (*Physcomitrella patens*) and other land plants (Fig. 1, Table S2, Table S7). There were more cold-related homologs in *Brassica napus* (1520) than in any other plant, followed by *Gossypium hirsutum* (1210) and *Brassica oleracea* (852). In contrast, *S. moellendorffii* exhibited the fewest *CRG* homologs (124) (Table S6, Fig. 1). At the whole-genome level, 1.83% (750) of *Brassica rapa* genes belonged to the cold-related group, followed by the proportions in *Musa acuminata* (1.78%, 652) and *G. hirsutum* (1.72%), while only 0.56% of *S. moellendorffii* genes belonged to this group (Table S3).

In 20 of the 21 species, with the exception of *S. moellendorffii*, cold-related homologs were significantly enriched (*P* < 0.05) for polyploidy-related genes at the whole-genome level (Table 1, Table S6). In 17 of the 21 plant species, polyploidization was the predominant mechanism by which *CRG* homologs formed, accounting for 46.32–96.00% of these homologs in each genome (Figure S1, Table S6). *CRGs* were also enriched for dispersed duplications in three plants (*A. trichopoda*, *P. patens*, and *S. moellendorffii*) and proximal duplications in one plant (*M. truncatula*). *CRGs* were underrepresented (*P* < 0.05) among singleton genes in all 21 species (Fig. 1, Table S4). Notably, nucleotide-binding site (*NBS*) genes showed a trend that was precisely opposite that of *CRGs*. The most NBS genes were found in *M. truncatula* (565, 1.28%), followed by *P. trichocarpa* (563, 1.36%) and *O. sativa* (500, 0.90%), while *NBS* genes represented <0.50% of the gene sets of *B. napus, G. hirsutum*, *B. rapa*, and *M. acuminata* (Table S8). These phenomena indicated that the expansion and evolutionary mechanisms were different between *CRGs* and *NBS* genes. Whole-genome duplication/triplication (WGD/T) and segmental duplication played a much greater role in the expansion of *CRGs* than *NBS* genes. Overall, these findings suggested that the expansion of *CRGs* in a wide range of plants might be correlated with polyploidization.

### Hierarchical analyses of recursive polyploidies and *CRG* expansion

**Fig. 2:**
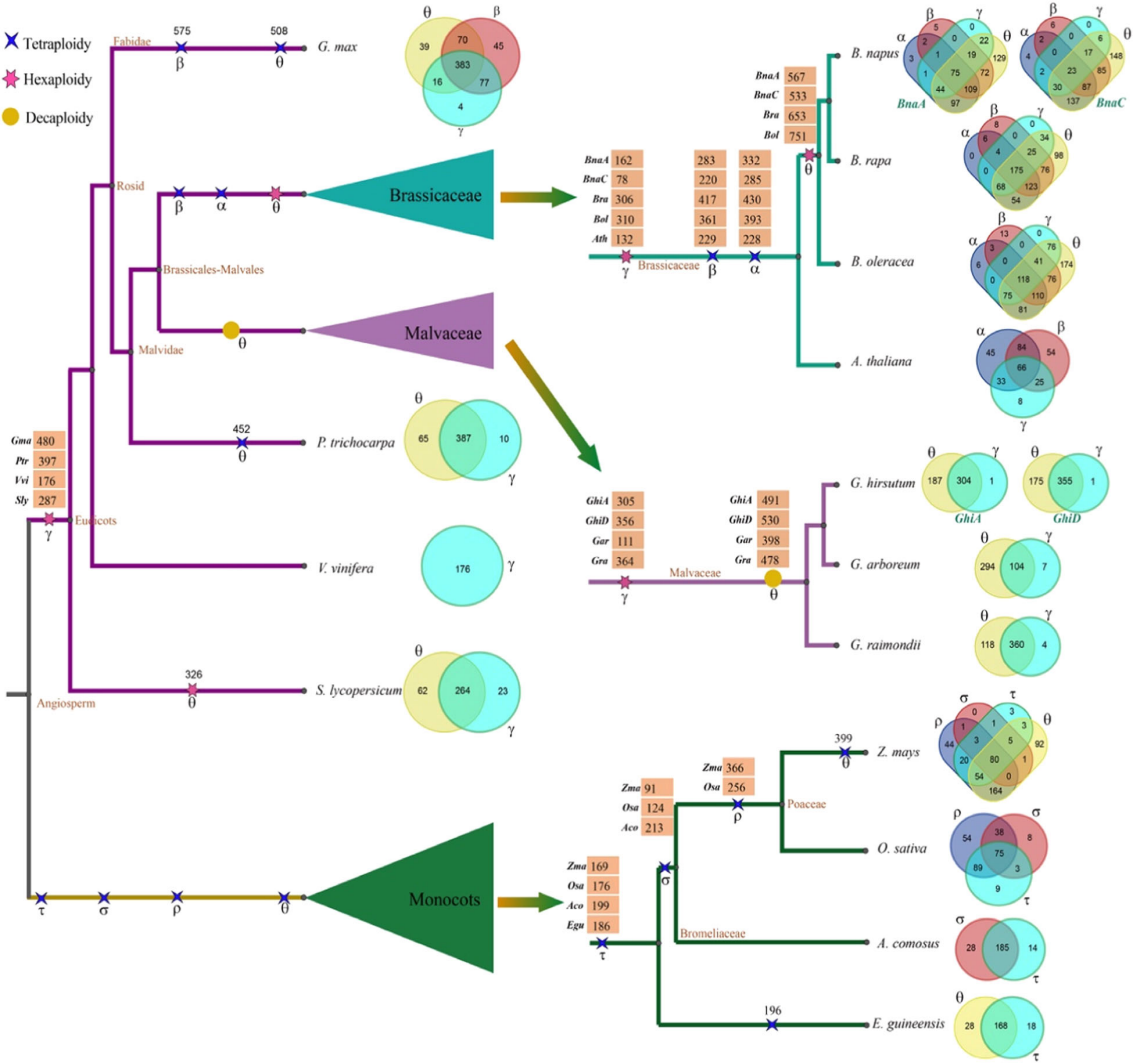
CRG expansion as a result of polyploidization events during the evolution of different plant groups. A blue four-pointed star represents tetraploidy events, including β, α, τ, σ, ρ and some θ events. A red six-pointed star represents hexaploidy events, including γ and some θ events. A yellow circle represents decaploidy events, which occurred only in Malvaceae. The number on the branch represents the number of *CRGs* included in each species related to each duplication event. Venn diagrams show the numbers of common and unique *CRGs* in each examined species

The 21 plants in which we investigated the accumulation of *CRGs* have experienced several independent polyploidizations (Fig. 2). Using a hierarchical and event-related approach by which we deciphered gene collinearity in many other genomes^28,29^, we identified colinear genes produced by each of the WGD/T events. *CRGs* were significantly increased in colinear regions in all species except for *S. moellendorffii*. The most recent events generally contributed the most to the enrichment of *CRGs* (Fig. 2, Table 2).

**Table 2:** *CRG* expansion analysis for each polyploidization event in selected species

Species	Event 1			Event 2			Event 3			Event 4		
	Num#	Per (%)	Events	Num#	Per (%)	Events	Num #	Per (%)	Events	Num #	Per (%)	Events
*G. max*	480	59.04	WGT (γ)	575	70.73	WGD (β)	508	62.48	WGD (θ)	NA	NA	NA
*A. thaliana*	132	31.43	WGT (γ)	229	54.52	WGD (β)	228	54.29	WGD (α)	NA	NA	NA
*B. napus A*	162	21.60	WGT (γ)	283	37.73	WGD (β)	332	44.27	WGD (α)	567	75.60	WGT (θ)
*B. napus C*	78	10.13	WGT (γ)	220	28.57	WGD (β)	285	37.01	WGD (α)	533	69.22	WGT (θ)
*B. oleracea*	310	36.38	WGT (γ)	361	42.37	WGD (β)	393	46.13	WGD (α)	751	88.15	WGT (θ)
*B. rapa*	306	40.80	WGT (γ)	417	55.60	WGD (β)	430	57.33	WGD (α)	653	87.07	WGT (θ)
*G. hirsutum A*	305	51.35	WGT (γ)	491	82.66	Decaploid (θ)	NA	NA	NA	NA	NA	NA
*G. hirsutum D*	356	57.79	WGT (γ)	530	86.04	Decaploid (θ)	NA	NA	NA	NA	NA	NA
*G. arboreum*	111	18.50	WGT (γ)	398	66.33	Decaploid (θ)	NA	NA	NA	NA	NA	NA
*G. raimondii*	364	58.90	WGT (γ)	478	77.35	Decaploid (θ)	NA	NA	NA	NA	NA	NA
*P. trichocarpa*	397	79.08	WGT (γ)	452	90.04	WGD (θ)	NA	NA	NA	NA	NA	NA
*V. vinifera*	176	55.70	WGT (γ)	NA	NA	NA	NA	NA	NA	NA	NA	NA
*S. lycopersicum*	287	71.75	WGT (γ)	326	81.50	WGT (θ)	NA	NA	NA	NA	NA	NA
*Z. mays*	169	30.18	WGD (τ)	91	16.25	WGD (σ)	366	65.36	WGD (ρ)	399	71.25	WGD (θ)
*O. sativa*	176	40.93	WGD (τ)	124	28.84	WGD (σ)	256	59.53	WGD (ρ)	NA	NA	NA
*A. comosus*	199	69.82	WGD (τ)	213	74.74	WGD (σ)	NA	NA	NA	NA	NA	NA
*E. guineensis*	186	63.70	WGD (τ)	196	67.12	WGD (θ)	NA	NA	NA	NA	NA	NA

WGD/WGT represents whole-genome duplication/triplication. The WGD or tetraploidy events include β, α, τ, σ, ρ and some θ events. The WGT or hexaploidy events include γ and some θ events. Decaploidy indicates the θ event of *Malvaceae* species. “NA” indicates species that did not experience the corresponding genome duplication events. Collinearity analysis of *CRGs* was conducted using the MCScanX program

For example, *Arabidopsis* has been affected by one polyploidization common to core eudicots and two shared with other *Brassicaceae* plants^30,31^. By checking the gene collinearity inferred above and polyploidization-produced paralogous regions reported previously^32^, 31.43, 54.52, and 54.29% of the 420 cold-related homologs could be connected to the respective events in temporal order. Genes associated with an older event were recounted if they were reduplicated in a younger event. This general pattern was found for nearly all events affecting the other plants under study (Table 2), consistent with the fact that gene collinearity is better preserved from recent than ancient events.

In contrast, colinear regions were poor in *NBS* genes in nearly all species (Table S6). The ratio of *NBS* genes in colinear regions was significantly lower than that at the whole-genome level in almost all species, except for *M. truncatula*, *A. trichopoda*, and *S. moellendorffii* (Table S9). *NBS* genes largely expanded by proximal and tandem duplications through a rapid birth-and-death process.

### Regulatory network analyses

#### Networks constructed from all candidate CRGs

By using the *Arabidopsis* Interactions Viewer with data obtained from several methods, such as gene coexpression analysis (see Methods), we constructed an interaction network for all 420 inferred *CRGs* of *Arabidopsis*. The software queries a database of 70,944 predicted and 39,505 confirmed interacting proteins in *Arabidopsis*. For other species, we also characterized protein–protein interactions among putative orthologs of the *Arabidopsis CRGs* using the established *Arabidopsis* interaction network and predictions from the method of equivalent replacement. The inferred interaction relationships were illustrated using Gephi software^33^.

**Fig. 3:**
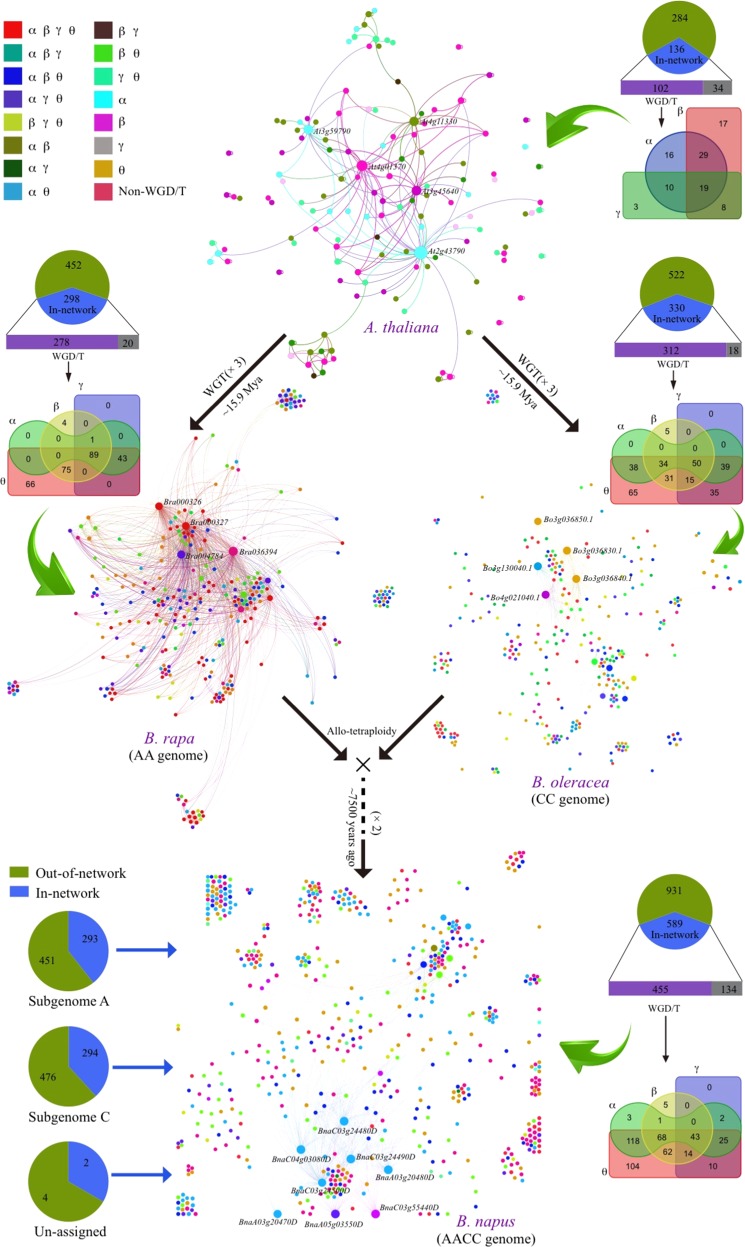
Interaction networks for the identified CRGs in Arabidopsis thaliana, B. rapa, B. oleracea, and B. napus. The network of *CRGs* in *Arabidopsis* was constructed using *Arabidopsis* Interactions Viewer (v2.0). The networks associated with the *Arabidopsis* orthologs of *CRGs* in the other three species were constructed using Gephi (v0.8.2) software. The pie charts show the number of *CRGs* located in the network and outside of the network. WGD (tetraploidy) events include β, α, and θ events, while γ represents a WGT (hexaploidy) event. Venn diagrams show the numbers of *CRGs* common or specific to different polyploidization events (α, β, γ, and θ). Dots represent α (blue), β (purple), γ (green), and θ (orange) events, respectively

We found that taxa that have experienced more duplication events generally exhibited much larger interaction networks. For example, *Brassica* plants (*B. rapa*, *B. oleracea*) have undergone triplication since divergence from *Arabidopsis*^34–36^, and *B. napus* (AACC genome) originated via hybridization between *B. rapa* (AA genome) and *B. oleracea* (CC genome)^31^. The interaction network of *CRGs* was much larger in *B. rapa* and *B. oleracea* than in *Arabidopsis* and was larger still in *B. napus*. For *Arabidopsis*, 136 of 420 *CRGs* were involved in the network, among which 102 could be related to polyploidy events, with 3, 17, and 16 related to γ, β, and α polyploidizations, respectively (Fig. 3, Table S10). For *B. rapa*, 39.7% (298 of 750) of *CRGs* were involved in the network, and 278 were related to polyploidy events, with 0, 4, 0, and 66 genes that were specific to γ, β, α, and θ (the latest) events, respectively (Fig. 3, Table S10). For *B. oleracea*, 330 of 852 *CRGs* were involved in the network, among which 312 were related to polyploidy, with 0, 5, 0, and 65 being specific tο γ, β, α, and θ events, respectively. For *B. napus* (Fig. 3, Table S10), 589 of 1520 *CRGs* were involved in the network, with 293 and 294 coming from subgenomes A and C, respectively. Among these genes, 455 genes were related to polyploidy, with 0, 5, 3, and 104 that were specific to γ, β, α, and θ events.

Genes from plants that have experienced more polyploidization exhibited more interactions. Among these four networks, the maximum edge number for a single *Arabidopsis* gene was only 39 (*At2g43790*), vs. 356, 262, and 676 for the *B. rapa*, *B. oleracea*, and *B. napus* genes, respectively (Figure S2a, Table S11). Likewise, the total edge number was highest for *B. napus* (7887), followed by *B. oleracea* (2722), *B. rapa* (2165), and *Arabidopsis* (213) (Figure S2b).

The complexity of the regulatory networks was likely related to transcription factors (TFs). More than half of the *CRGs* were putative TFs in *Arabidopsis* and *Brassica* (Figure S3a, Tables S12, 13). A total of 16 types of TFs were identified among all *CRGs*, including *ethylene-responsive factor* (*ERF*), *myeloblastosis* (*MYB*), *APETALA2* (*AP2*), *related to ABI3/VP1* (*RAV*), *cysteine 2/histidine 2* (*C2H2*), *NAM*, *ATAF1*/2, and *CUC2* (*NAC*) TFs, with *ERF* and *MYB* being significantly more abundant than other types of TFs (Figure S3b, Table S12), which indicated that these two kinds of TFs might play important roles in cold regulation networks in the studied plants.

#### Networks constructed with reported CRGs

**Fig. 4:**
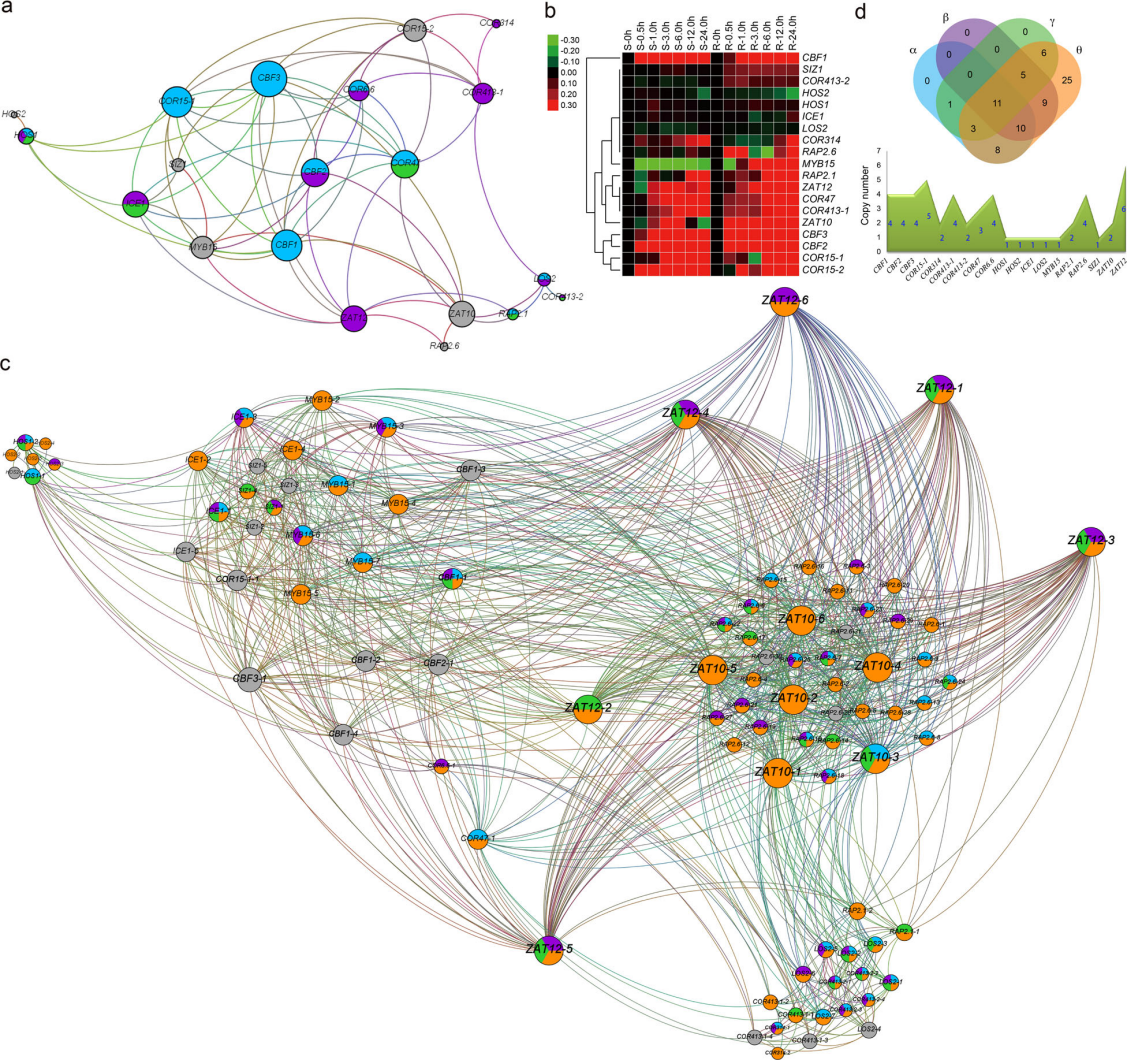
Analyses of key CRGs in Arabidopsis and B. napus. **a** An interaction network of key *CRGs* in *Arabidopsis* was constructed based on *Arabidopsis* Interactions Viewer. Dots represent α (blue), β (purple), γ (green), and θ (orange) events. **b** Expression cluster analyses were performed for key *CRGs* under different durations (0, 0.5, 1.0, 3.0, 6.0, 12.0 and 24.0 h) of cold treatment of the shoots (S) and roots (R) in *Arabidopsis*. The expression values were log2 transformed for heatmap cluster analyses. Red and green colors represent up- and downregulated genes, respectively. **c** The networks associated with the *Arabidopsis* orthologs of key *CRGs* in *B. napus* were constructed using Gephi software. Dots represent α (blue), β (purple), γ (green), and θ (orange) events, respectively. **d** Copy numbers of key *CRGs* from *Arabidopsis* in *B. napus*. Venn diagrams indicate the numbers of *CRGs* that are common or specific to different events in *B. napus*

We investigated previously reported cold regulation networks in *Arabidopsis*^19,20^ and selected 20 key *CRGs* for further analyses. The interaction networks among putative orthologs of the *Arabidopsis CRGs* in other plants were inferred using the above methods (Table S14) (Fig. 4a). The published data on the expression of these *CRGs*^37^ came from two tissues, shoots and roots, under different durations of cold treatment (0, 0.5, 1.0, 3.0, 6.0, 12.0, and 24.0 h) (Table S15). Most of the examined *CRGs* were upregulated after cold treatment, with only *high expression of osmotically responsive genes 2* (*HOS2*) being downregulated after 24 h of treatment (Fig. 4b). For *MYB15*, expression was downregulated in some time periods but upregulated after cold treatment for 1–24 h. These expression changes indicated that these genes play important roles in the cold response of *Arabidopsis*. Furthermore, we studied the expression of these genes in seven different tissues, including the roots, stems, leaves, shoots, flowers, pollen, and seeds. Some genes showed tissue-specific expression; for example, *MYB15* exhibited much higher expression levels in the roots than in other tissues (Figure S4, Table S16).

Among these networks, the maximum edge number for a single gene (*zinc finger of Arabidopsis thaliana 10*, *ZAT10*) was 60 in *B. napus* (Table S17). The total edge number was highest for *B. napus* (1022), followed by *B. oleracea* (418) and *B. rapa* (300) (Figure S2c d, Table S18). The cold regulation networks of several species, such as *B. napus*, *B. oleracea*, *B. rapa*, and *M. acuminata*, were much larger than those of *Arabidopsis* (Fig. 4a, c, Figure S5). The ratios of edge number to node number in these four species were higher than in *Arabidopsis* (3.35) and other examined species. The highest ratio was detected in *B. napus* (10.76), followed by *B. oleracea* (7.46) and *B. rapa* (5.45) (Table S18). Among these key *CRGs*, one or more copies were identified in each of *B. napus*, *B. oleracea*, and *B. rapa*, except for *SIZ1* in *B. rapa* (Fig. 4d).

**Fig. 5:**
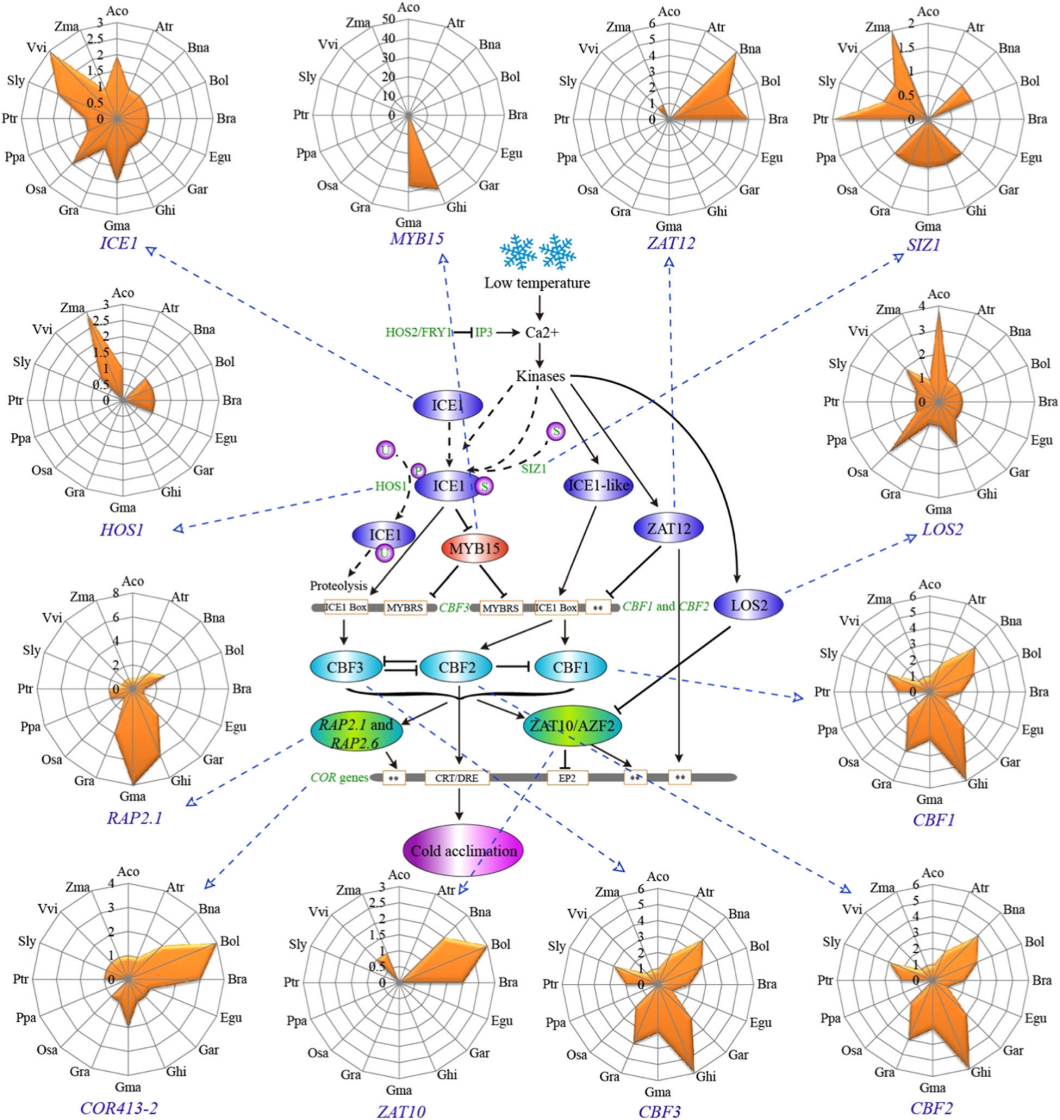
Number of genes in each examined plant orthologous to key Arabidopsis CRGs. The regulatory network of key *CRGs* was drawn, mainly in reference to a previous report^20^. The key orthologous *CRGs* in *B. napus* and other examined species were identified using the OrthoFinder program

Similarly, we identified more copies of the *CBF1-3*, *MYB15*, *related to AP2 1* (*RAP2.1*), and *ICE1* genes in most species than were found in *Arabidopsis* (Fig. 5). There were 42 and 37 *MYB15* copies in *G. hirsutum* and *G. max*, respectively (Table S14, Fig. 5). However, we failed to detect an ortholog of *Arabidopsis COR15-2* in all species. For *COR15-1*, *COR47*, and *COR6.6*, orthologous genes were only detected in *B. napus*, *B. rapa*, and *B. oleracea*, while none were found in all other species (Figure S6, Table S14). In *P. patens*, we detect no copies of 14 of these genes, one copy of 4, and two copies only for *RAP2.1*, which could reflect sequence variation, high structural variation or deletions.

There were no *B. napus CRGs* specific to γ, β, or α polyploidization events, while 25 were specific to the most recent event (θ) (Fig. 4d). In addition, we detected 11 genes that were duplicated in all four genome duplication events (γ, β, α, and θ). For *B. oleracea*, 0, 2, 0, and 15 key *CRGs* were specific to γ, β, α, and θ events, respectively (Figure S5), with 5 genes involved in all four types of genome duplication events. For *B. rapa*, no *CRG* was specific to γ, β, or α events, but 9 were specific to the most recent polyploidization (θ) (Figure S5), and 14 were duplicated in all four types of genome duplication events.

#### Strengthened CRG regulatory networks after polyploidization

The robustness of *CRG* regulatory networks was enriched with extra homologs after polyploidization. We evaluate the robustness value (*R*) of a network, reflecting the size of the largest linked subgraph after randomly removing certain numbers of nodes from an initial network or graph (see Methods for details). We used two approaches to compare the robustness values of *Arabidopsis* and *Brassica* plants. First, the same number of nodes was removed from each network, comparing the corresponding networks (Table 3). For the networks constructed from the key *CRGs*, when 5 genes were removed from each network, the *Arabidopsis* network presented a robustness value of 0.967, which was lower than those for the *Brassica* plants. The *Arabidopsis* network robustness was still lower even after the removal of 10 nodes of *Brassica* plants (≥0.998). Second, the same percentages of nodes were removed. When 5% of nodes were removed in the network constructed with key *CRGs*, the *Arabidopsis* network presented a robustness value of 0.992, compared with 0.995, 1.000, and 1.000 for *B. rapa, B. oleracea*, and *B. napus*, respectively, and its robustness value remained lower even after 20% of nodes were removed from the *B. oleracea* and *B. napus* networks. Robustness was also checked with the networks constructed from all candidate *CRGs*, which resulted in the same conclusion (Table 3).

**Table 3:** The assessment of interaction networks constructed using reported *CRGs* and all candidate *CRGs* in *Arabidopsis*, *B. rapa*, *B. oleracea*, and *B. napus*

	All nodes in a network	5	10	5%	10%	20%	50%
	Reported *CRGs*						
*A. thaliana*	20	0.967	0.881	0.992	0.991	0.976	0.882
*B. rapa*	55	0.998	0.988	0.995	0.994	0.983	0.945
*B. oleracea*	56	0.999	0.997	1.000	1.000	0.997	0.976
*B. napus*	95	1.000	0.999	1.000	0.999	0.997	0.981
	All candidate *CRGs*						
*A. thaliana*	113	0.962	0.918	0.959	0.906	0.820	0.519
*B. rapa*	288	0.999	0.995	0.995	0.990	0.974	0.885
*B. oleracea*	317	0.995	0.983	0.995	0.976	0.964	0.877
*B. napus*	565	1.000	1.000	0.999	0.997	0.988	0.949

Network robustness was calculated after the removal of certain numbers (5 or 10) or percentages (5%, 10%, 20%, or 50%) of nodes

### Positive selection analyses of key *CRG* families

To explore the selective pressures acting on genes involved in the cold regulation network, we collected and selected key *CRGs* according to previous reports^19,20^. The best BlastP matches or likely orthologs from all plants were used to perform positive selection analyses (Figure S7). A total of 118 branch nodes showing positive selection were detected in 16 phylogenetic trees (Table S19). The greatest number of branch nodes with positive selection (16) was detected in the *COR15* gene family, followed by *CBF* (12) and *COR47* (10) (Figure S8). In contrast, there were few nodes showing positive selective in the *COR6.6* (3), *LOS2* (4), *MYB15* (4), and salt-induced *zinc finger protein 1* (*SIZ1*) (4) gene families (Table S19).

### Dosage effect analysis

We identified *B. rapa* genes that were colinear with key *Arabidopsis CRGs*. The results showed that 1, 5, 8, and 5 key *Arabidopsis CRGs* exhibited 0–3 colinear orthologs in *B. rapa* (Figure S9a, Table S20). The expression patterns of five groups with three colinear orthologs were studied in six tissues of *B. rapa*. We found that several colinear genes exhibited similar expression trends among tissues (Figure S9b, c), although the expression levels were slightly different. These findings indicated that these colinear homologs may play similar or related roles after duplication.

We found that in several cases, colinear orthologs presented divergent expression patterns, showing likely dosage differentiation or compensation effects (Figure S9d–f). For example, *Bra002527* exhibited much higher expression in almost every tissue than its two colinear homologs. A similar phenomenon was found for *Bra023742*, which presented significantly higher expression in roots than the other two colinear homologs. These findings might also indicate that polyploidy-produced homologs may have diverged in function, possibly resulting in subfunctionalization or neofunctionalization. In addition, we found that some genes exhibited tissue specificity in their expression. *Bra020284* and *Bra023742* were highly expressed in roots but not in other tissues.

## Discussion

Numerous lines of evidence suggest that polyploidization is associated with the expansion of gene families related to the cold temperature response, an association that may be related to the direct effects of cold on gamete formation^38^. Cold stress can alter the formation of radial microtubule arrays at telophase II and lead to the formation of diploid and polyploid pollen grains, which will ultimately increase the possibility of polyploidization^39^. Genes including those encoding transcription factors are involved in determining the sensitivity of male gametogenesis to cold stress^40^. Thus, cold temperatures may lead to the formation of polyploidy, and cold tolerance may be important for the survival of newly formed polyploids. High temperature can also induce the production of unreduced pollen in *Populus*^41^ and in *Rosa* sp.^42^. It is likely that temperature stresses disrupt the normal process of cell division and can cause chromosome number increases in plants. Previous analyses demonstrated that extreme cold temperatures may represent an absolute boundary for species survival, whereas warm temperatures do not^43^. The observation of more polyploids in higher-latitude regions in the Northern Hemisphere agrees with this inference^2^. Here, the analysis of the abundance and expansion of *CRGs* in dozens of land plants provided further support for this hypothesis.

Experimental evidence suggests that both *CRGs* and heat tolerance genes are often members of the temperature stress-responsive or *thermostress*-*responsive* (*TSR*) gene families. For example, the transgenic overexpression of a *Primula forrestii* heat-induced gene, *PfHSP17.2*, in *Arabidopsis* increased cold tolerance^44^. The overexpression of a zinc finger transcription factor (*TaZnF*) from wheat conferred tolerance to both heat and cold stresses in *Arabidopsis*^45^. Even in insects, most heat shock protein (HSP) genes are induced or repressed by thermal stress (40 °C) and cold treatment (4 °C)^46^. Strong evidence led to the conclusion that certain temperature stress-responsive gene families, or even individual members of these families, may be involved in the responses to both high- and low-temperature stresses. Therefore, it was no surprise that we identified the preferential retention of cold-related family genes as an outcome of paleopolyploidization in plants from both cold region and warm regions. We conducted analyses of heat shock transcription factors (*Hsfs*) among 21 representative plants (Table S21). The repeat types were identified for these *Hsf* genes and compared with those of cold-related genes in each species. Six species showed more and three species showed fewer WGD-type duplications than non-WGD-type duplications (Table S21). On average, 50.80% of *Hsf* genes belonged to WGD events (including segmental duplications), confirming that some heat-related genes were also enriched by WGD, even though the number was smaller than that of cold-related genes. We are not aware of any reports that heat induces polyploidy.

Plants with doubled genomes have been suggested to present a greater chance of survival in dramatically altered environments^14^. Polyploidization was previously related to the occurrence of mass extinctions, albeit with some problems^16^. Catastrophic events such as volcanic eruptions and widespread global cooling (ice ages) could have triggered mass extinctions^47^. However, we must note that icy or cold days may occur nearly every year and in nearly all regions on Earth, thereby affecting plant cell division, and a single undivided cell may ultimately grow into a polyploid plant^15^. Previous research has shown that angiosperms had to achieve new structural and functional trait solutions before making forays into cold environments^48^. Our findings suggest that the expansion of cold-related genes in a wide range of plants might be correlated with polyploidization. *CRGs* were significantly more often retained in polyploids compared with the genome-wide average. Moreover, *CRGs* were enriched in genomic regions produced by recent WGD/T events, suggesting that recurrent polyploidization may enrich the repertoire of *CRGs*. One exception, *S. moellendorffii*, was inferred to present the fewest *CRG* homologs, possibly due to its fragmented genome assembly^49^.

Moreover, previous studies have indicated that polyploid plants exhibit tolerance to a wider range of environmental conditions than their diploid relatives^50^. This is thought to occur because gene duplication promotes diversification and evolutionary success through the emergence of novel gene functions^51^. The overrepresentation of *CRGs* in the polyploidy-related gene group suggests that polyploidization has contributed significantly more to the expansion of *CRGs* than the other four gene duplication types. Moreover, if all genes were equally affected by collinearity erosion, newer polyploidization events should have made the largest contributions not only to the *CRG* reservoir but also to other gene families, as those associated with older events decrease over time. This is clearly not a general characteristic of all large gene families; for example, *NBS* genes are predominantly clustered, and ectopic duplication is the major cause of their expansion^52^. Although *CRGs*, among which the *NBS* genes are a major component, have expanded via various mechanisms other than WGD^53^, the present study confirmed this phenomenon in a diverse group of taxa and provided a direct opposite example to the group of *CRGs* (Tables S8, 9, 22). In addition, we identified drought-related genes in *Arabidopsis* that were not significantly enriched during polyploidization.

Many WGD/T events are distributed across angiosperms and might lead to gene expression changes and epigenetic remodeling^47^, which may provide variation that allows rapid adaptation to novel environments^54^. Altered reproductive modes, increased phenotypic variability, and subfunctionalization may allow polyploids to survive in conditions that are beyond the adaptive range of their diploid progenitors^55^. For example, arctic allopolyploids have been particularly efficient in invading deglaciated (relatively warmer) areas during periods of dramatic climate change^2^.

The plasticity of expression patterns might confer polyploids with a wider phenotypic range than their diploid progenitors. Based on various environmental stresses, duplicated genes could become subfunctionalized by partitioning ancestral expression patterns, which might improve adaptation to different ecological conditions^56^. In addition, several key *CRGs*, such as *COR15* and *CBF* genes, show evidence of positive selection in their evolutionary history. Most *CRGs* belong to transcription factor families, and their regulatory networks are much larger in plant genomes affected by more polyploidization events. These results suggest that polyploidy plays an important role in resisting the stress imposed by a cold external environment. These findings may contribute to understanding the formation of polyploids and contribute new knowledge to the field of ecological genomics.

## Materials and methods

### Retrieval of sequences

The *B. napus, B. oleracea*, and *B. rapa* genome sequences were retrieved from the BRAD database^57^. The *Arabidopsis* and rice genome sequences were downloaded from the TAIR (https://www.arabidopsis.org) and RGAP (http://rice.plantbiology.msu.edu) databases, respectively. The *A. trichopoda* genome sequences were obtained from the *Amborella* Genome Database^58^. The *S. lycopersicum* genome sequences were downloaded from the Sol Genomics Network (https://solgenomics.net). The *G. hirsutum*, *G. arboretum*, and *G. raimondii* genome sequences were downloaded from CottonFGD (http://www.cottonfgd.org). The *M. acuminata* and *M. domestica* genome sequences were downloaded from the Banana Genome Hub (https://banana-genome-hub.southgreen.fr) and GDR (https://www.rosaceae.org), respectively. The genome sequences of the other nine examined species (*M. truncatula, G. max, P. trichocarpa, V. vinifera, Z. mays, A. comosus, E. guineensis, S. moellendorffii*, and *P. patens*) were downloaded from JGI Phytozome 12 (https://phytozome.jgi.doe.gov/pz/portal.html). These species are representative of different branches in the plant phylogenetic tree, and the WGD/WGT information was obtained through searches in the plant genome duplication database^59^.

### Identification and characterization of *CRGs* and *NBS* genes

First, 115 *CRGs* of *Arabidopsis* were employed in searches against rice protein sequences using the Blastp program with an *E*-value threshold of 1 × 10^−10^. The top-ranked rice hit was used to construct the *CRG* library combined with 115 *Arabidopsis CRGs*. Then, homologous *CRGs* in other species were identified through comparison with the *Arabidopsis* and rice *CRG* library by using the Blastp program (*E*-value < 1 × 10^−10^, identity > 60%) according to a previous report^60^. The Pfam (v32.0, http://pfam.xfam.org) database and a related Perl script were used to identify the *NBS* gene family^61^. Pfam accession number PF00931.18 was used to identify the NB-ARC domain. The genes were further verified with the SMART program^62^.

### Identification of gene collinearity and specific duplication events

BLAST and the Multiple Collinearity Scan toolkit (MCScanX) were used for gene collinearity analysis with default parameters according to previous reports^25,63^. Venn diagrams were drawn by using the R (v3.5.0) program VennDiagram. A program (duplicate_gene_classifier) in MCScanX was used to infer different types of duplicated genes. To identify the cold-related genes associated with a specific polyploidization event, we inferred colinear genes. First, BlastP was used to search for potential anchors (*E*-value < 10^–5^; top five matches) between each possible pair of chromosomes in multiple genomes. A loose *E*-value threshold accommodates the highly divergent evolutionary rates of duplicated genes produced by polyploidization millions of years ago.

The protein sequences from each species were searched against its own or other genome sequences. The best, second best, and other gene pairs were indicated by red, blue, and gray dots, respectively. Then, the homologous blocks within each genome and between different species (maximal gap ≤ 50 genes; *P*-value < 0.05) were determined using ColinearScan^64^. The key parameter of the maximum gap between neighboring genes along a chromosome showing collinearity with genes along the counterpart chromosome sequence was set to 50 intervening genes, which was proven to be successful in previous studies^29^.

### Orthologous and paralogous *CRG* identification

Orthologous and paralogous *CRGs* in *B. napus* and other species were identified using OrthoFinder (v2.2.7, https://github.com/davidemms/OrthoFinder/releases). The relationships between orthologous and paralogous genes were plotted using Circos (v1.0)^65^. Clustering was conducted by using the Markov cluster algorithm (MCL, v1.0, −*I* > 1.5).

### Gene expression analyses of *CRGs*

We conducted *CRG* expression analyses in *B. rapa* using RNA-Seq data reported previously (Tong et al.^66^). These data came from six tissues, including the roots, stems, leaves, flowers, siliques, and calli, and fragments per kilobase of transcript per million fragments mapped (FPKM) values were used for comparative analyses. The expression values of these *CRGs* in *Arabidopsis* were obtained from the eFP browser (http://bar.utoronto.ca/efp_arabidopsis/cgi-bin/efpWeb.cgi) according to a previous report^37^; these values came from two tissues, the shoots and roots, under different cold treatments (0, 0.5, 1.0, 3.0, 6.0, 12.0, and 24.0 h). The expression values of these *CRGs* in seven different tissues, including the roots, stems, leaves, shoots, flowers, pollen, and seeds, were also downloaded from the *Arabidopsis* eFP browser. The heat maps of hierarchical clustering according to gene expression were plotted using Tree View software (http://jtreeview.sourceforge.net/).

### Construction of regulatory networks

The interaction network of *CRGs* in *Arabidopsis* was constructed by using *Arabidopsis* Interactions Viewer (v2.0) (http://bar.utoronto.ca/interactions/cgi-bin/arabidopsis_interactions_viewer.cgi). The interaction networks related to orthologs of *Arabidopsis* genes in other species were constructed with Gephi (v0.8.2) software using the Force-based algorithm ForceAtlas^33^. The plug-ins of Multi Color Renderer were used to illustrate the WGD/WGT events (α, β, γ, and θ). The key *CRG* network for *Arabidopsis* was constructed by using the String databases (v10.5)^67^.

### Evaluation of network robustness

The robustness criterion is defined as *R* = *C/(N*−*Nr)*, where *N* is the size of a linked group or network defined by the number of nodes within it; *Nr* is the number of removed nodes; and *C* is the size of the largest linked group after the removal of those *Nr* nodes^68^. In our implementation, certain numbers or percentages of nodes were randomly removed from a certain network, and *R* was then calculated. This process was implemented 100 times, and an average *R* value was used to describe the robustness of the network.

### Selective pressure estimation

To estimate the divergence times of the colinear *CRG* pairs, the protein sequences were aligned with the ClustalW2 program and translated into the coding sequence alignment. The synonymous rate (Ks), nonsynonymous rate (Ka), and evolutionary constraint (Ka/Ks) between colinear gene pairs were calculated using the method of Nei and Gojobori, implemented in KaKs_Calculator (v2.0)^69^. The divergence times of our selected species were collected and integrated from several previous reports^70–72^. We employed the Codeml program from PAML (v4.9) and analyzed 16 key *CRG* families to calculate ω, which is the ratio of nonsynonymous to synonymous distances^73^, and we checked positive selection by employing a likelihood ratio test between the M7 and M8 models. Maximum-likelihood phylogenetic trees were constructed with the PhyML program (v3.0)^74^.

## Supplementary information

Suptext-SupFigureS1-S9

Supplementary tables 1-22
