# Supplementary material for: Preferential gene retention increases the robustness of cold regulation in Brassicaceae and other plants after polyploidization
Source: Hortic Res. 2020 Feb 21;7:20. doi: 10.1038/s41438-020-0253-0 (PMC7035258; doi:10.1038/s41438-020-0253-0)
Supplement: Supplementary file 1 — Suptext-SupFigureS1-S9 [file 41438_2020_253_MOESM1_ESM.pdf]

Supplementary Text

Disease resistance genes did not accumulate through polyploidization

To investigate whether polyploidization was the predominant mechanism by disease related genes, we investigated the *NBS* genes associated with disease resistance. In contrast, colinear regions were poor in *NBS* genes in all examined species (Table S6). The largest number of *NBS* genes were found in *M. truncatula* (565, 1.28% of annotated genes), followed by *P. trichocarpa* (563, 1.36%) and *O. sativa* (500, 0.90%), while the percentage of *NBS* genes was less than 0.50% in *B. napus, G. hirsutum*, *B. rapa*, and *M. acuminata* (Table S8). Similar to cold-related genes, *S. moellendorffii* (17, 0.08%) also contained the fewest NBS genes among all examined species. In comparison, *NBS* genes, key to plant disease resistance, expand largely by proximal and tandem duplications, evolving through a rapid birth-and-death process.

Interestingly, the pattern of propagation for *NBS* genes was precisely opposite of that for cold-related genes (Table S6). The ratio of *NBS* genes in colinear regions was significantly lower than that at the whole genome level in 18 of the 21 species, with *M. truncatula*, *A. trichopoda* and *S. moellendorffii* showing insignificant differences (Table S9). However, the percentage of *NBS* genes related to proximal or tandem duplication was significantly higher than the genome-wide average in nearly all species. We calculated the numbers of synonymous substitutions (Ks) for *NBS* genes (Table S22), and found that they often form young clusters with high similarity (Ks < 0.2) on chromosomes (Figure S8). For examples there are *NBS* gene clusters on chromosomes 1, 11, 14, and 17 of *P. trichocarpa*; and chromosomes 3, 7, and 9 of *G. raimondii* (Figure S8).

Supplementary Figure S1-S9

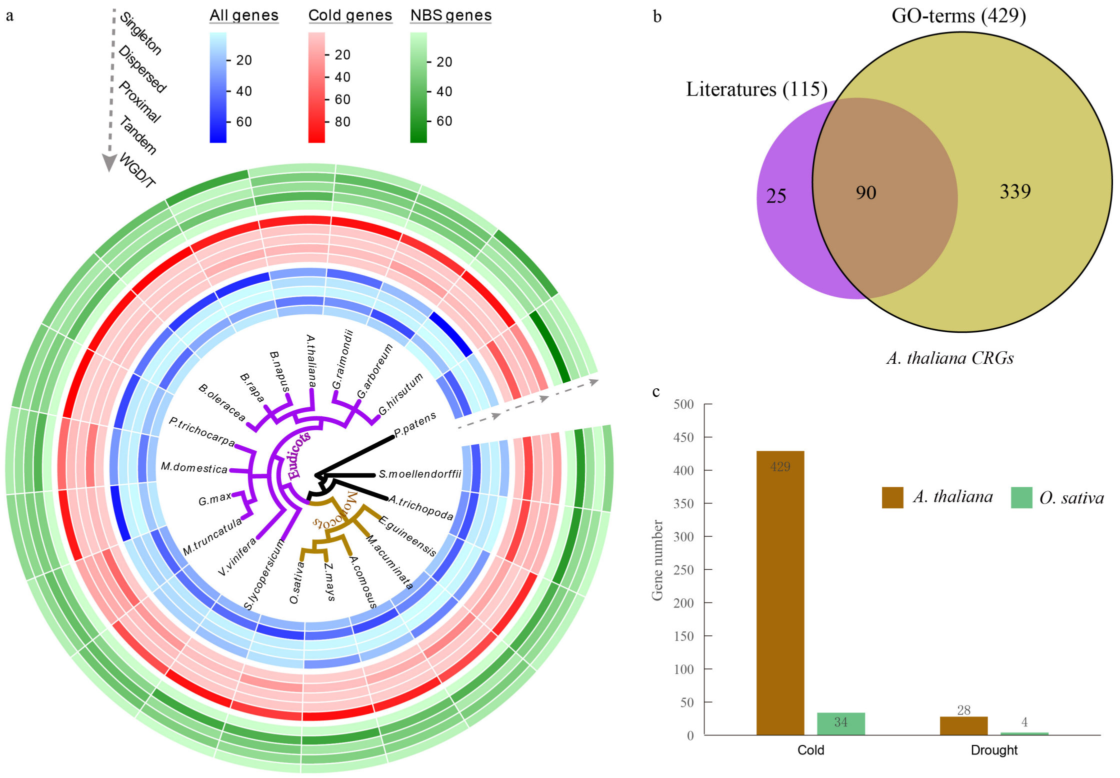

Figure S1. (a) The five duplicated types of cold-related genes (*CRGs*) and *NBS* genes. The percentages of each duplicated type for *CRGs* (red gradient), *NBS* genes (green gradient), and all genes (blue gradient) in the whole genomes of 21 plants. (b) The venn diagram showed the common and specific *CRGs* identified by the two methods (Literatures and GO terms). (c) The *CRGs* and drought-related genes identified by the GO-terms in *Arabidopsis* and rice.

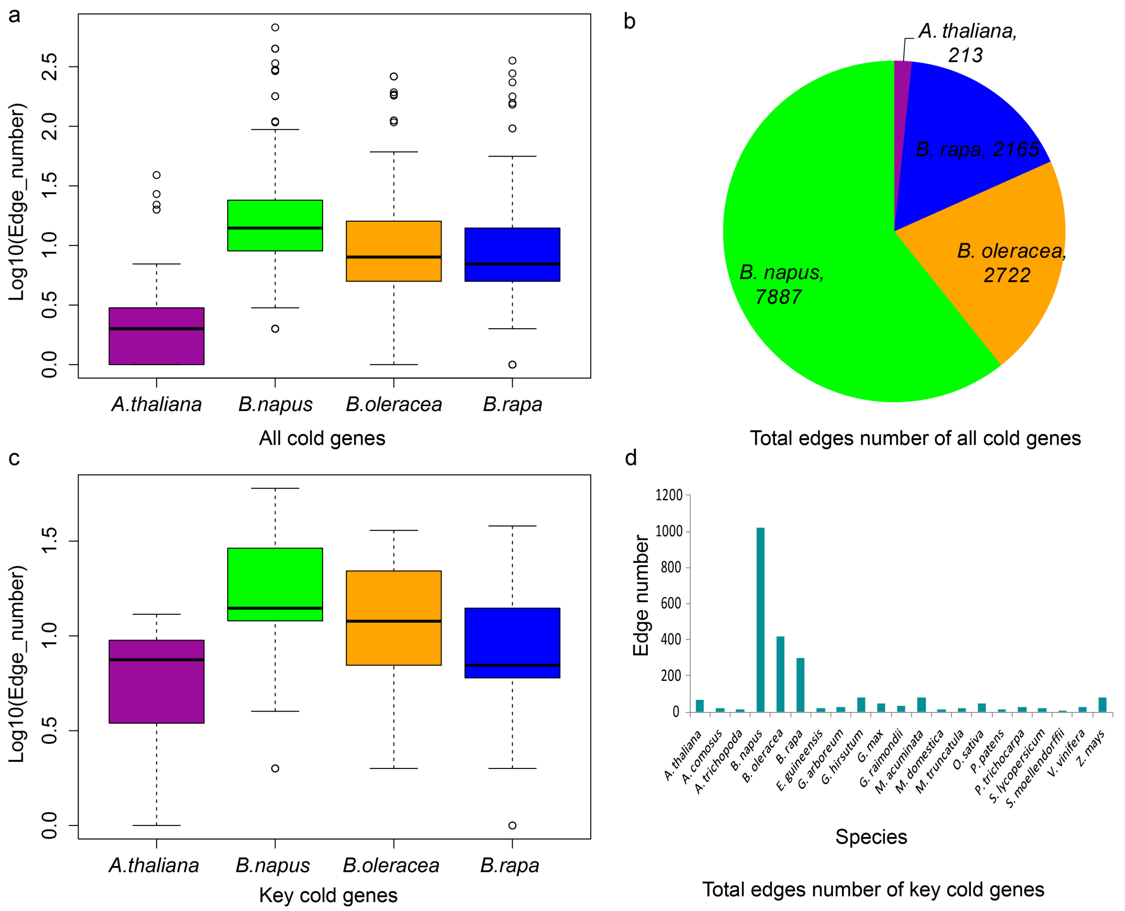

Figure S2. The statistics of edge number for interaction network. (a) The boxplot shows edge number (Log10) of the interaction network constructed by all cold-related genes in *A. thaliana*, *B. napus*, *B. oleracea*, and *B. rapa,* respectively. (b) The total edge number of the interaction network constructed by all cold-related genes in *A. thaliana, B. napus, B. oleracea,* and *B. rapa*, respectively (c) the boxplot shows edge number (Log10) of the interaction network constructed by key cold-related genes in *A. thaliana, B. napus, B. oleracea,* and *B. rapa,* respectively. (d) the total edge number of the interaction network constructed by key cold-related genes in 21 species.

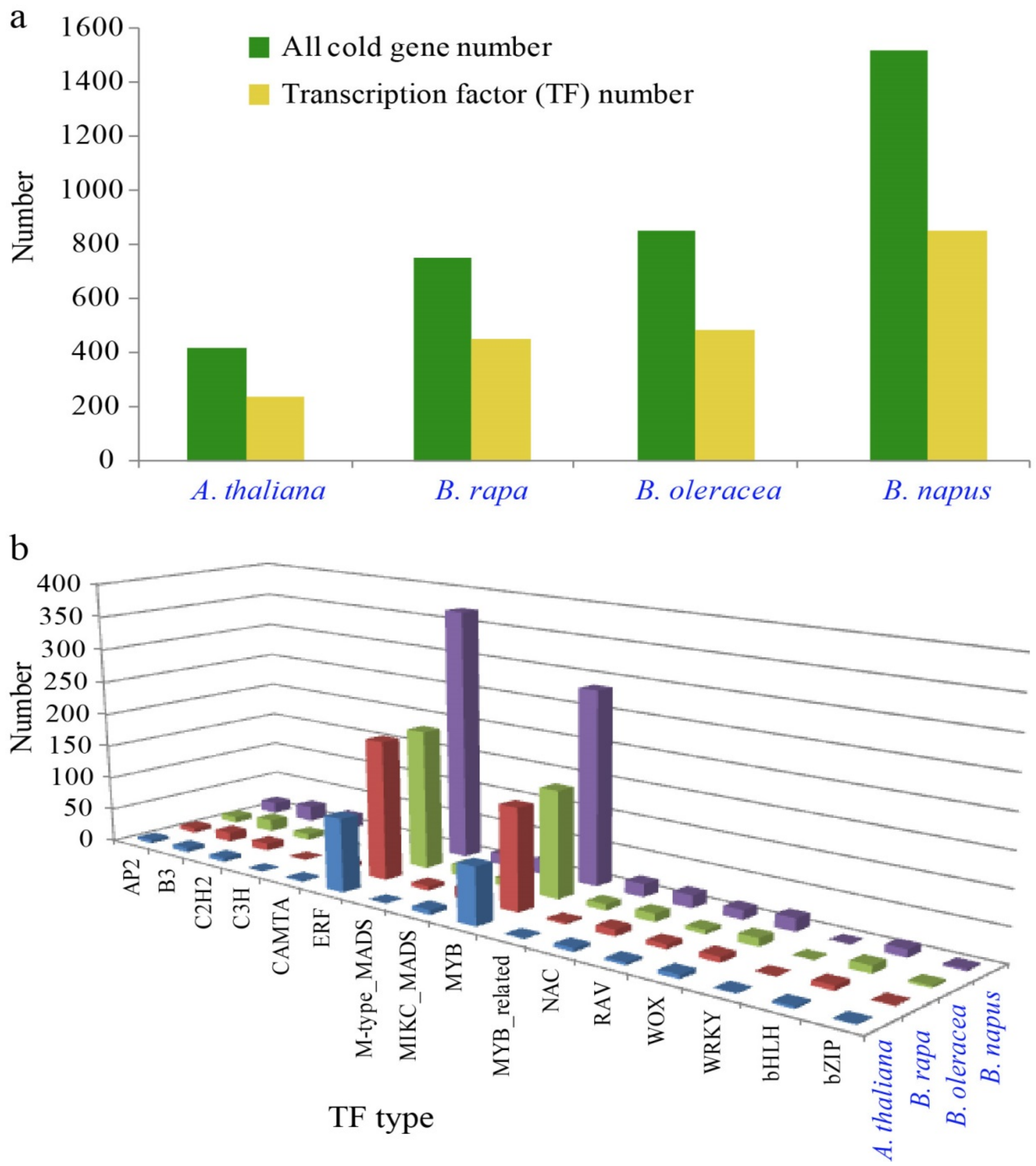

Figure S3. Identification of transcription factor from the cold-related genes (*CRGs*) in *A. thaliana, B. napus, B. oleracea,* and *B. rapa*, respectively. (a) The number of transcription factor identified from the *CRGs*. (b) The number of each transcription factor in *A. thaliana, B. napus, B. oleracea,* and *B. rapa*.

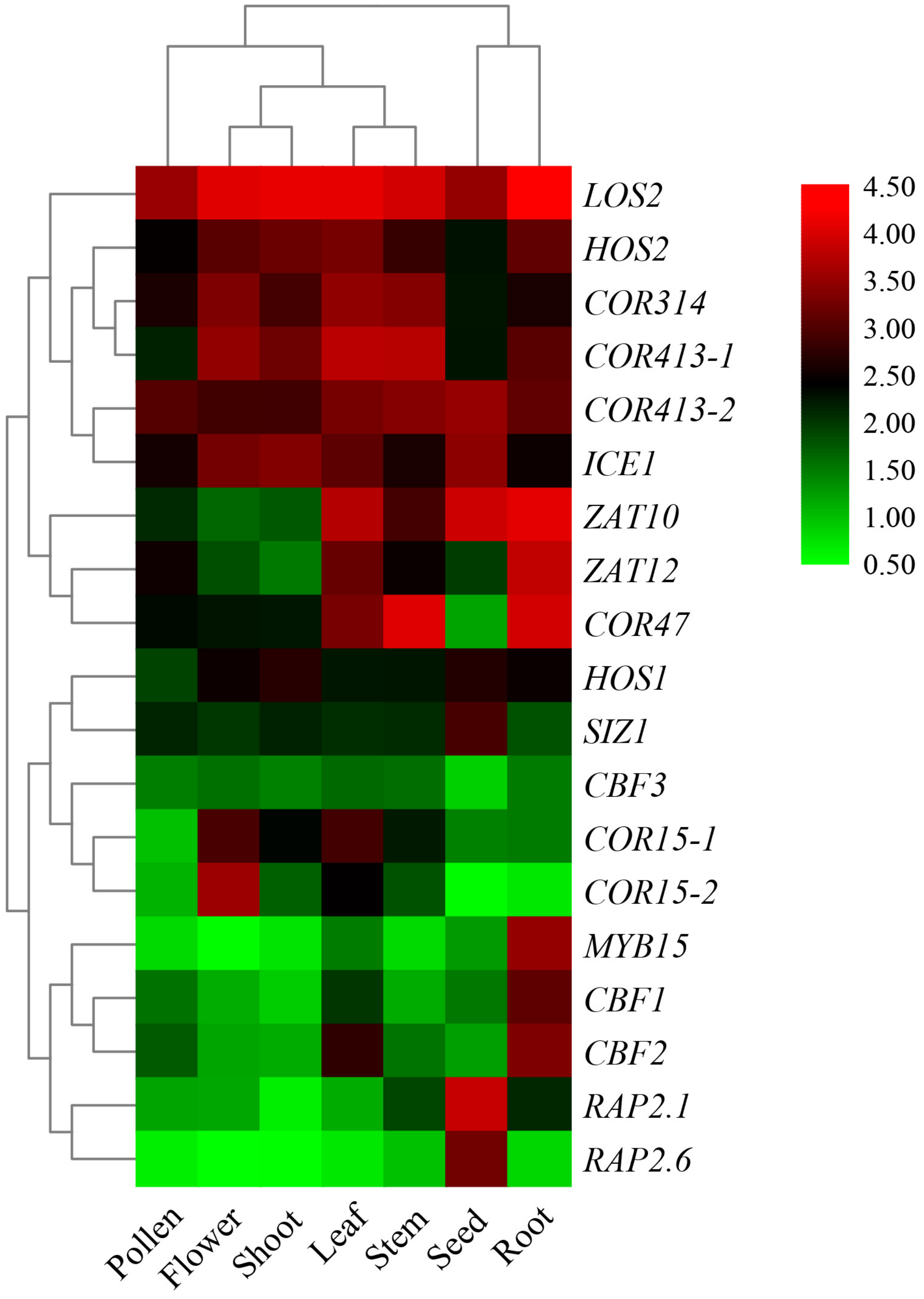

Figure S4. Expression cluster analyses for *Arabidopsis* key *CRGs* in different tissues, including root, stem, leaf, shoot, flower, pollen, and seed. The expression values were transformed by log2 for constructing the heatmap cluster analyses. The colors from green to red represent the gene expression from low to high level.

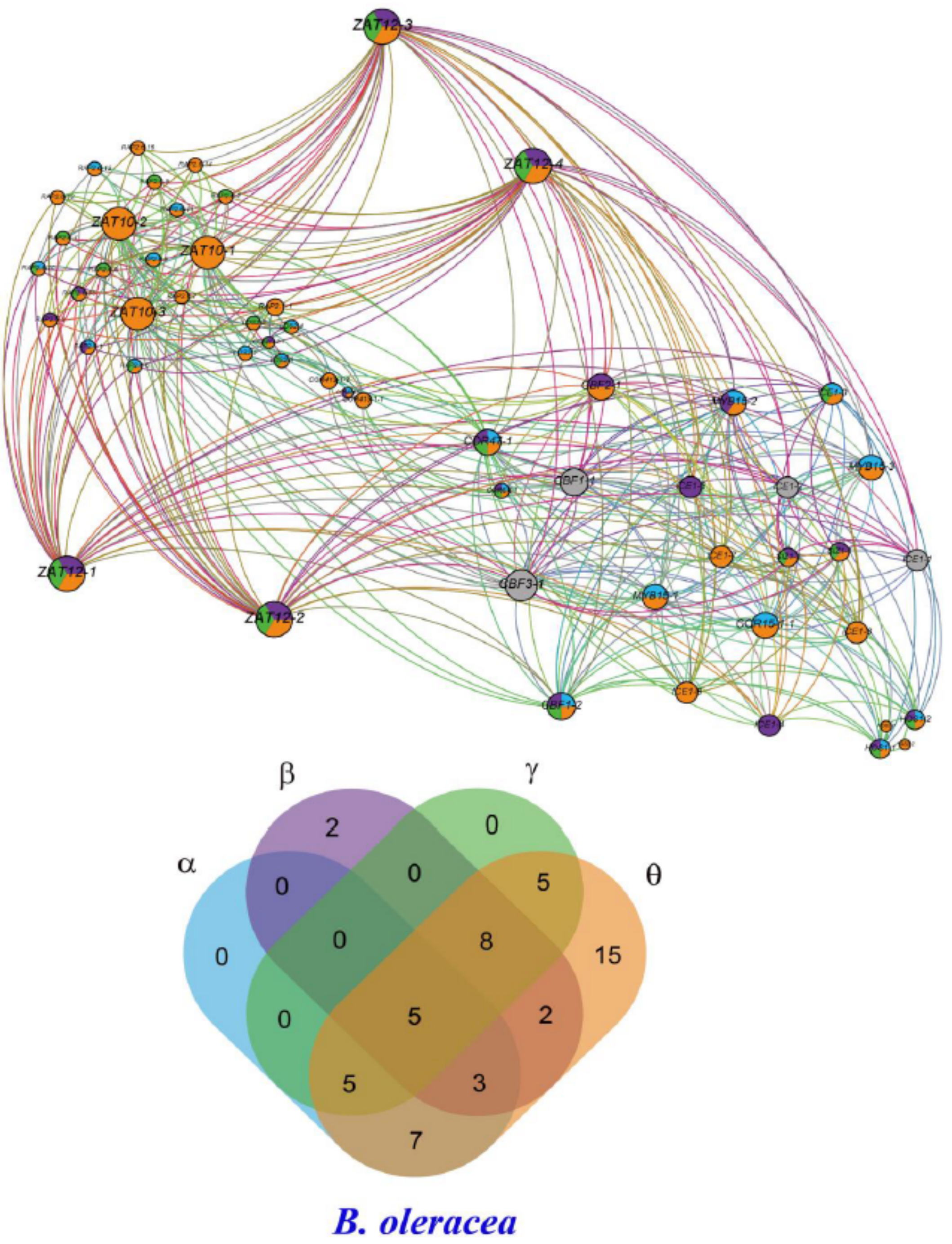

Figure S5. The interaction network analyses of key cold-related genes in 21 species (Part, please check the results of all species in the separate Figure S5 file). The Venn diagrams indicate numbers of common and specific key cold-related genes of different polyploidy events in *B. oleracea*.

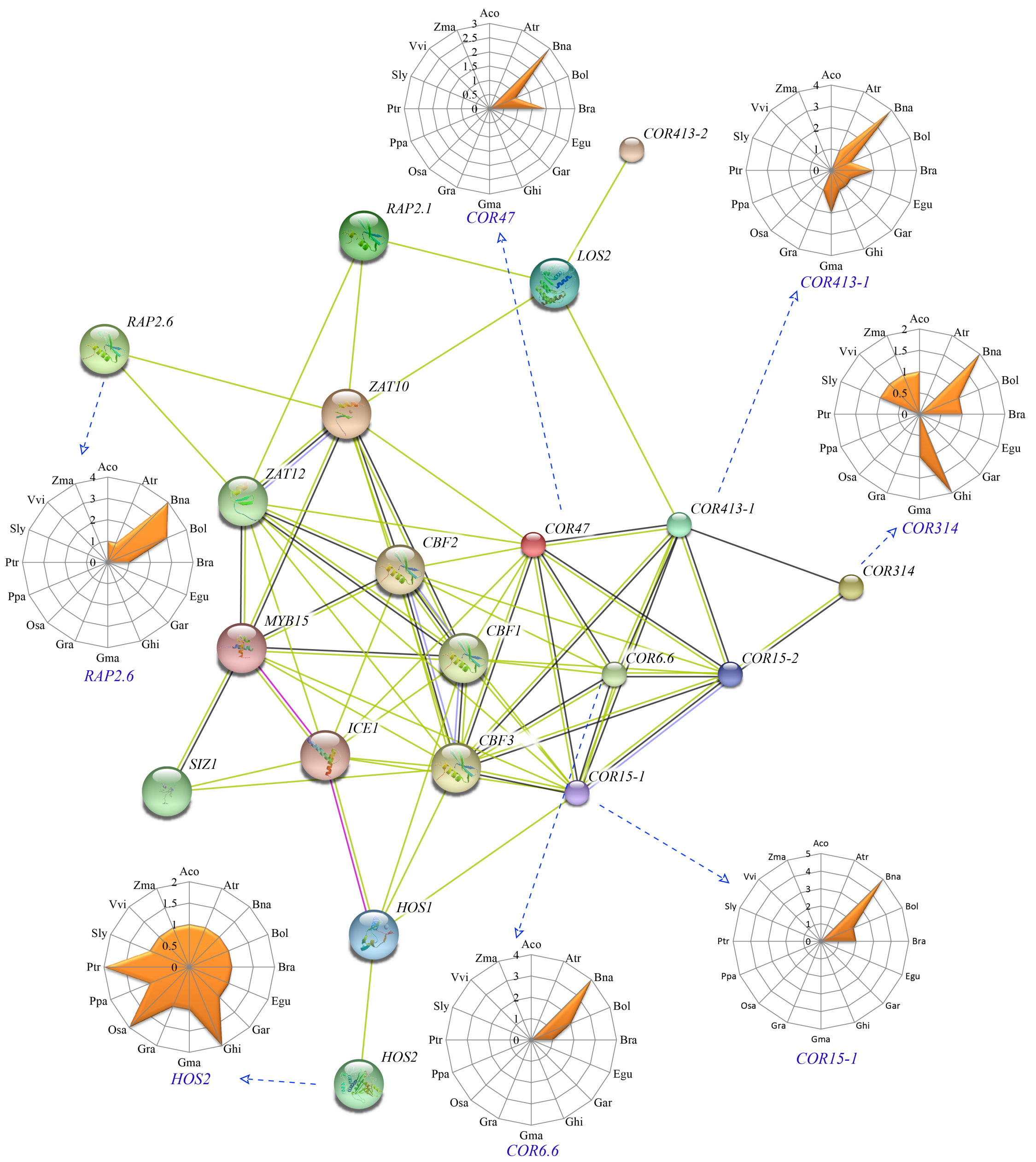

Figure S6. The number of inferred genes in each plant that are orthologous to key cold-related genes in *Arabidopsis*. The interaction network was constructed using the String database (https://string-db.org/).

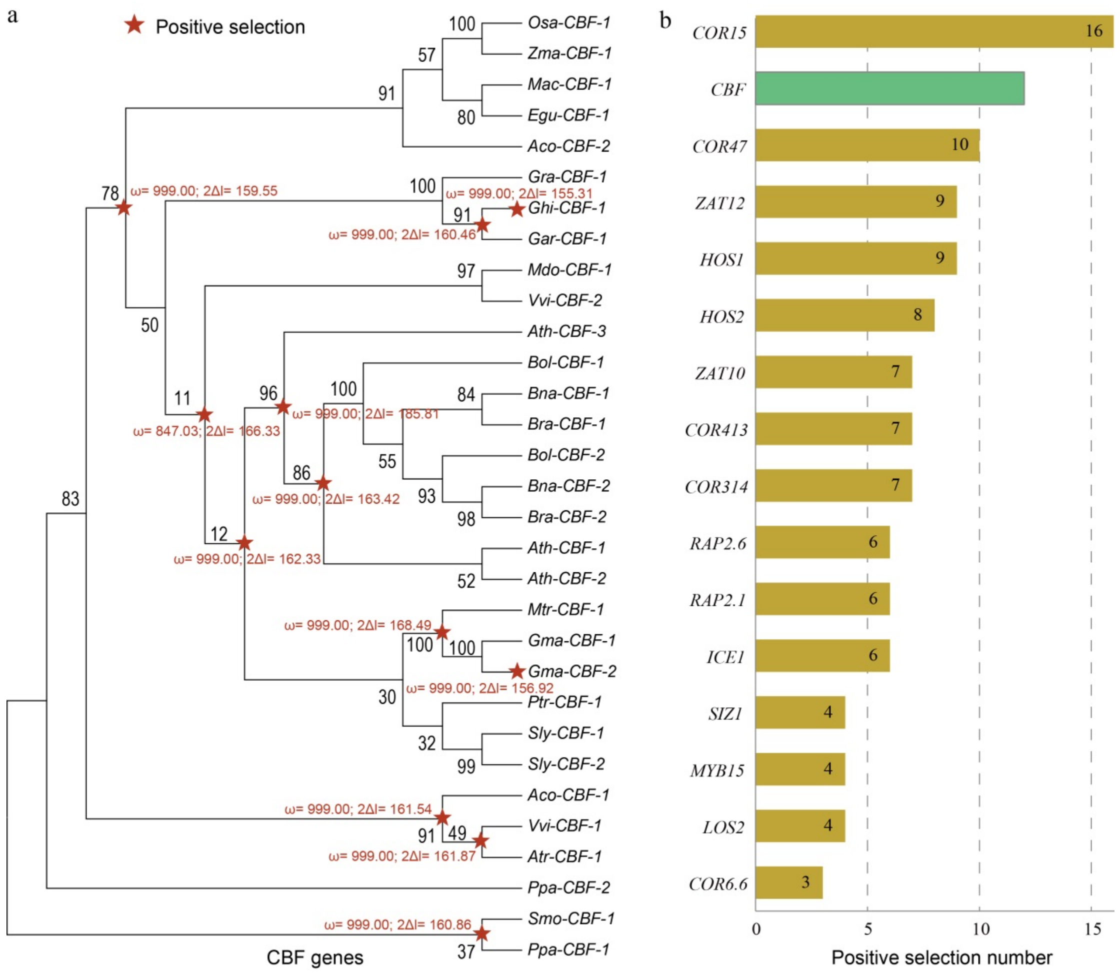

Figure S7. Phylogenetic relationships and positive selection analyses. (a) Phylogenetic trees constructed using ML method by PhyML, using the *CBF* genes in 21 species (Please check the results of all other key *CRGs* in the separate Figure S7 file). The numbers are bootstrap values based on 1,000 iterations. The ω on the clades is the dn/ds value under M8 of codeml, which represent the results of positive selection analyses. (b) Positive selection analyses for 16 cold-related gene families in representative species. The numbers indicate nodes with significant positive selection.

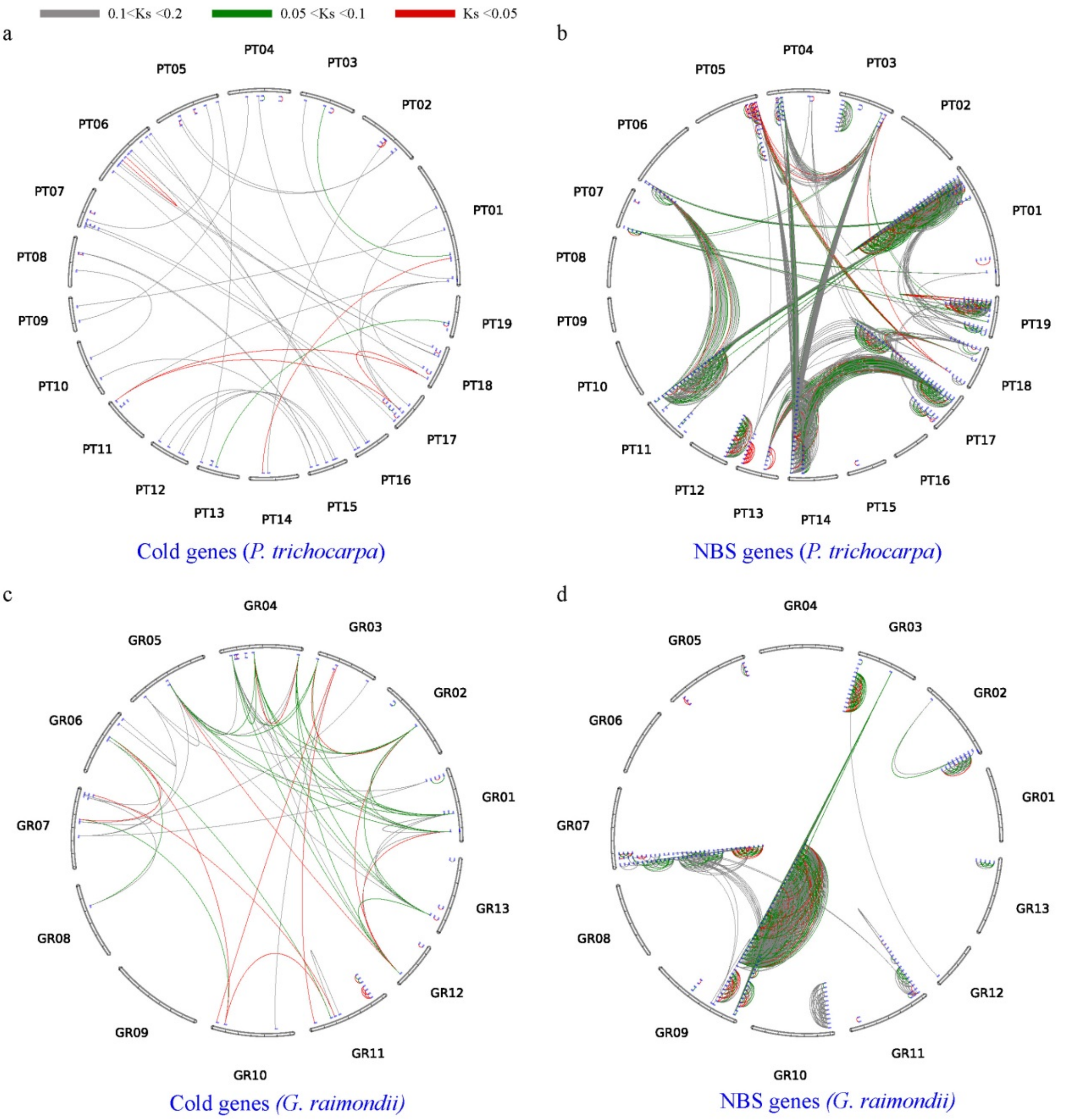

Figure S8. Ks analyses for cold-related genes (*CRGs*) and *NBS* genes in each species (Please check the results of all species in the separate Figure S8 file). Ks for (a) *CRGs* in *P. trichocarpa*; (b) *NBS* genes in *P. trichocarpa*; (c) *CRGs* in *G. raimondii*; (d) *NBS* genes in *G. raimondii*.

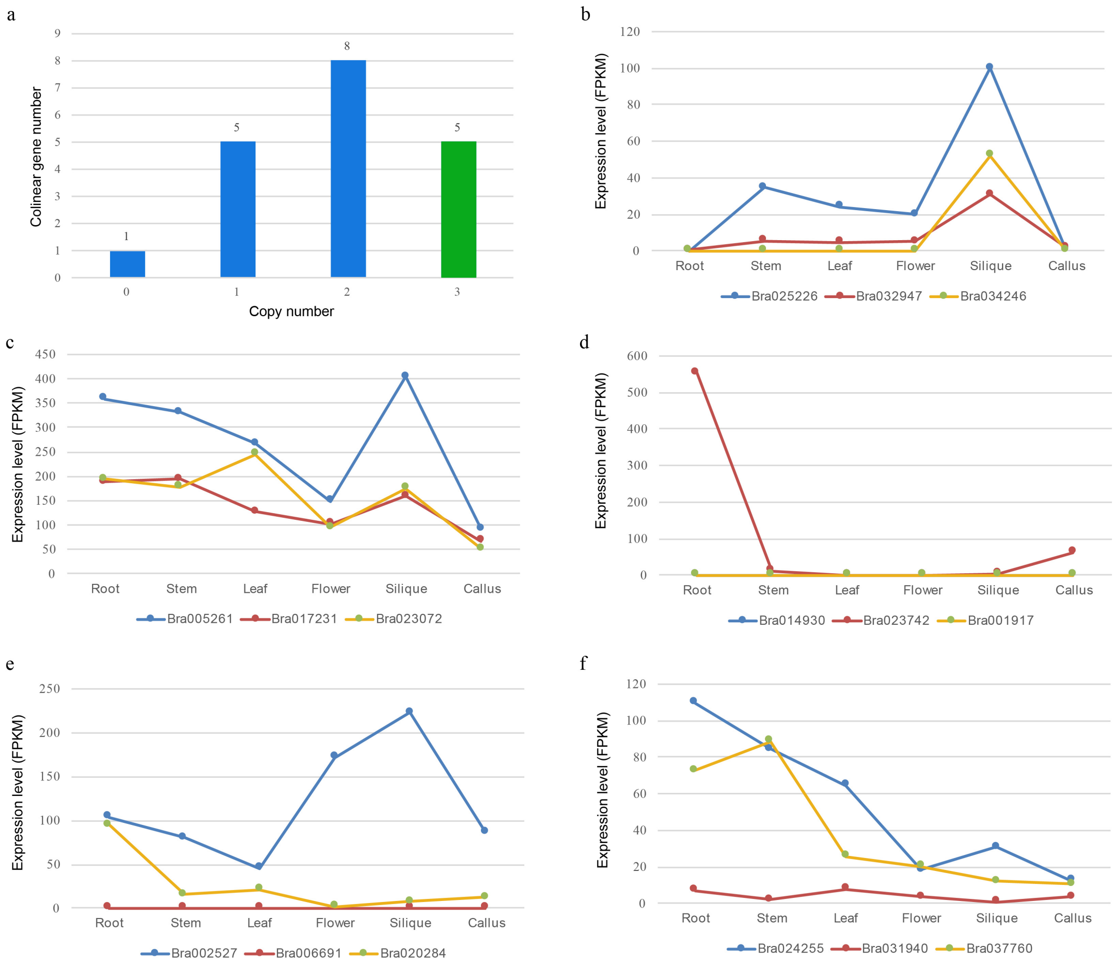

Figure S9. The expression level (FPKM) of collinear genes in *B. rapa* with *Arabidopsis* key cold related genes in 6 tissues. (a) The collinear gene number in *B. rapa* with *Arabidopsis* for copy number from 0 to 3. (b-f) The expression level (FPKM) of collinear genes with 3 copy number compared with *Arabidopsis* in *B. rapa*.

The large separated supplementary Figures

Figure S5. The interaction network analyses of key cold-related genes (*CRGs*). The Venn diagrams indicate the numbers of common and specific key *CRGs* of different polyploidy events.

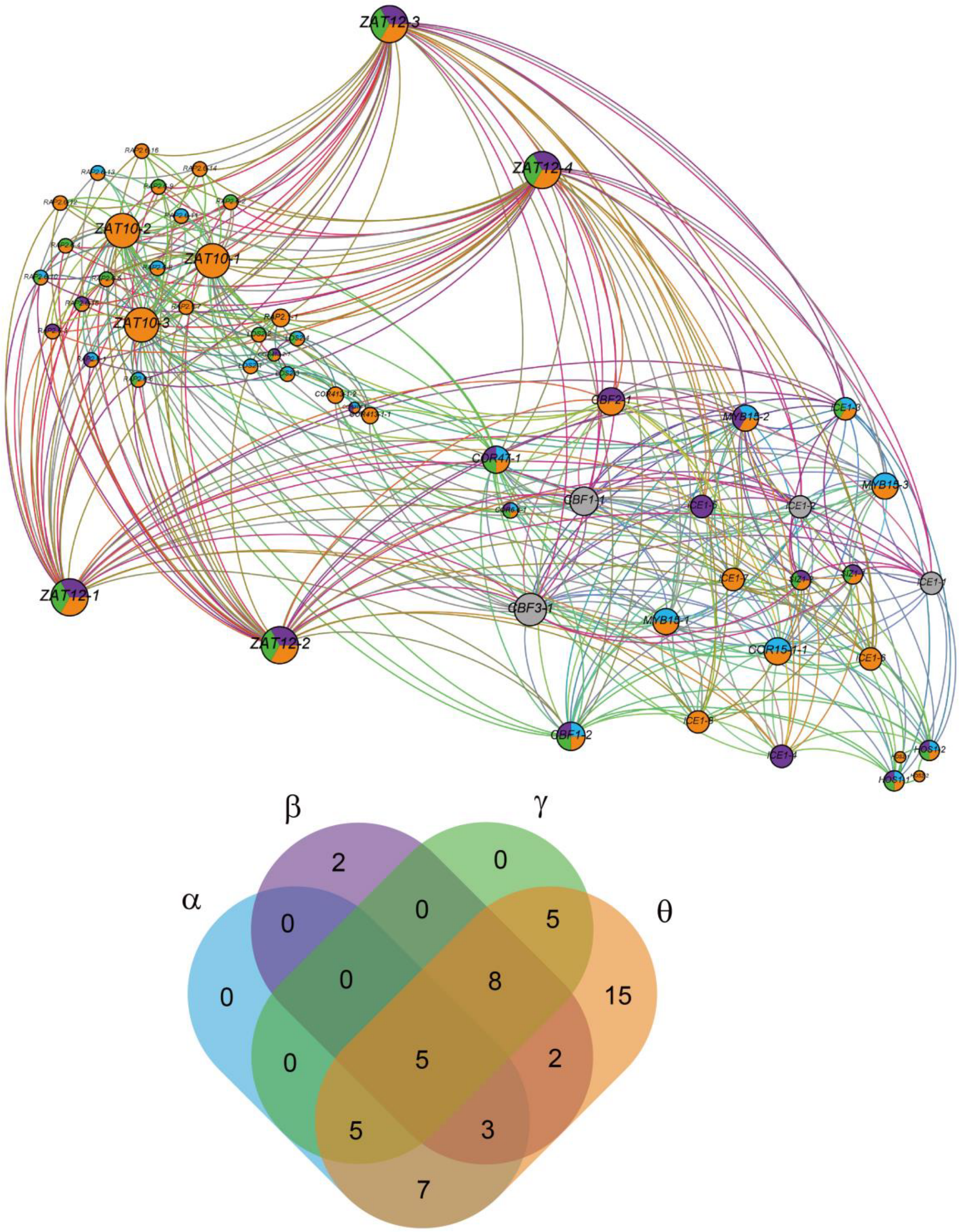
ZAT12-3
ZAT12-4
ZAT10-2
ZAT10-1
ZAT10-3
CBF2-1
MYB15-2
COR47-1
CBF1-1
MYB15-3
ZAT12-1
ZAT12-2
CBF3-1
MYB15-1
COR15a-1
CBF1-2
ICE1-1
ICE1-2
β
γ
α
θ
2
0
0
0
5
0
15
0
8
0
5
2
5
3
7

B. oleracea

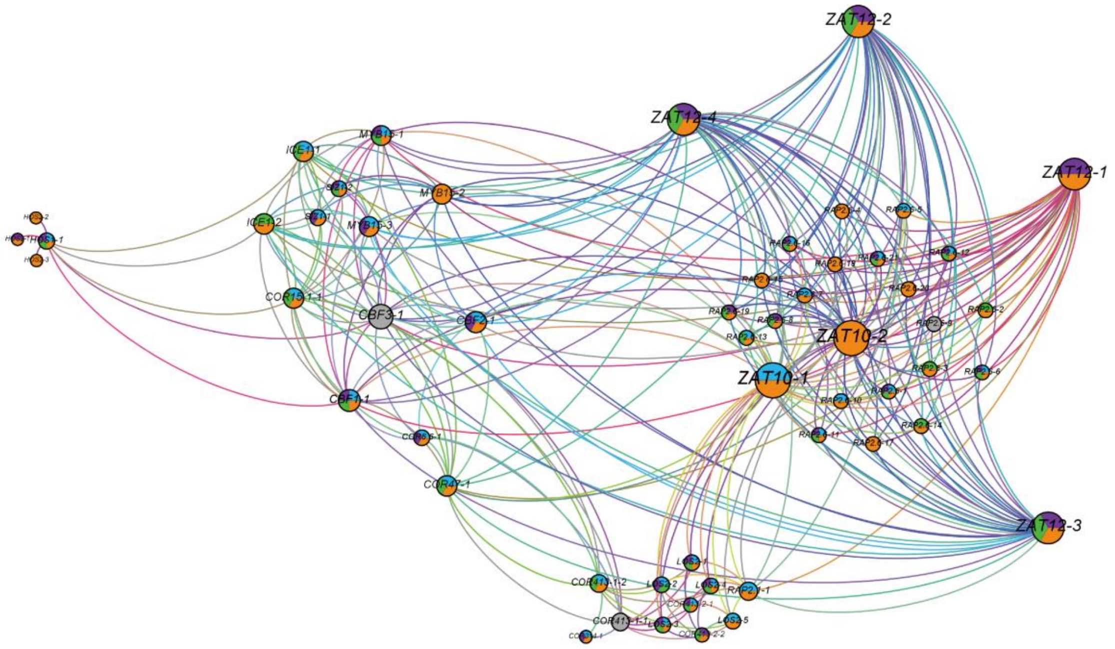
ZAT12-2
ZAT12-4
ZAT12-1
ZAT10-2
ZAT10-1
ZAT12-3
CBF3-1
ICE1-1
ICE1-2
MYB15-1
MYB15-2
MYB15-3
COR15-1-1
CBF1-1
COR47-1

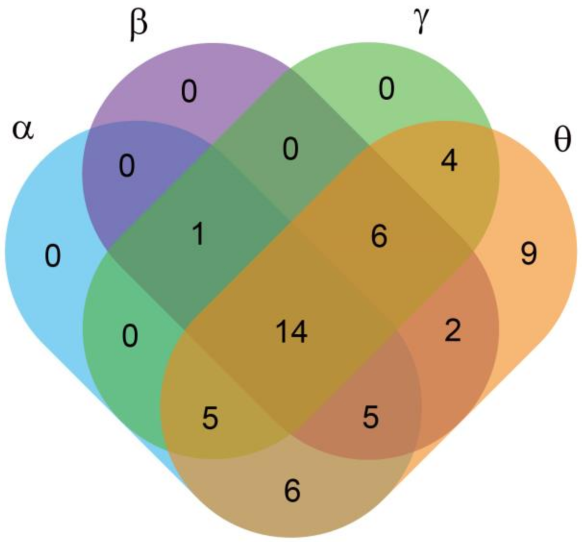
β
γ
α
θ
0
0
0
0
4
0
1
6
9
0
14
2
5
5
6

B. rapa

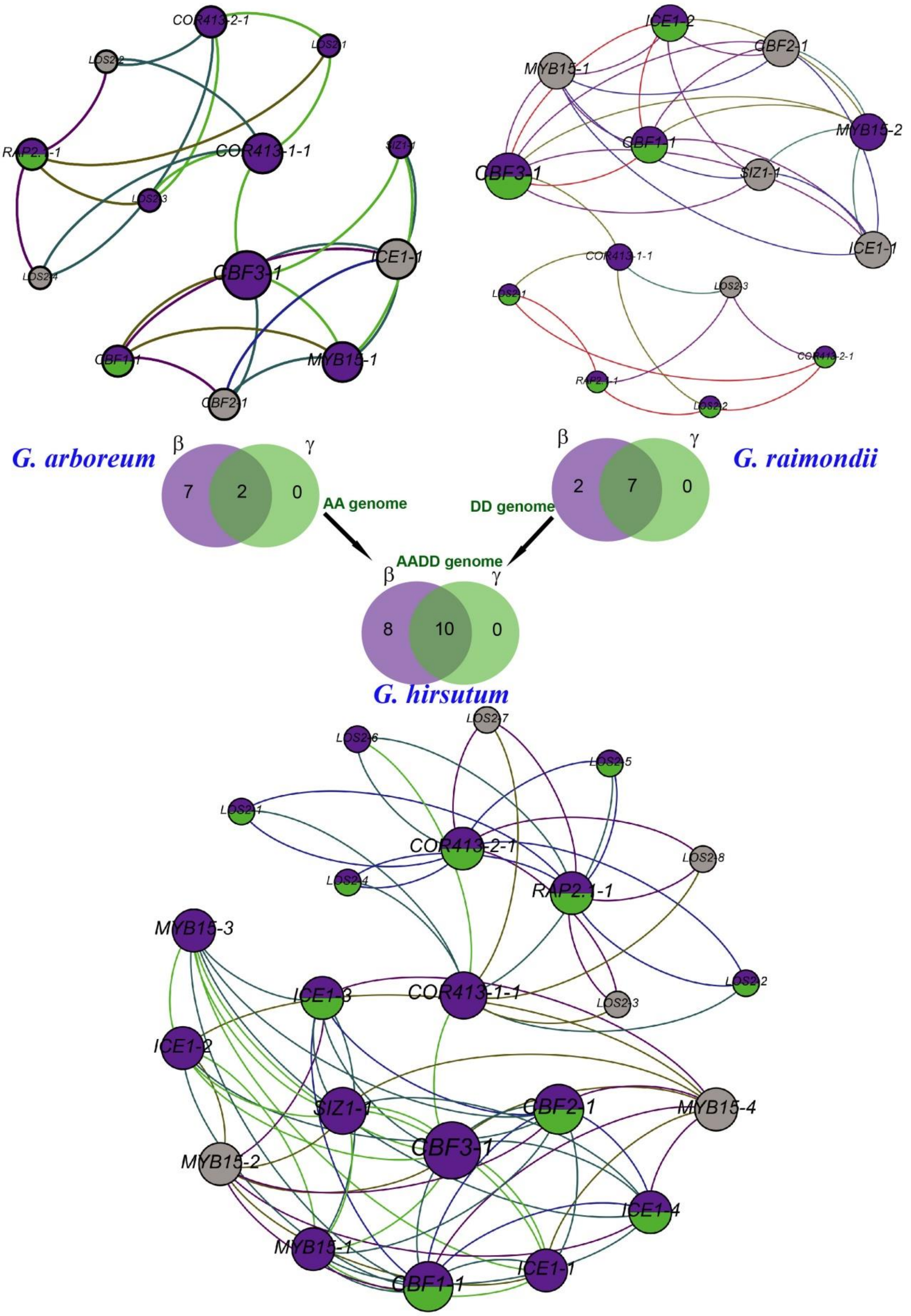
G. arboreum
G. raimondii
G. hirsutum
AA genome
DD genome
AADD genome
β
γ
7
2
0
8
10
COR413-2-1
LOS2-1
LOS2-2
LOS2-3
LOS2-4
RAP2.1-1
COR413-1-1
SIZ1-1
CBF3-1
ICE1-1
CBF1-1
MYB15-1
CBF2-1
ICE1-2
MYB15-2
MYB15-3
MYB15-4
LOS2-5
LOS2-6
LOS2-7
LOS2-8
ICE1-3
ICE1-4

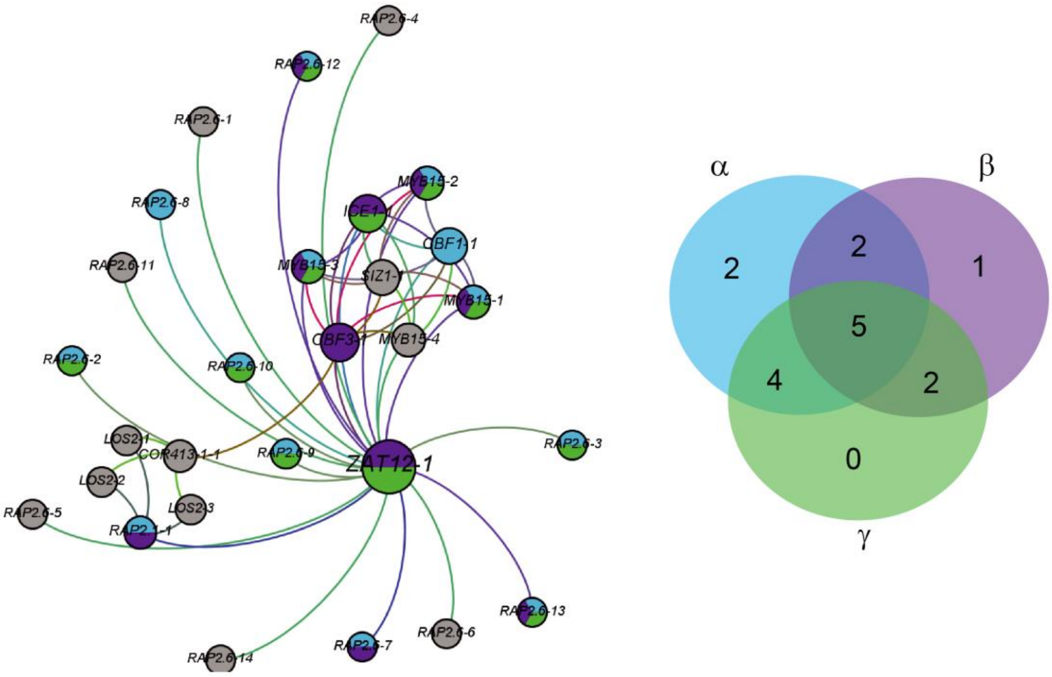

O. sativa

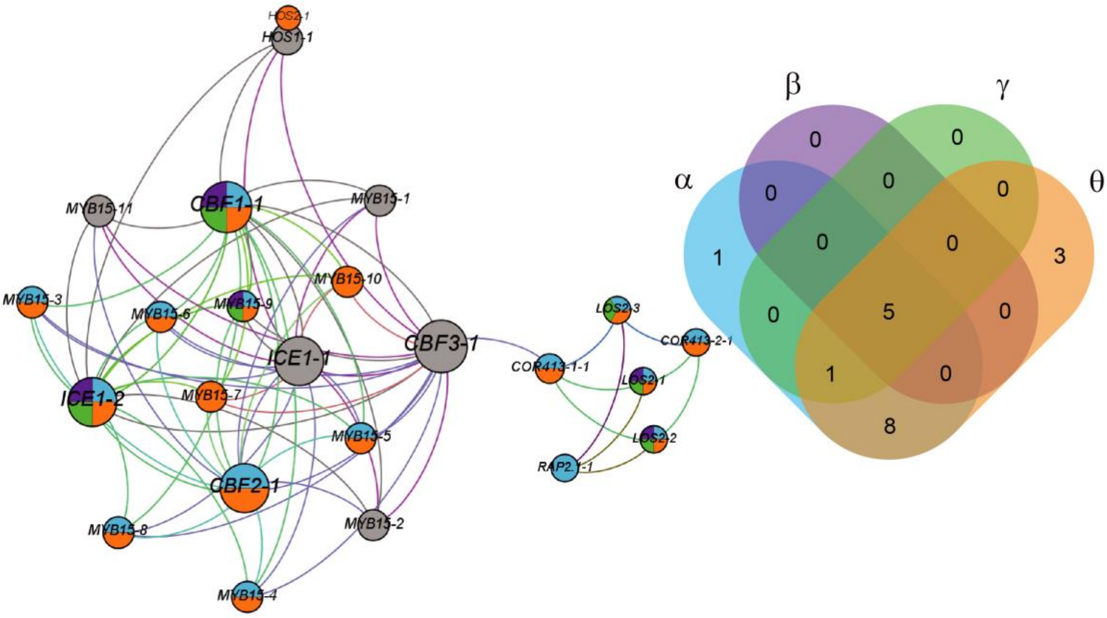

Z. mays

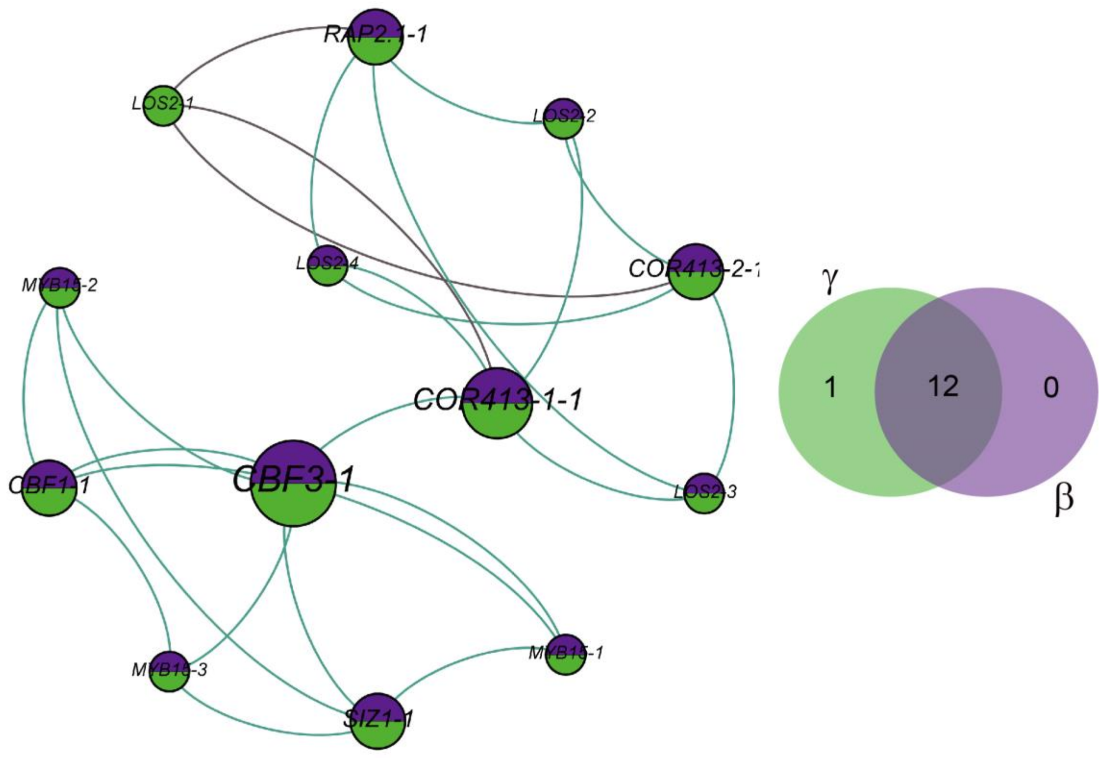

P. trichocarpa

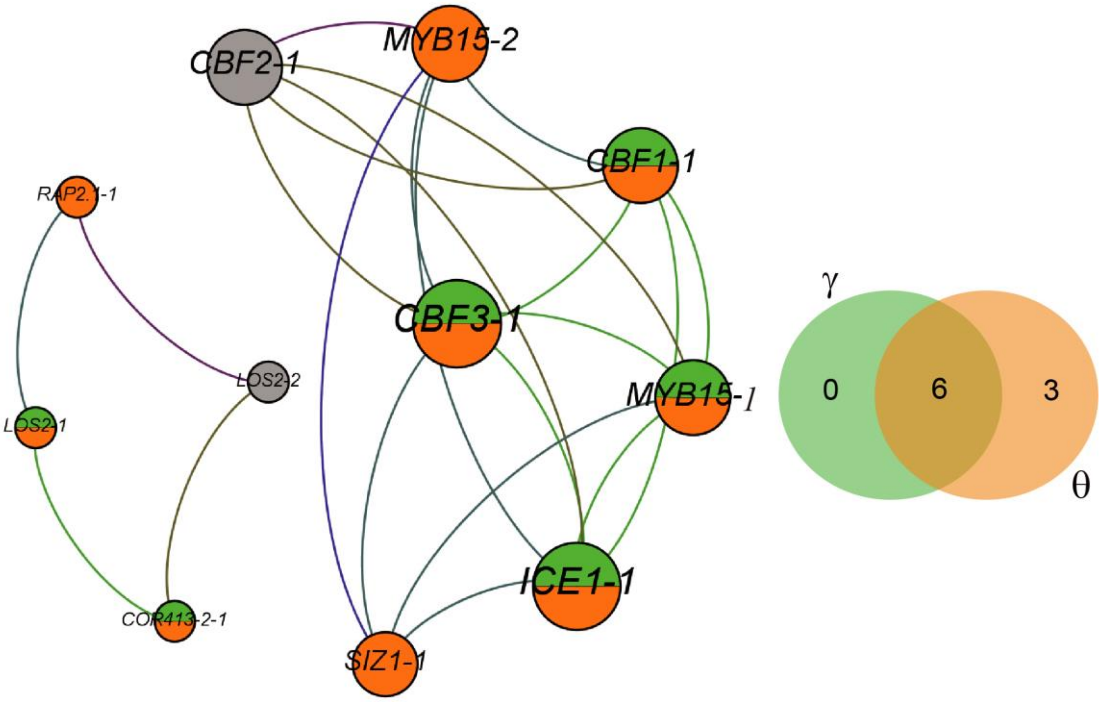

S. lycopersicum

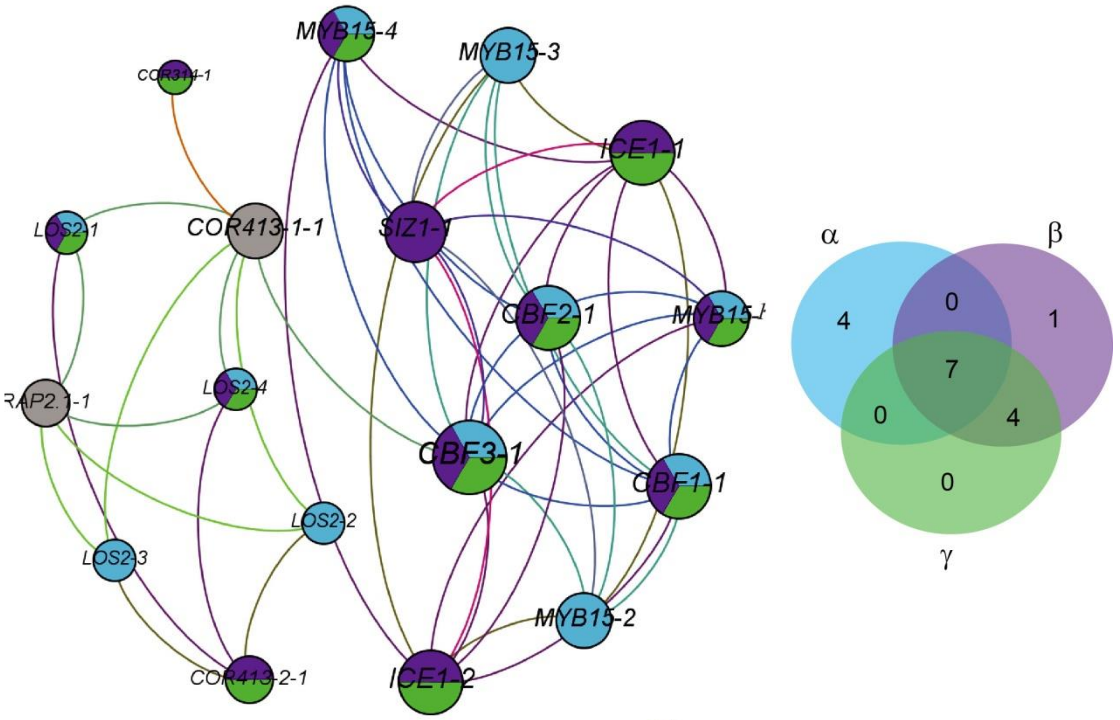

G. max

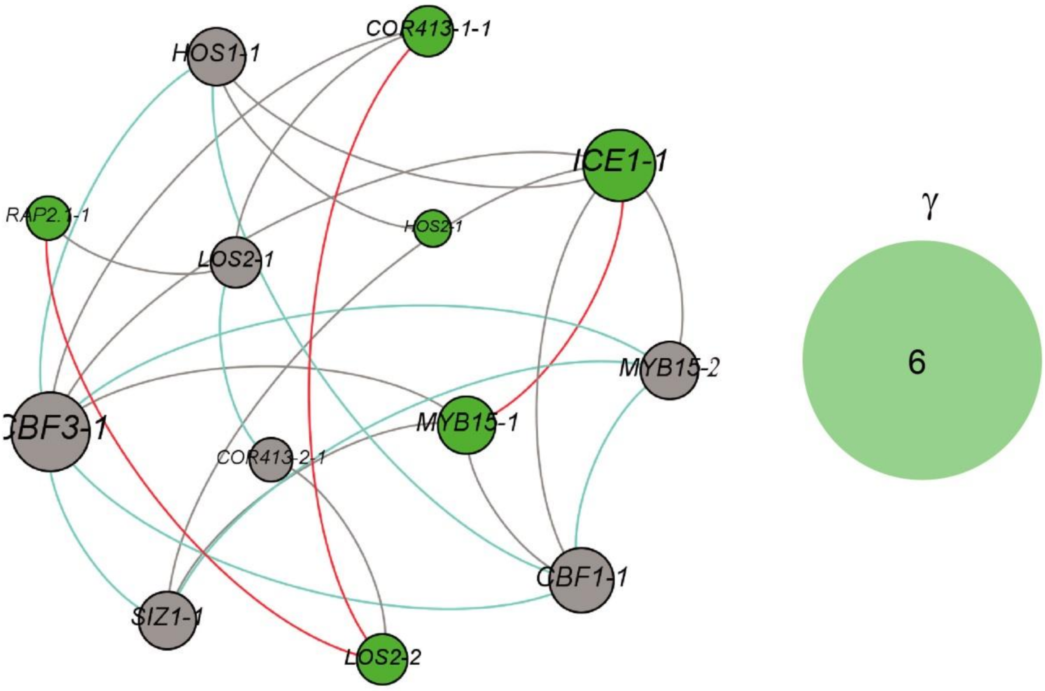

V. vinifera

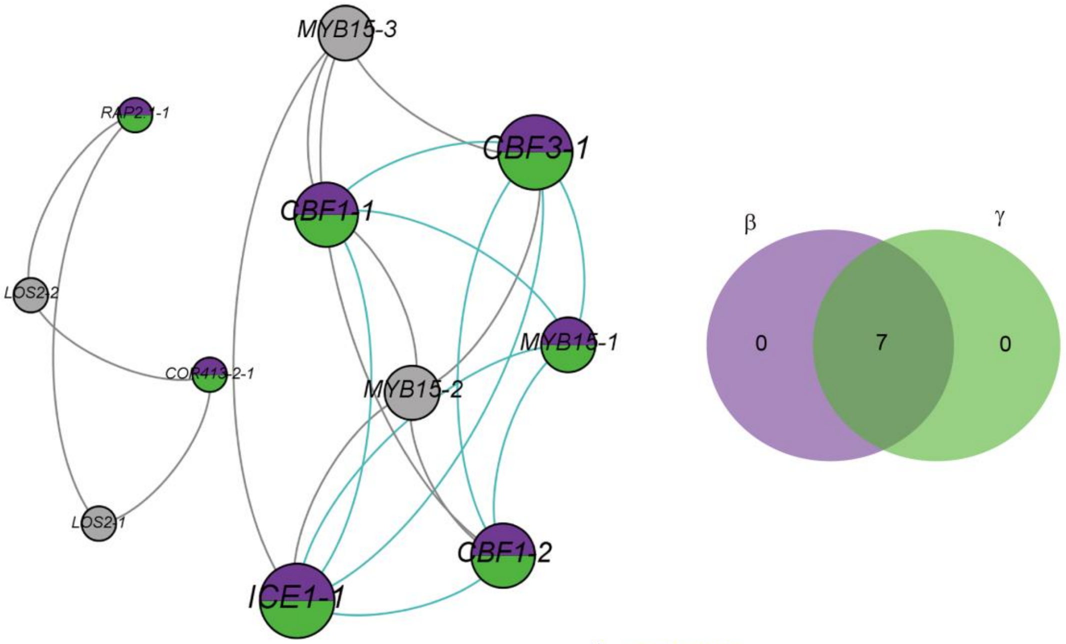

A. comosus

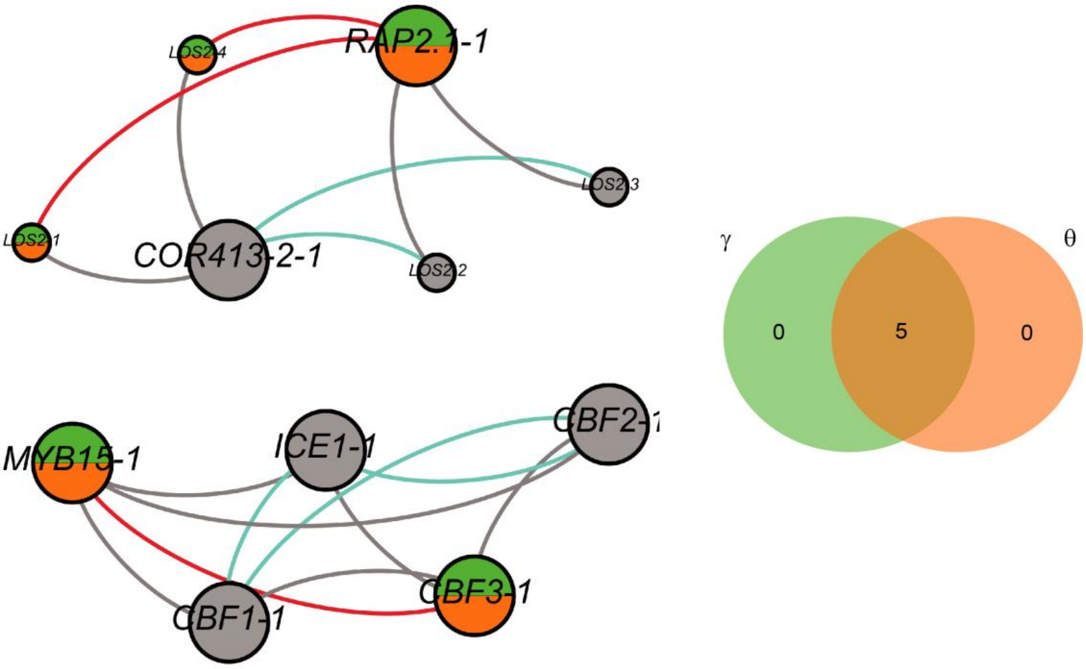

E. guineensis

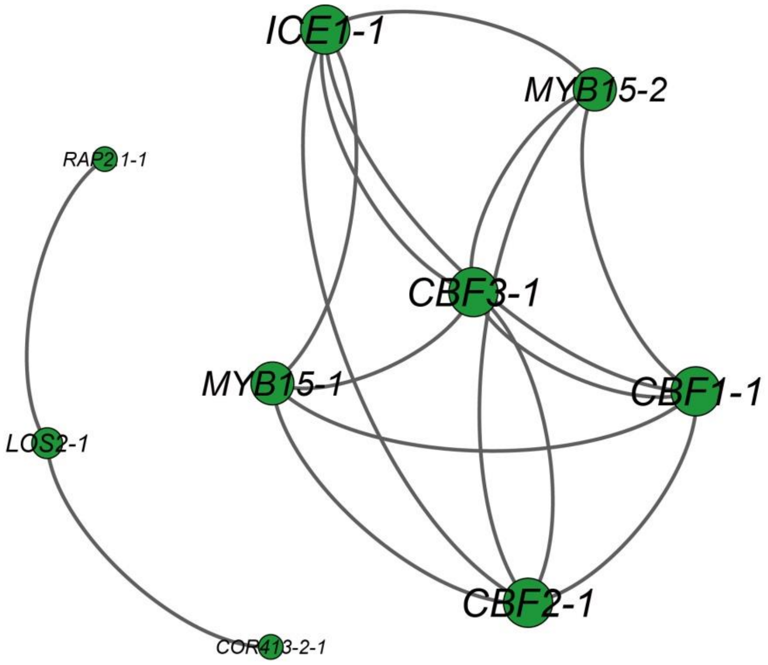

A. trichopoda

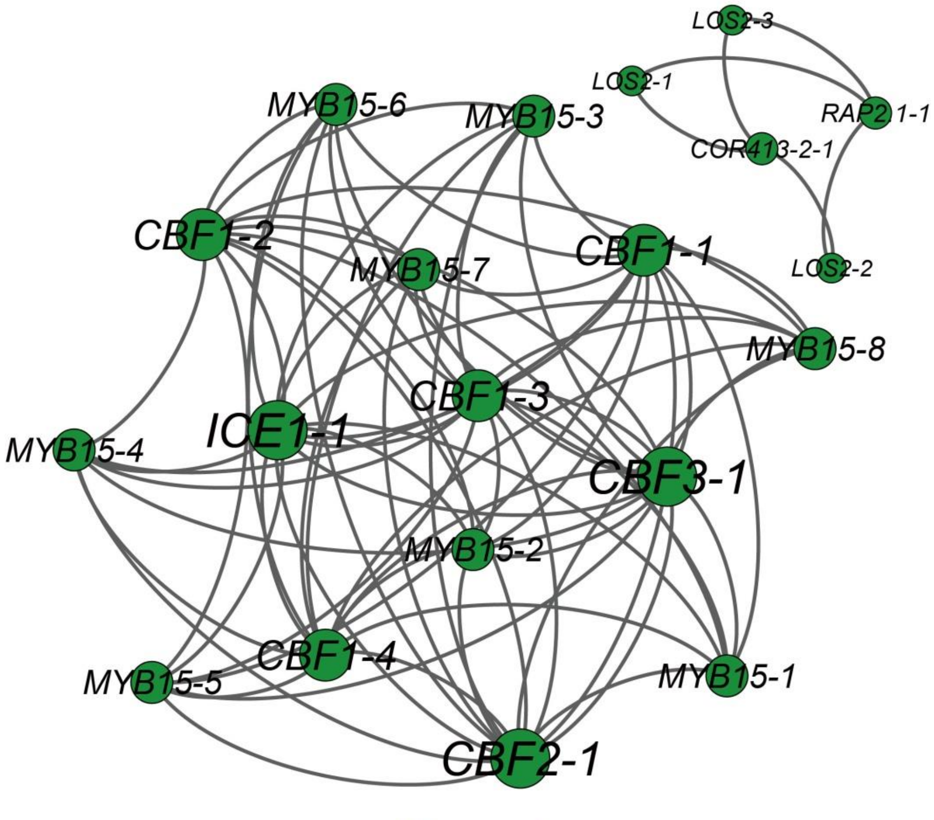

M. acuminata

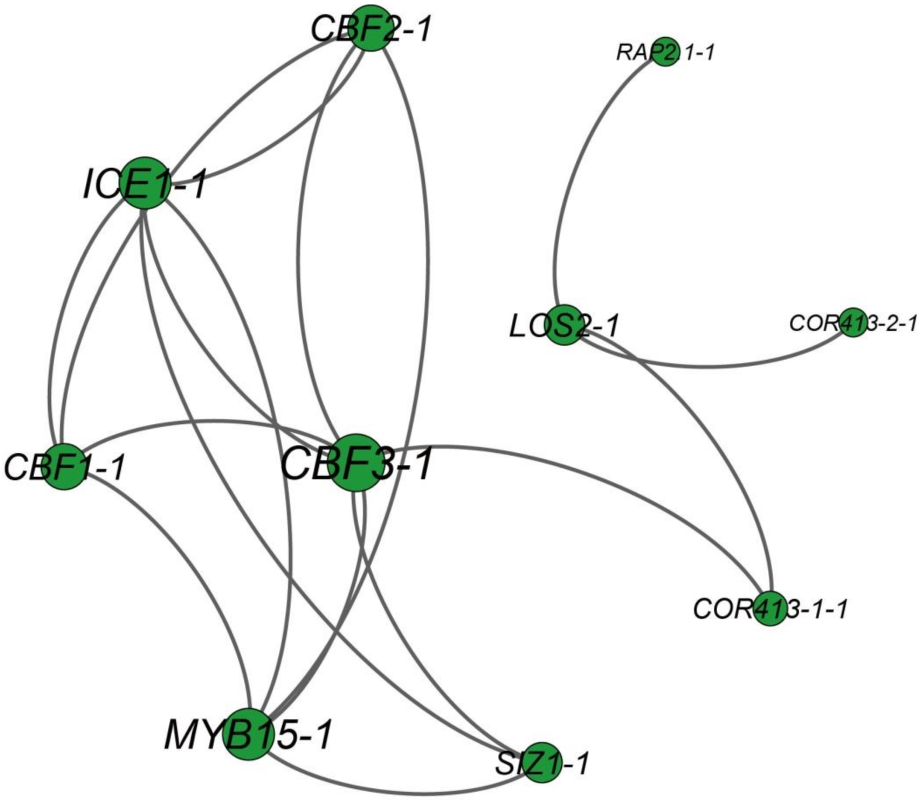

M. truncatula

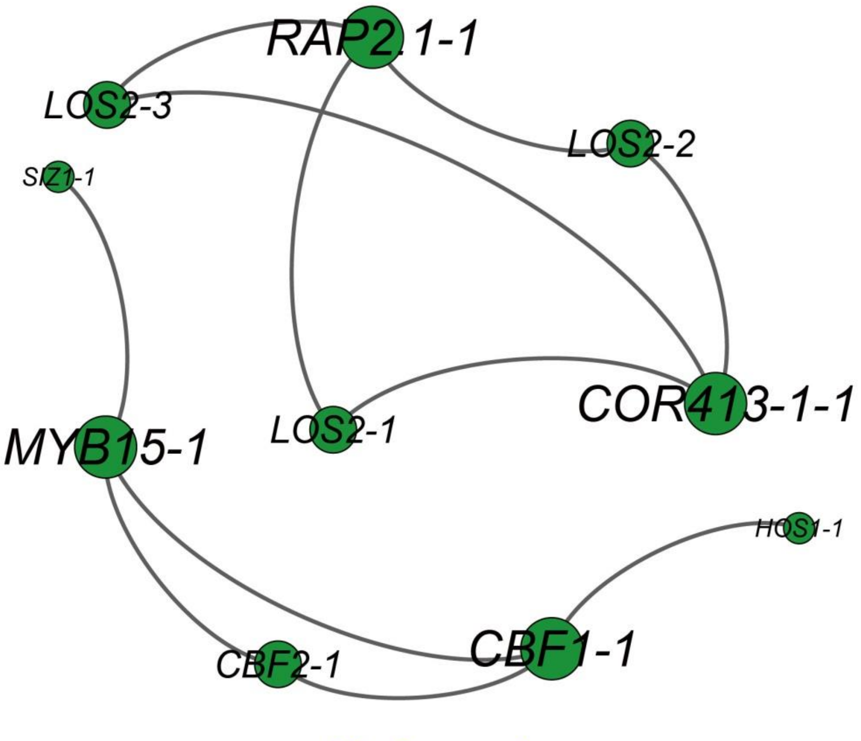

M. domestica

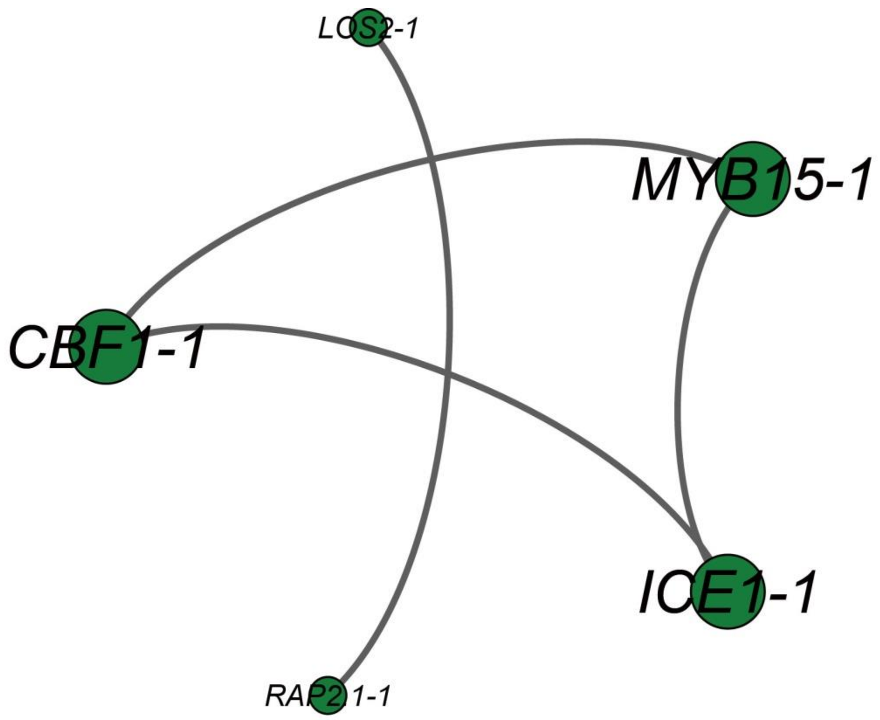

S. moellendorffii

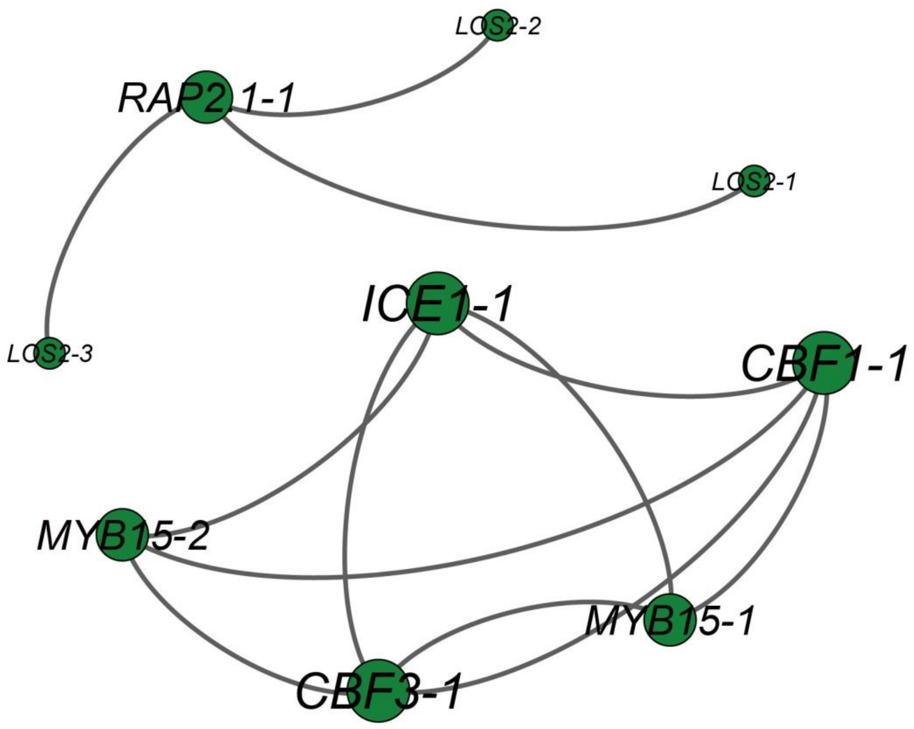

P. patens

The large separated supplementary Figures

Figure S7. The phylogenetic relationship and positive selection analyses. Phylogenetic trees constructed using the ML method by PhyML, using the 16 key cold-related genes in 21 species. The numbers are bootstrap values based on 1,000 iterations. The positive selection was conducted for key cold-related genes. The ω on the clades is the dn/ds value under M8 of codeml, which represent the results of positive selection analyses.

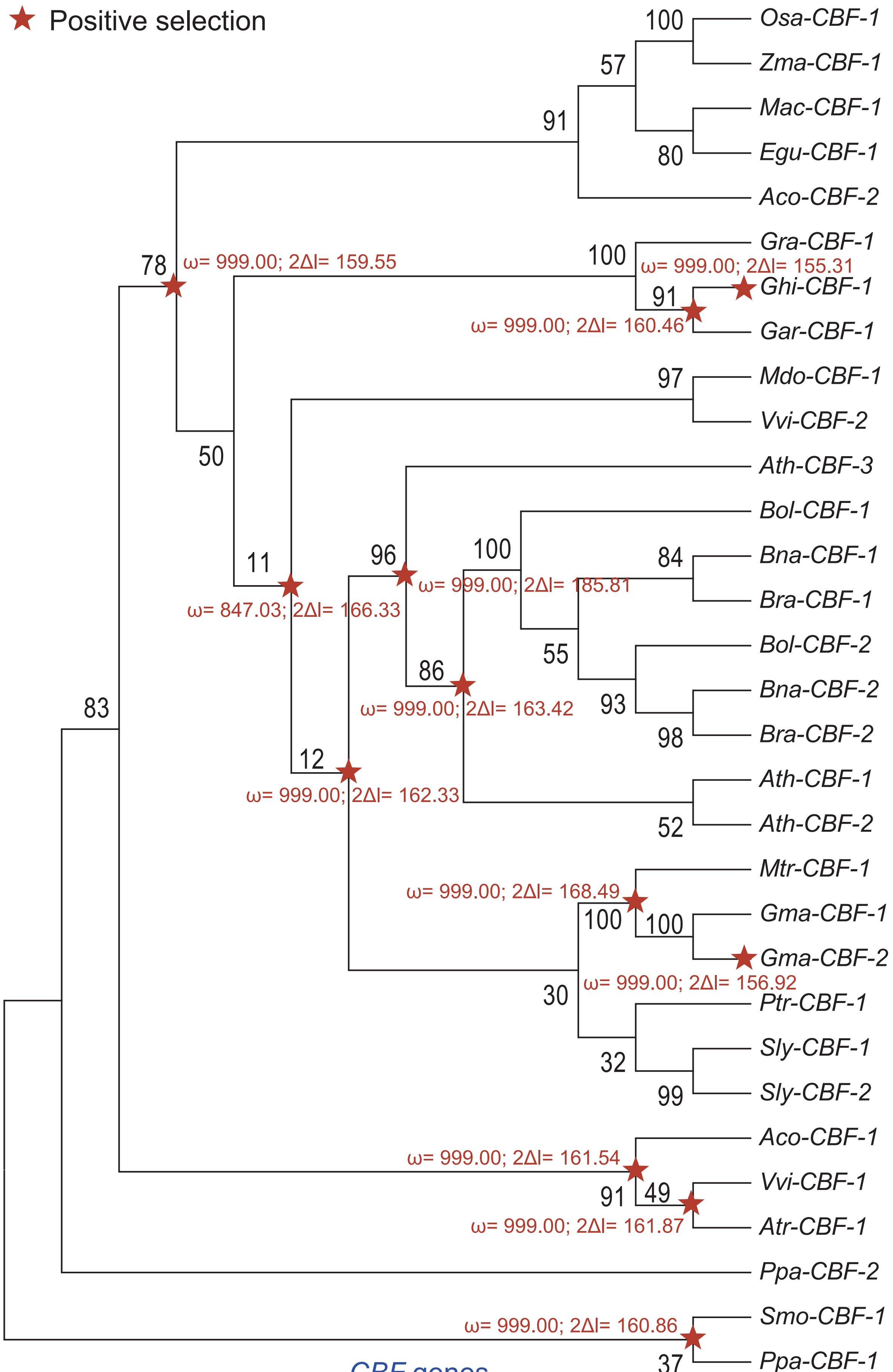

Positive selection
Osa-CBF-1
Zma-CBF-1
Mac-CBF-1
Egu-CBF-1
Aco-CBF-2
Gra-CBF-1
Ghi-CBF-1
Gar-CBF-1
Mdo-CBF-1
Vvi-CBF-2
Ath-CBF-3
Bol-CBF-1
Bna-CBF-1
Bra-CBF-1
Bol-CBF-2
Bna-CBF-2
Bra-CBF-2
Ath-CBF-1
Ath-CBF-2
Mtr-CBF-1
Gma-CBF-1
Gma-CBF-2
Ptr-CBF-1
Sly-CBF-1
Sly-CBF-2
Aco-CBF-1
Vvi-CBF-1
Atr-CBF-1
Ppa-CBF-2
Smo-CBF-1
Ppa-CBF-1
ω= 999.00; 2Δl= 159.55
ω= 999.00; 2Δl= 155.31
ω= 999.00; 2Δl= 160.46
ω= 847.03; 2Δl= 166.33
ω= 999.00; 2Δl= 185.81
ω= 999.00; 2Δl= 163.42
ω= 999.00; 2Δl= 162.33
ω= 999.00; 2Δl= 168.49
ω= 999.00; 2Δl= 156.92
ω= 999.00; 2Δl= 161.54
ω= 999.00; 2Δl= 161.87
ω= 999.00; 2Δl= 160.86
100
57
91
80
78
100
91
97
50
11
96
100
84
55
86
93
98
83
12
52
100
100
30
32
99
91
49
37

CBF genes

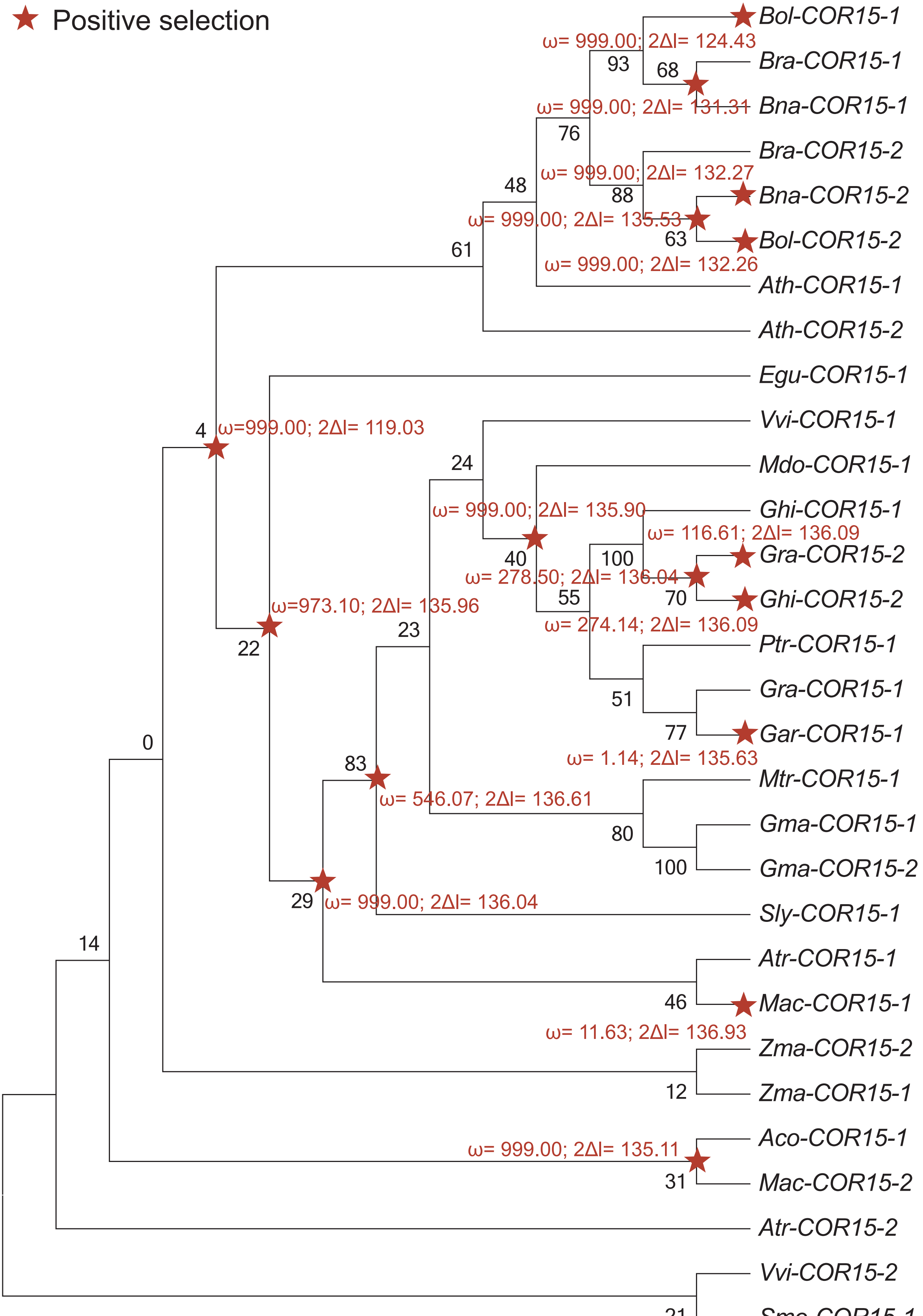

Positive selection
Bol-COR15-1
Bra-COR15-1
Bna-COR15-1
Bra-COR15-2
Bna-COR15-2
Bol-COR15-2
Ath-COR15-1
Ath-COR15-2
Egu-COR15-1
Vvi-COR15-1
Mdo-COR15-1
Ghi-COR15-1
Gra-COR15-2
Ghi-COR15-2
Ptr-COR15-1
Gra-COR15-1
Gar-COR15-1
Mtr-COR15-1
Gma-COR15-1
Gma-COR15-2
Sly-COR15-1
Atr-COR15-1
Mac-COR15-1
Zma-COR15-2
Zma-COR15-1
Aco-COR15-1
Mac-COR15-2
Atr-COR15-2
Vvi-COR15-2
Smo-COR15-1
ω= 999.00; 2ΔI= 124.43
ω= 999.00; 2ΔI= 131.31
ω= 999.00; 2ΔI= 132.27
ω= 999.00; 2ΔI= 135.53
ω= 999.00; 2ΔI= 132.26
ω=999.00; 2ΔI= 119.03
ω= 999.00; 2ΔI= 135.90
ω= 116.61; 2ΔI= 136.09
ω= 278.50; 2ΔI= 136.04
ω=973.10; 2ΔI= 135.96
ω= 274.14; 2ΔI= 136.09
ω= 1.14; 2ΔI= 135.63
ω= 546.07; 2ΔI= 136.61
ω= 999.00; 2ΔI= 136.04
ω= 11.63; 2ΔI= 136.93
ω= 999.00; 2ΔI= 135.11
93
68
76
48
88
63
61
4
24
40
100
55
70
22
23
51
77
0
83
80
100
29
14
46
12
31
21

COR15 genes

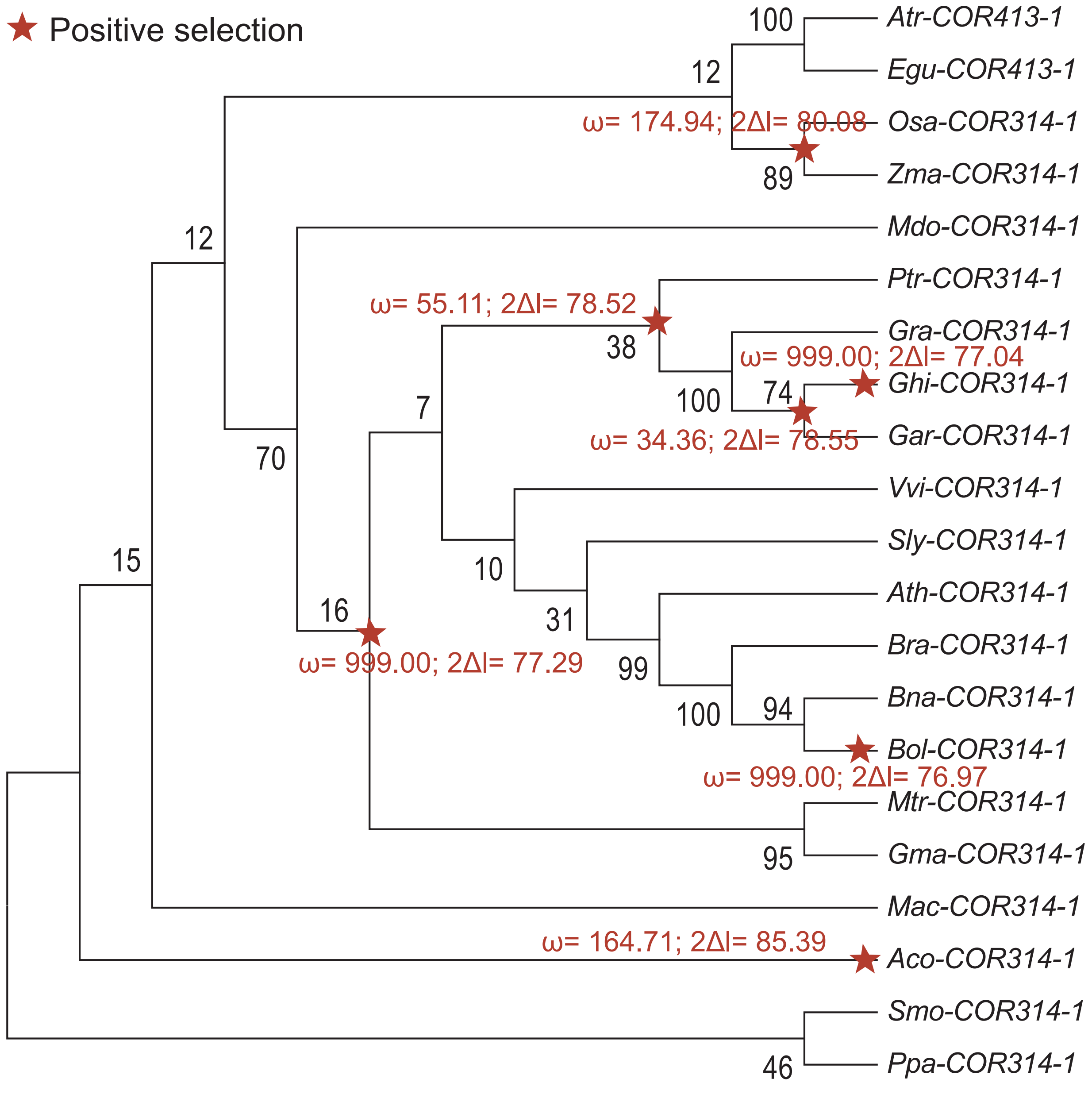

COR314 genes

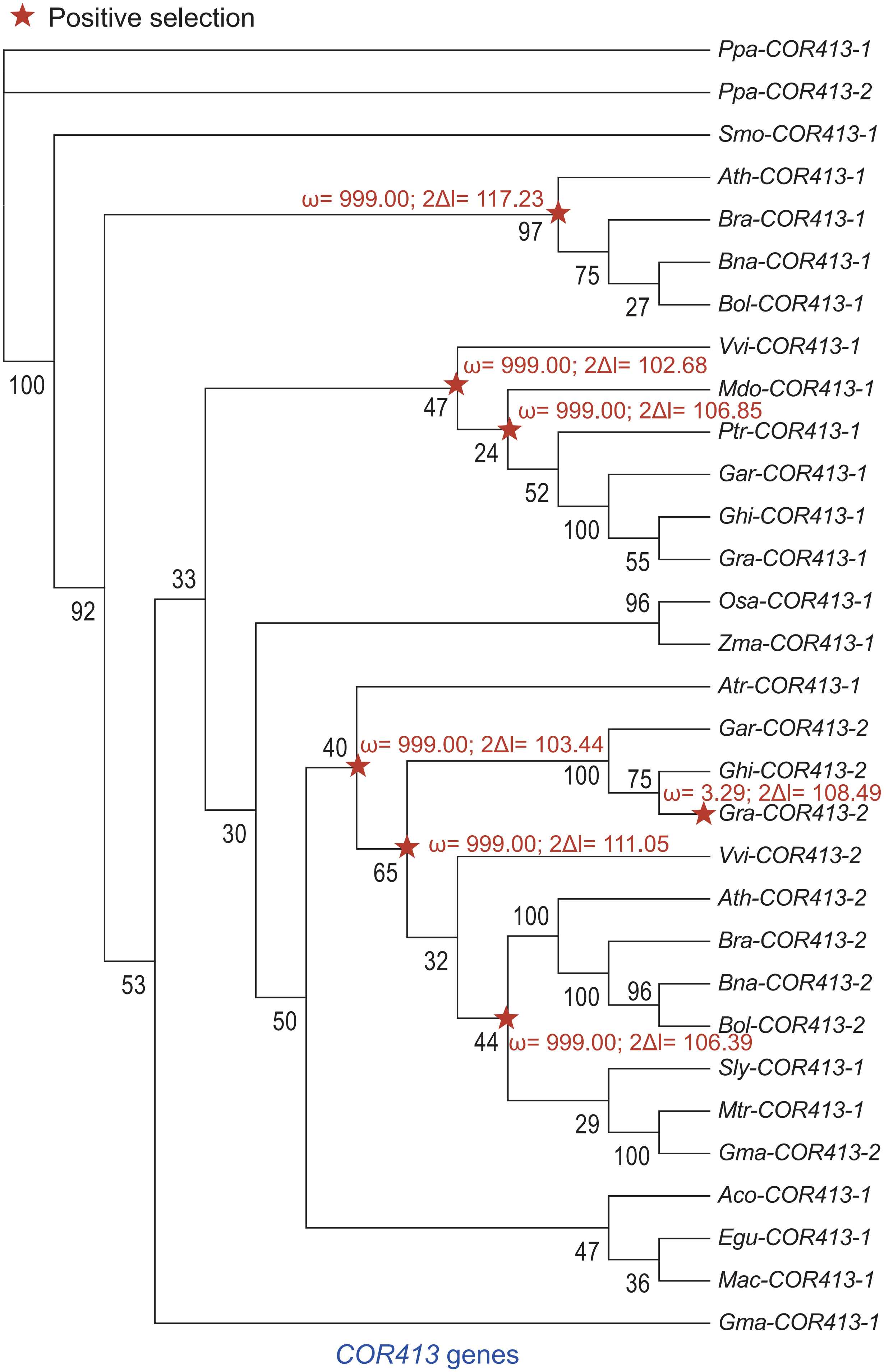
Positive selection
Ppa-COR413-1
Ppa-COR413-2
Smo-COR413-1
Ath-COR413-1
Bra-COR413-1
Bna-COR413-1
Bol-COR413-1
Vvi-COR413-1
Mdo-COR413-1
Ptr-COR413-1
Gar-COR413-1
Ghi-COR413-1
Gra-COR413-1
Osa-COR413-1
Zma-COR413-1
Atr-COR413-1
Gar-COR413-2
Ghi-COR413-2
Gra-COR413-2
Vvi-COR413-2
Ath-COR413-2
Bra-COR413-2
Bna-COR413-2
Bol-COR413-2
Sly-COR413-1
Mtr-COR413-1
Gma-COR413-2
Aco-COR413-1
Egu-COR413-1
Mac-COR413-1
Gma-COR413-1
ω= 999.00; 2Δl= 117.23
ω= 999.00; 2Δl= 102.68
ω= 999.00; 2Δl= 106.85
ω= 999.00; 2Δl= 103.44
ω= 3.29; 2Δl= 108.49
ω= 999.00; 2Δl= 111.05
ω= 999.00; 2Δl= 106.39
COR413 genes

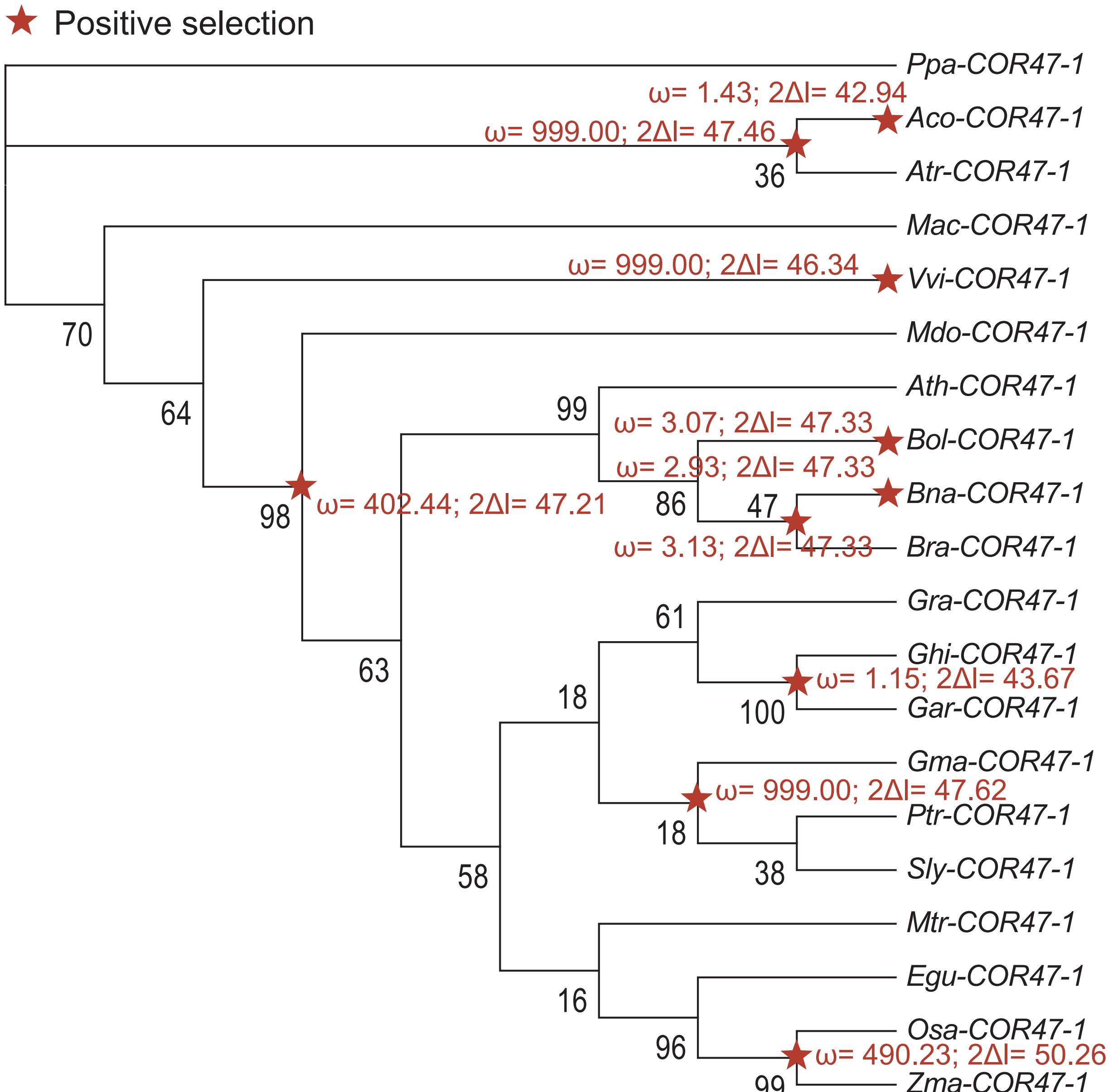
Positive selection
Ppa-COR47-1
ω= 1.43; 2Δl= 42.94
Aco-COR47-1
ω= 999.00; 2Δl= 47.46
36
Atr-COR47-1
Mac-COR47-1
ω= 999.00; 2Δl= 46.34
Vvi-COR47-1
70
Mdo-COR47-1
Ath-COR47-1
64
99
ω= 3.07; 2Δl= 47.33
Bol-COR47-1
ω= 2.93; 2Δl= 47.33
Bna-COR47-1
98
ω= 402.44; 2Δl= 47.21
86
47
ω= 3.13; 2Δl= 47.33
Bra-COR47-1
61
Gra-COR47-1
Ghi-COR47-1
ω= 1.15; 2Δl= 43.67
63
18
100
Gar-COR47-1
Gma-COR47-1
ω= 999.00; 2Δl= 47.62
18
Ptr-COR47-1
58
38
Sly-COR47-1
Mtr-COR47-1
Egu-COR47-1
16
96
Osa-COR47-1
ω= 490.23; 2Δl= 50.26
99
Zma-COR47-1

COR47 genes

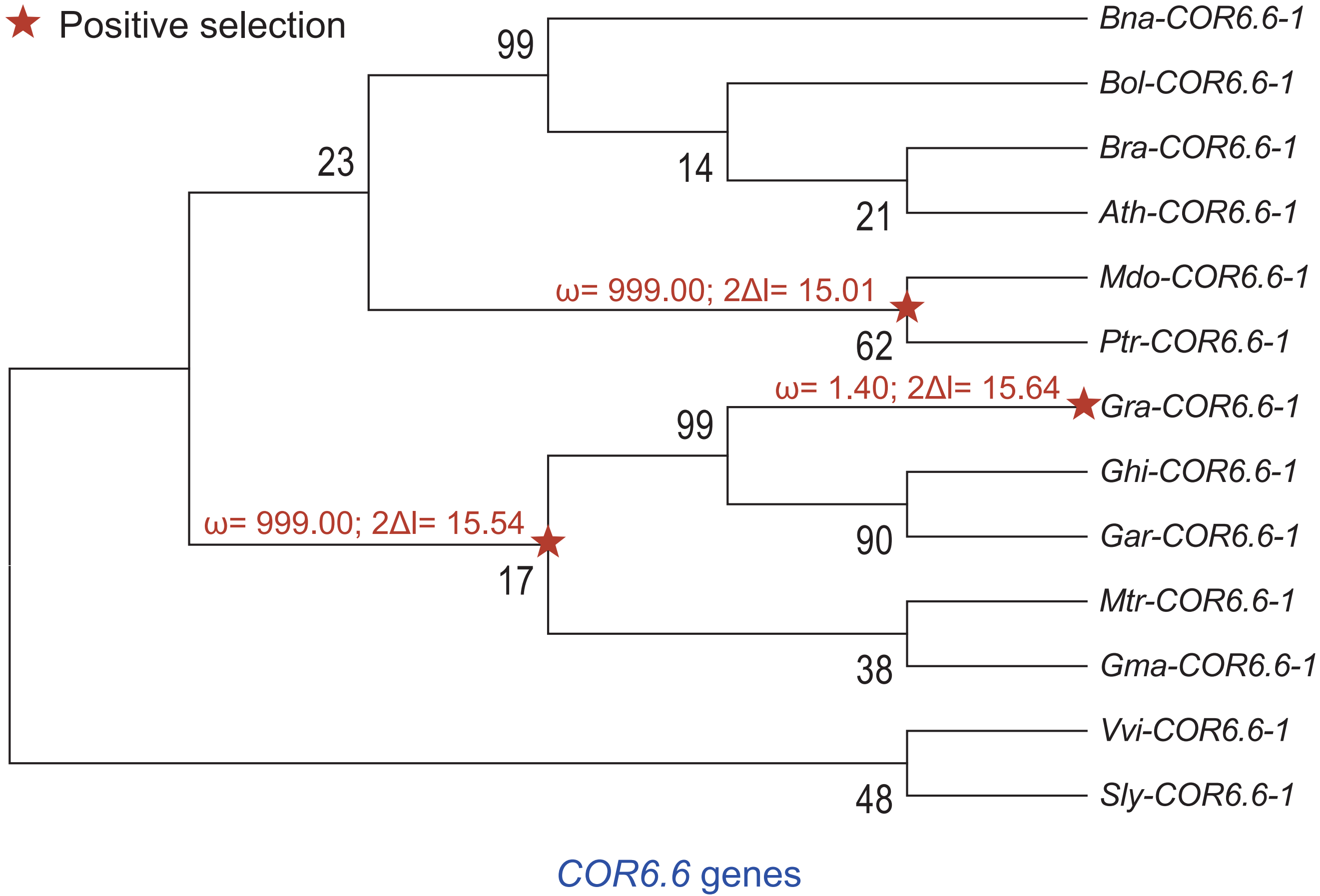

Positive selection
99
Bna-COR6.6-1
Bol-COR6.6-1
23
14
Bra-COR6.6-1
21
Ath-COR6.6-1
ω= 999.00; 2Δl= 15.01
Mdo-COR6.6-1
62
Ptr-COR6.6-1
ω= 1.40; 2Δl= 15.64
Gra-COR6.6-1
99
Ghi-COR6.6-1
ω= 999.00; 2Δl= 15.54
90
Gar-COR6.6-1
17
Mtr-COR6.6-1
38
Gma-COR6.6-1
Vvi-COR6.6-1
48
Sly-COR6.6-1
COR6.6 genes

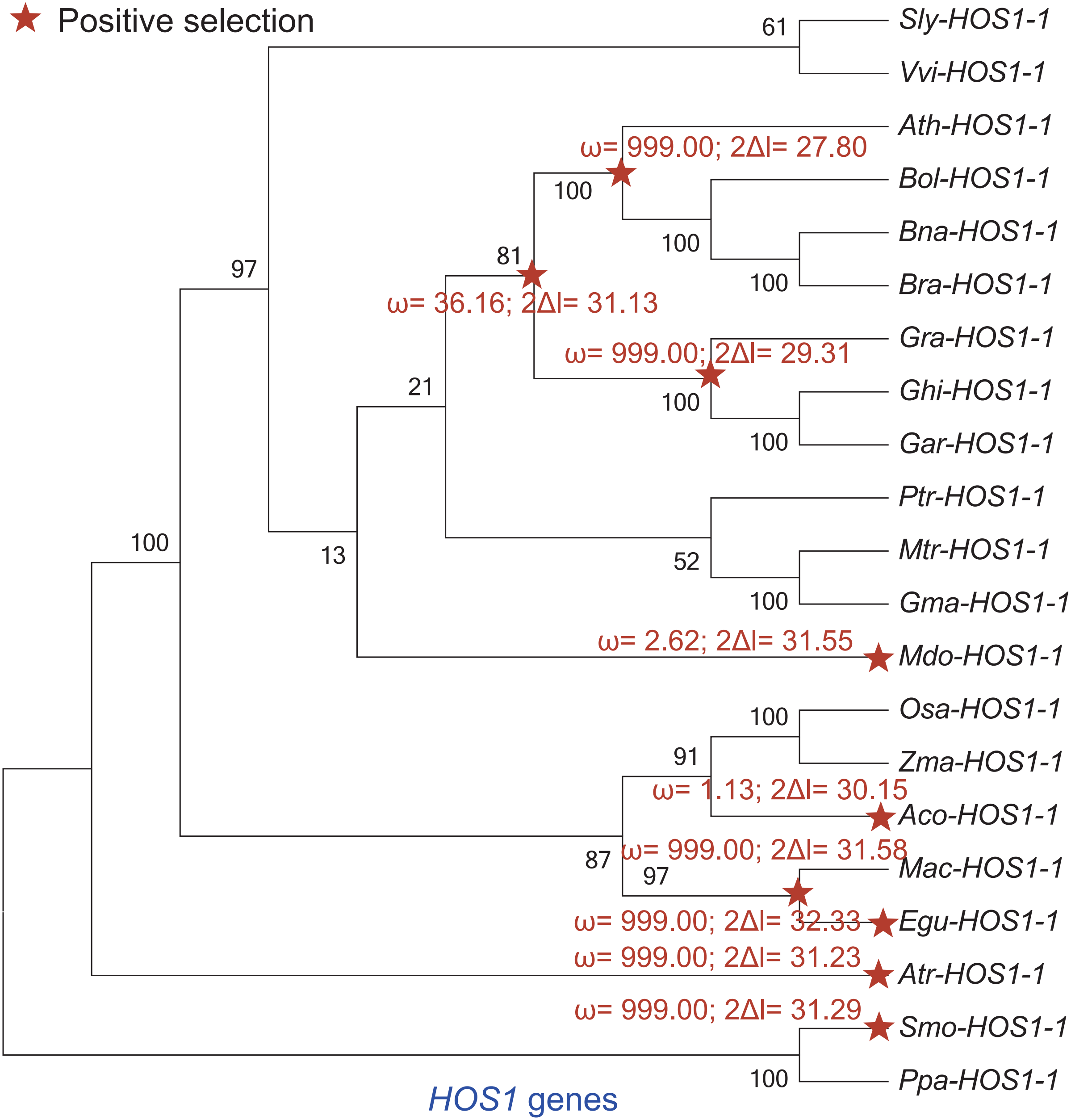

Positive selection
Sly-HOS1-1
Vvi-HOS1-1
Ath-HOS1-1
Bol-HOS1-1
Bna-HOS1-1
Bra-HOS1-1
Gra-HOS1-1
Ghi-HOS1-1
Gar-HOS1-1
Ptr-HOS1-1
Mtr-HOS1-1
Gma-HOS1-1
Mdo-HOS1-1
Osa-HOS1-1
Zma-HOS1-1
Aco-HOS1-1
Mac-HOS1-1
Egu-HOS1-1
Atr-HOS1-1
Smo-HOS1-1
Ppa-HOS1-1
ω= 999.00; 2Δl= 27.80
ω= 36.16; 2Δl= 31.13
ω= 999.00; 2Δl= 29.31
ω= 2.62; 2Δl= 31.55
ω= 1.13; 2Δl= 30.15
ω= 999.00; 2Δl= 31.58
ω= 999.00; 2Δl= 32.33
ω= 999.00; 2Δl= 31.23
ω= 999.00; 2Δl= 31.29
61
100
100
100
81
97
100
100
21
100
13
52
100
100
91
87
97
100
HOS1 genes

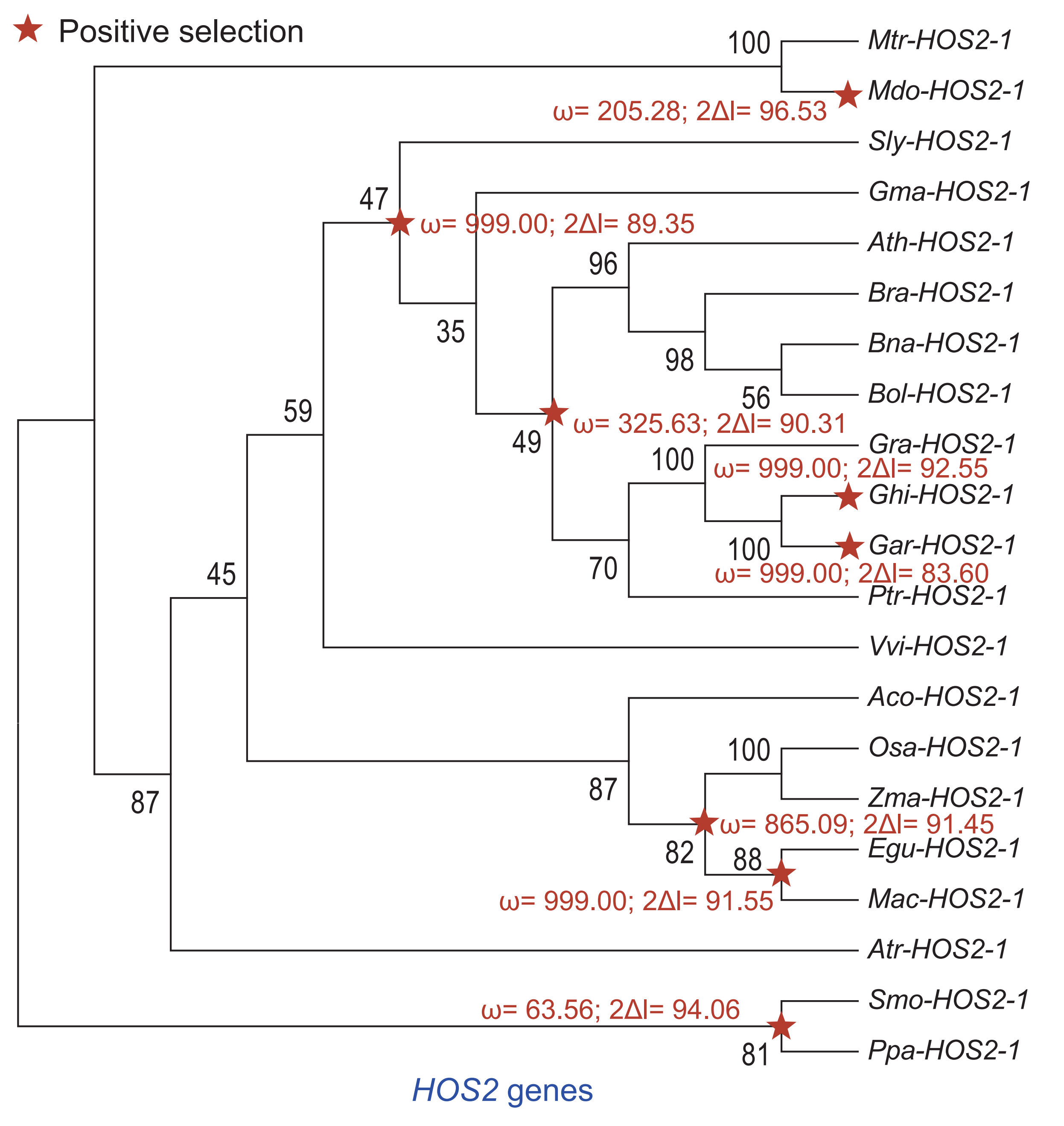

Positive selection
100
Mtr-HOS2-1
Mdo-HOS2-1
ω= 205.28; 2Δl= 96.53
Sly-HOS2-1
47
Gma-HOS2-1
ω= 999.00; 2Δl= 89.35
Ath-HOS2-1
96
Bra-HOS2-1
35
Bna-HOS2-1
98
59
Bol-HOS2-1
56
ω= 325.63; 2Δl= 90.31
49
Gra-HOS2-1
100
ω= 999.00; 2Δl= 92.55
Ghi-HOS2-1
Gar-HOS2-1
100
ω= 999.00; 2Δl= 83.60
45
70
Ptr-HOS2-1
Vvi-HOS2-1
Aco-HOS2-1
100
Osa-HOS2-1
Zma-HOS2-1
87
87
ω= 865.09; 2Δl= 91.45
82
88
Egu-HOS2-1
Mac-HOS2-1
ω= 999.00; 2Δl= 91.55
Atr-HOS2-1
Smo-HOS2-1
ω= 63.56; 2Δl= 94.06
81
Ppa-HOS2-1
HOS2 genes

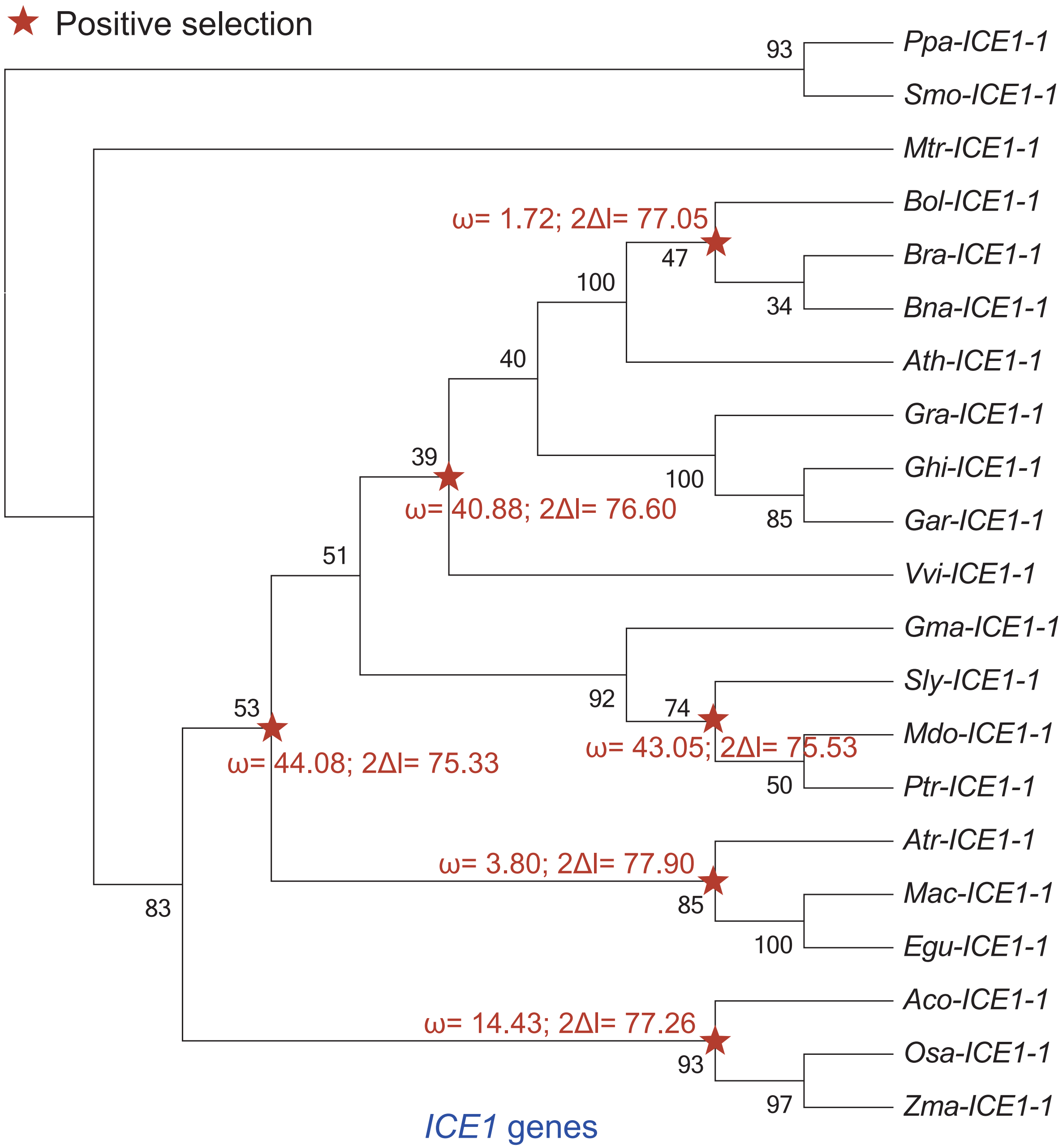
Positive selection
Ppa-ICE1-1
Smo-ICE1-1
Mtr-ICE1-1
Bol-ICE1-1
Bra-ICE1-1
Bna-ICE1-1
Ath-ICE1-1
Gra-ICE1-1
Ghi-ICE1-1
Gar-ICE1-1
Vvi-ICE1-1
Gma-ICE1-1
Sly-ICE1-1
Mdo-ICE1-1
Ptr-ICE1-1
Atr-ICE1-1
Mac-ICE1-1
Egu-ICE1-1
Aco-ICE1-1
Osa-ICE1-1
Zma-ICE1-1
ω= 1.72; 2Δl= 77.05
ω= 40.88; 2Δl= 76.60
ω= 43.05; 2Δl= 75.53
ω= 44.08; 2Δl= 75.33
ω= 3.80; 2Δl= 77.90
ω= 14.43; 2Δl= 77.26
93
47
34
100
40
39
100
85
51
92
74
50
53
85
100
83
93
97
ICE1 genes

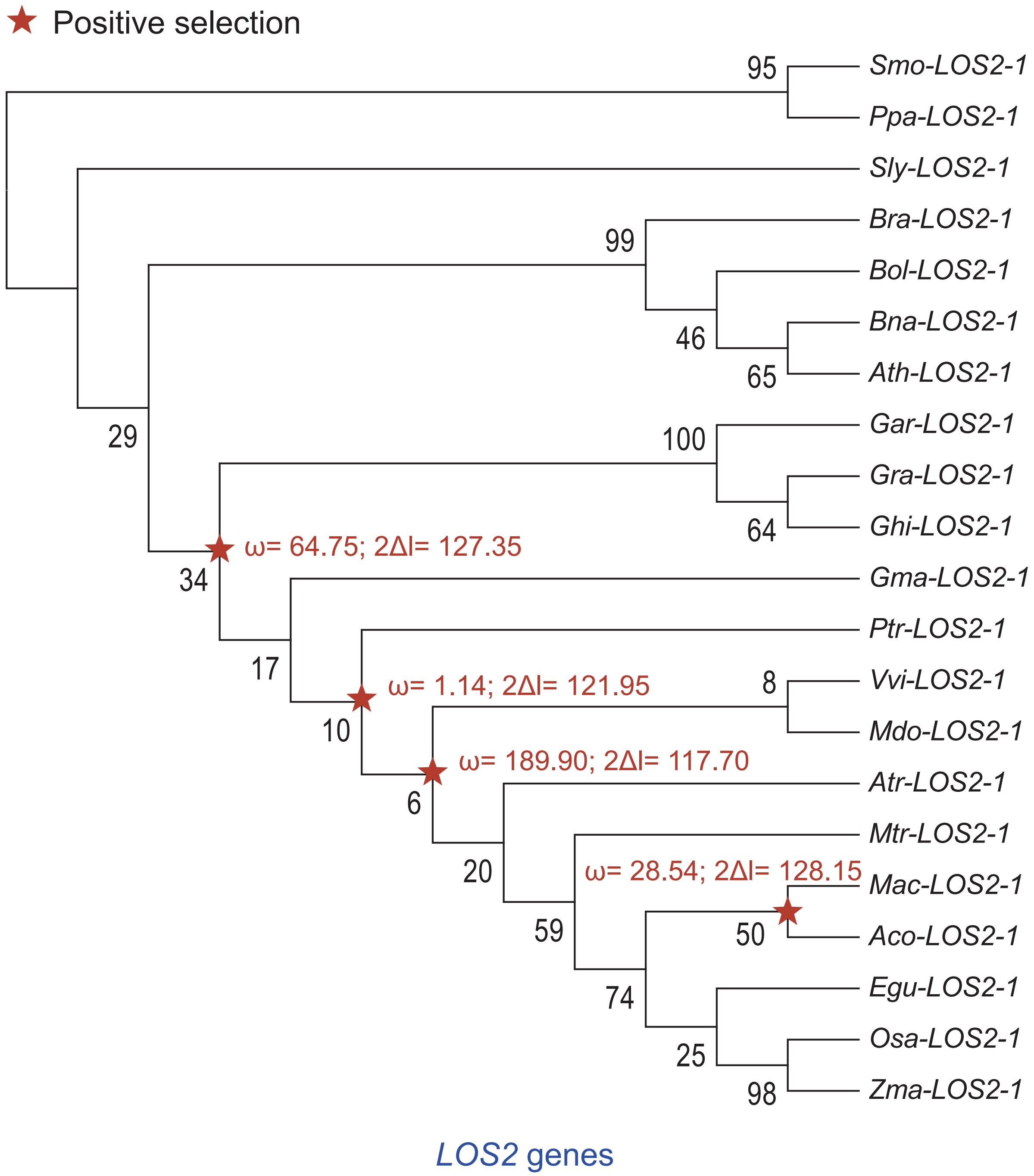
Positive selection
95
Smo-LOS2-1
Ppa-LOS2-1
Sly-LOS2-1
Bra-LOS2-1
99
Bol-LOS2-1
Bna-LOS2-1
46
65
Ath-LOS2-1
29
Gar-LOS2-1
100
Gra-LOS2-1
Ghi-LOS2-1
64
ω= 64.75; 2Δl= 127.35
34
Gma-LOS2-1
Ptr-LOS2-1
17
ω= 1.14; 2Δl= 121.95
8
Vvi-LOS2-1
10
Mdo-LOS2-1
ω= 189.90; 2Δl= 117.70
Atr-LOS2-1
6
Mtr-LOS2-1
20
ω= 28.54; 2Δl= 128.15
Mac-LOS2-1
59
50
Aco-LOS2-1
Egu-LOS2-1
74
Osa-LOS2-1
25
Zma-LOS2-1
98
LOS2 genes

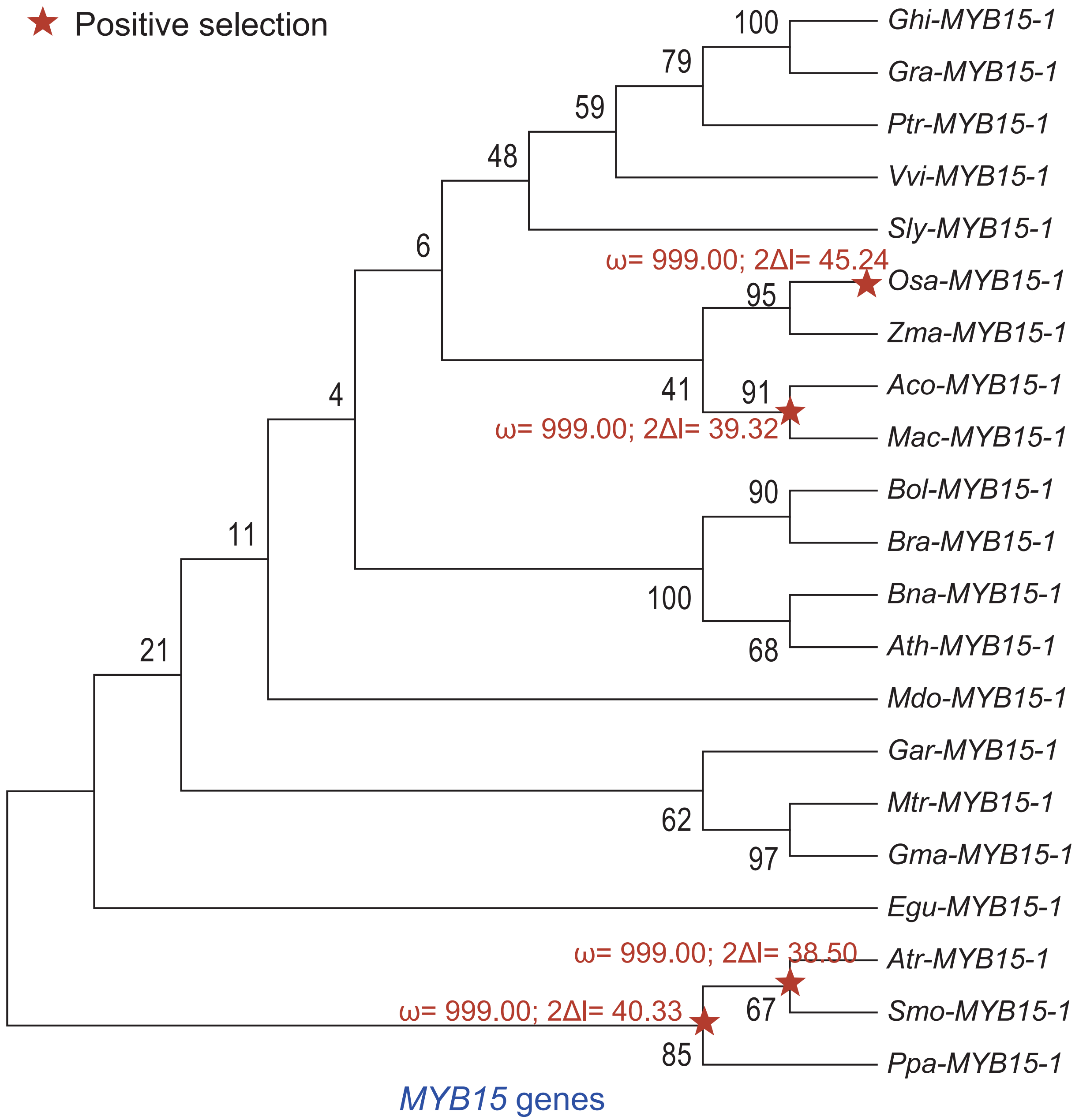
Positive selection
Ghi-MYB15-1
Gra-MYB15-1
Ptr-MYB15-1
Vvi-MYB15-1
Sly-MYB15-1
ω= 999.00; 2Δl= 45.24
Osa-MYB15-1
Zma-MYB15-1
Aco-MYB15-1
ω= 999.00; 2Δl= 39.32
Mac-MYB15-1
Bol-MYB15-1
Bra-MYB15-1
Bna-MYB15-1
Ath-MYB15-1
Mdo-MYB15-1
Gar-MYB15-1
Mtr-MYB15-1
Gma-MYB15-1
Egu-MYB15-1
ω= 999.00; 2Δl= 38.50
Atr-MYB15-1
ω= 999.00; 2Δl= 40.33
Smo-MYB15-1
Ppa-MYB15-1
100
79
59
48
6
95
41
91
4
90
11
100
68
21
62
97
67
85
MYB15 genes

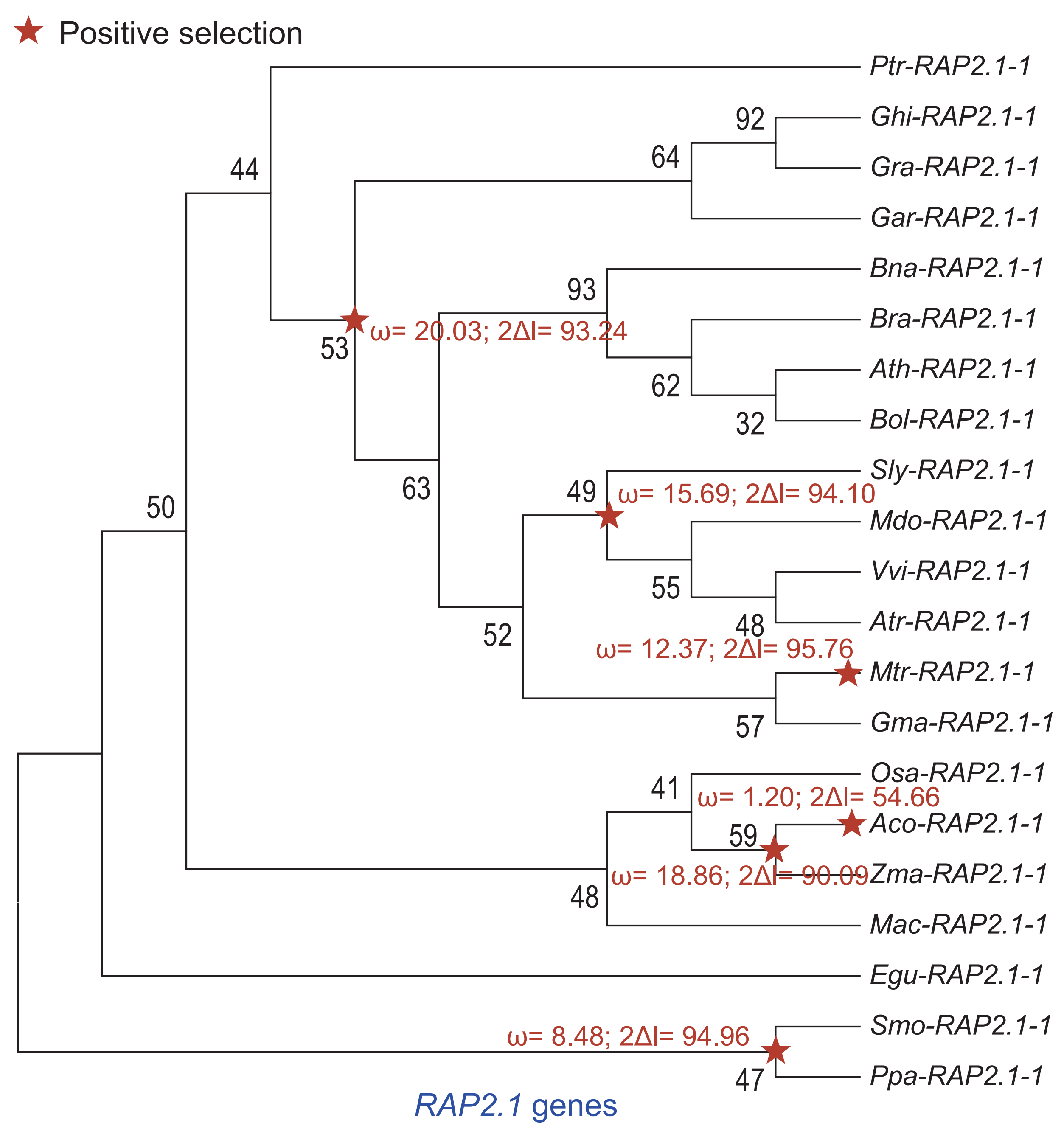

Positive selection
Ptr-RAP2.1-1
Ghi-RAP2.1-1
Gra-RAP2.1-1
Gar-RAP2.1-1
Bna-RAP2.1-1
Bra-RAP2.1-1
Ath-RAP2.1-1
Bol-RAP2.1-1
Sly-RAP2.1-1
Mdo-RAP2.1-1
Vvi-RAP2.1-1
Atr-RAP2.1-1
Mtr-RAP2.1-1
Gma-RAP2.1-1
Osa-RAP2.1-1
Aco-RAP2.1-1
Zma-RAP2.1-1
Mac-RAP2.1-1
Egu-RAP2.1-1
Smo-RAP2.1-1
Ppa-RAP2.1-1
ω= 20.03; 2ΔI= 93.24
ω= 15.69; 2ΔI= 94.10
ω= 12.37; 2ΔI= 95.76
ω= 1.20; 2ΔI= 54.66
ω= 18.86; 2ΔI= 90.09
ω= 8.48; 2ΔI= 94.96
44
92
64
93
53
62
32
63
50
49
55
48
52
57
41
59
48
47
RAP2.1 genes

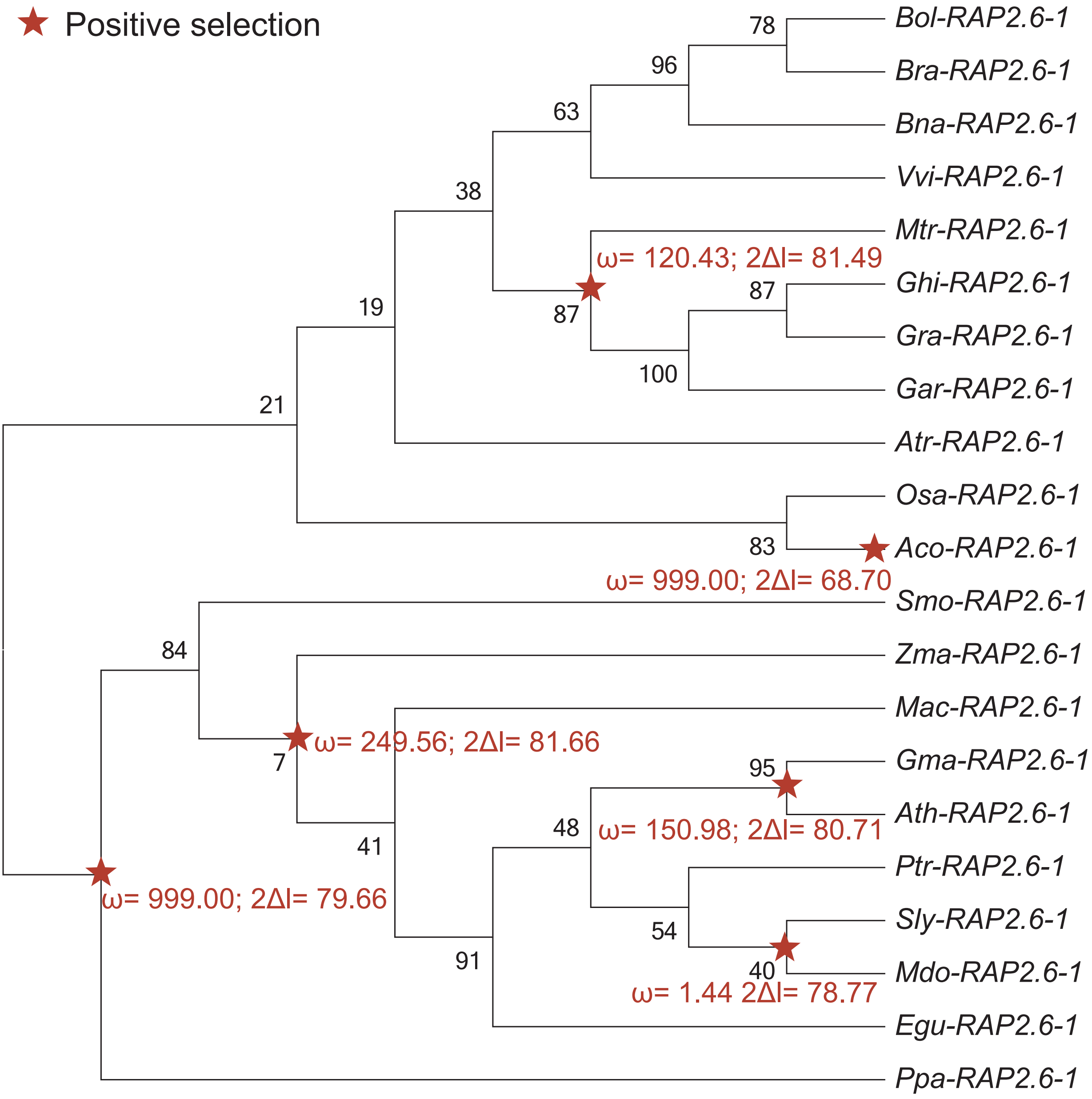
Positive selection
Bol-RAP2.6-1
Bra-RAP2.6-1
Bna-RAP2.6-1
Vvi-RAP2.6-1
Mtr-RAP2.6-1
Ghi-RAP2.6-1
Gra-RAP2.6-1
Gar-RAP2.6-1
Atr-RAP2.6-1
Osa-RAP2.6-1
Aco-RAP2.6-1
Smo-RAP2.6-1
Zma-RAP2.6-1
Mac-RAP2.6-1
Gma-RAP2.6-1
Ath-RAP2.6-1
Ptr-RAP2.6-1
Sly-RAP2.6-1
Mdo-RAP2.6-1
Egu-RAP2.6-1
Ppa-RAP2.6-1
78
96
63
38
19
21
87
87
100
83
84
7
41
95
48
54
40
91
ω= 120.43; 2Δl= 81.49
ω= 999.00; 2Δl= 68.70
ω= 249.56; 2Δl= 81.66
ω= 150.98; 2Δl= 80.71
ω= 999.00; 2Δl= 79.66
ω= 1.44 2Δl= 78.77
RAP2.6 genes

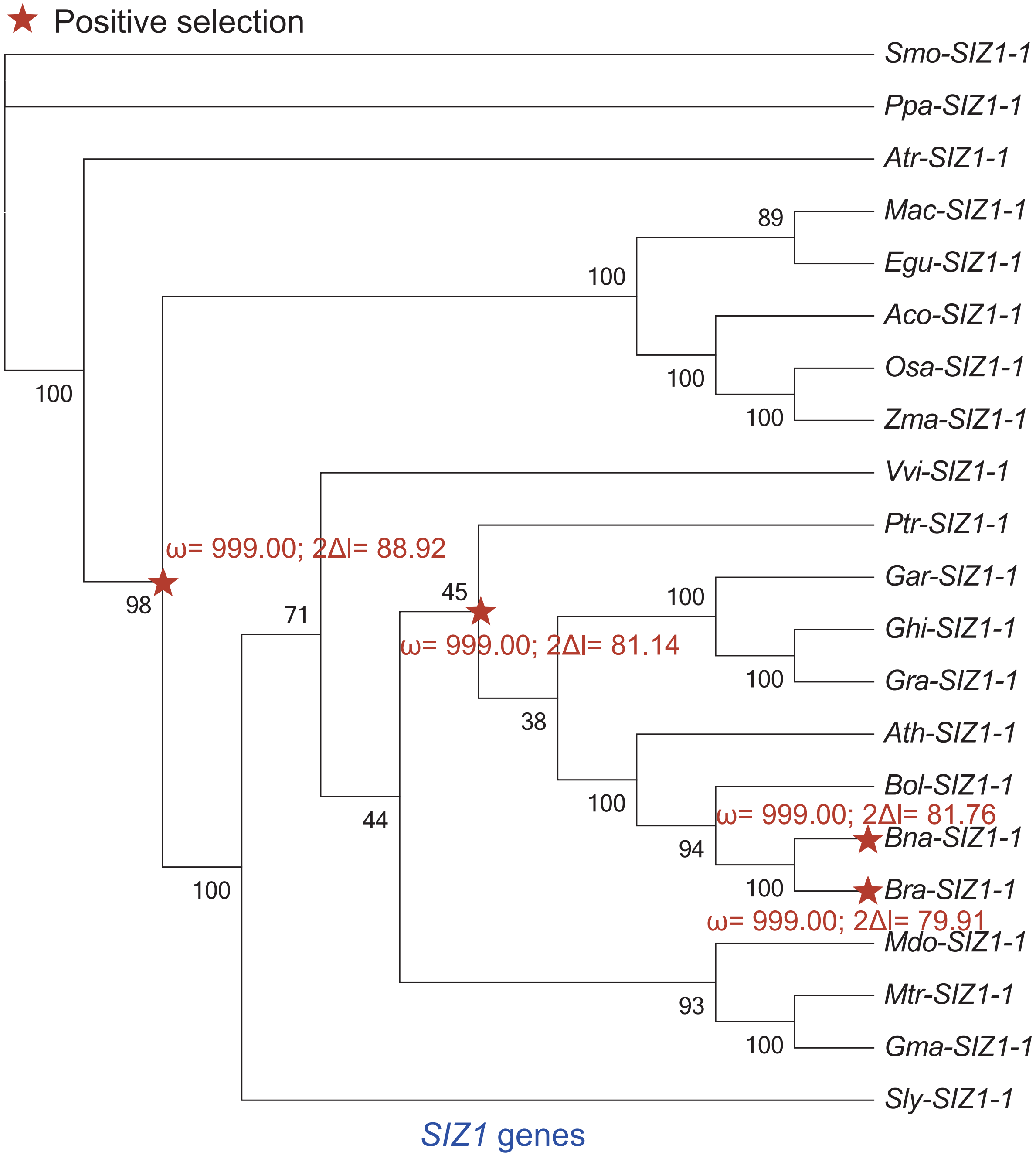

★ Positive selection
Smo-SIZ1-1
Ppa-SIZ1-1
Atr-SIZ1-1
Mac-SIZ1-1
Egu-SIZ1-1
Aco-SIZ1-1
Osa-SIZ1-1
Zma-SIZ1-1
Vvi-SIZ1-1
Ptr-SIZ1-1
Gar-SIZ1-1
Ghi-SIZ1-1
Gra-SIZ1-1
Ath-SIZ1-1
Bol-SIZ1-1
Bna-SIZ1-1
Bra-SIZ1-1
Mdo-SIZ1-1
Mtr-SIZ1-1
Gma-SIZ1-1
Sly-SIZ1-1
ω= 999.00; 2Δl= 88.92
ω= 999.00; 2Δl= 81.14
ω= 999.00; 2Δl= 81.76
ω= 999.00; 2Δl= 79.91
89
100
100
100
100
98
71
45
100
100
38
100
94
100
44
100
93
100
SIZ1 genes

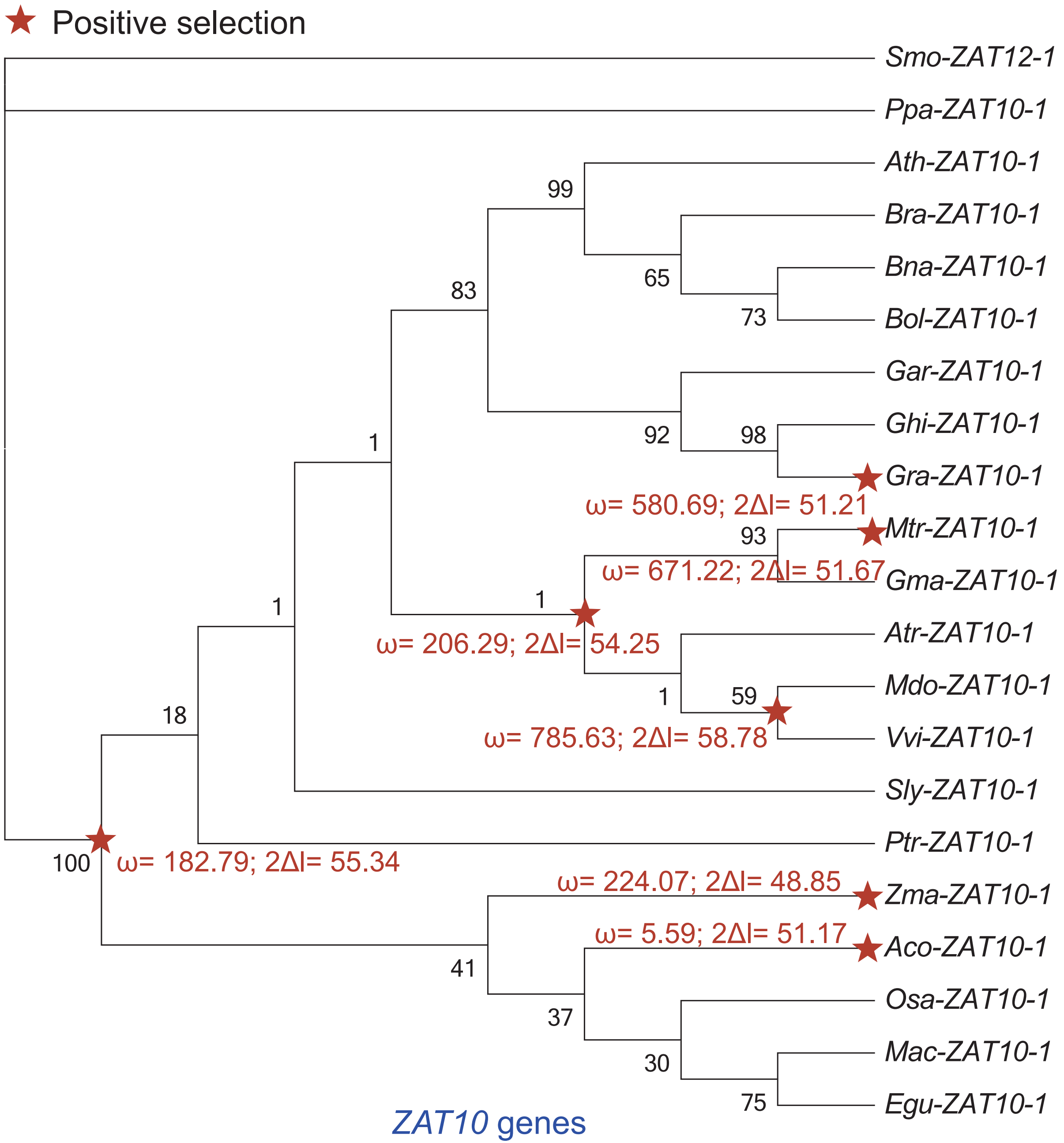
Positive selection
Smo-ZAT12-1
Ppa-ZAT10-1
Ath-ZAT10-1
Bra-ZAT10-1
Bna-ZAT10-1
Bol-ZAT10-1
Gar-ZAT10-1
Ghi-ZAT10-1
Gra-ZAT10-1
Mtr-ZAT10-1
Gma-ZAT10-1
Atr-ZAT10-1
Mdo-ZAT10-1
Vvi-ZAT10-1
Sly-ZAT10-1
Ptr-ZAT10-1
Zma-ZAT10-1
Aco-ZAT10-1
Osa-ZAT10-1
Mac-ZAT10-1
Egu-ZAT10-1
99
83
65
73
92
98
1
93
1
1
1
59
18
100
41
37
30
75
ω= 580.69; 2Δl= 51.21
ω= 671.22; 2Δl= 51.67
ω= 206.29; 2Δl= 54.25
ω= 785.63; 2Δl= 58.78
ω= 182.79; 2Δl= 55.34
ω= 224.07; 2Δl= 48.85
ω= 5.59; 2Δl= 51.17
ZAT10 genes

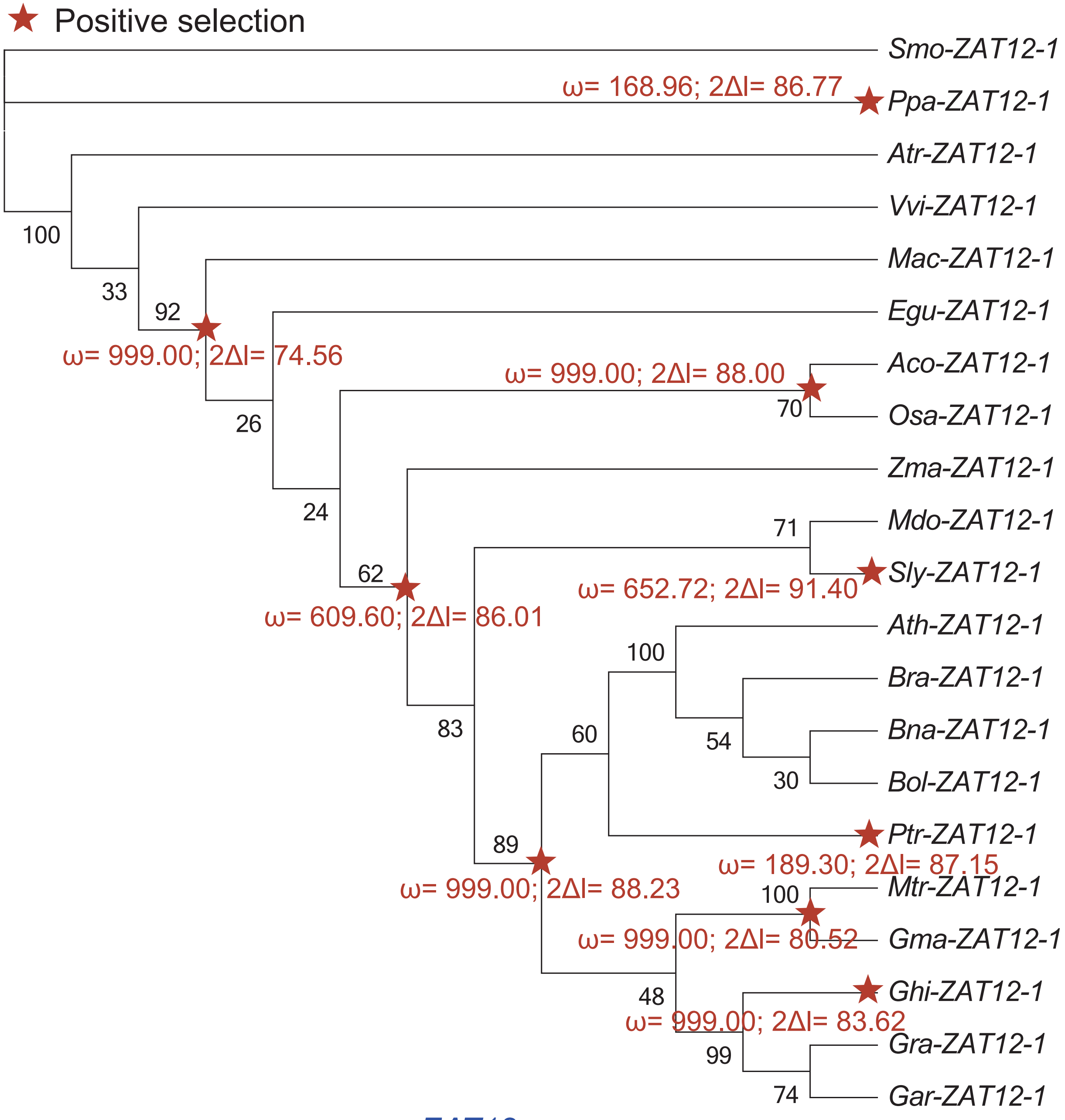
Positive selection
Smo-ZAT12-1
ω= 168.96; 2Δl= 86.77
Ppa-ZAT12-1
Atr-ZAT12-1
Vvi-ZAT12-1
Mac-ZAT12-1
Egu-ZAT12-1
ω= 999.00; 2Δl= 74.56
Aco-ZAT12-1
ω= 999.00; 2Δl= 88.00
Osa-ZAT12-1
Zma-ZAT12-1
Mdo-ZAT12-1
Sly-ZAT12-1
ω= 652.72; 2Δl= 91.40
ω= 609.60; 2Δl= 86.01
Ath-ZAT12-1
Bra-ZAT12-1
Bna-ZAT12-1
Bol-ZAT12-1
Ptr-ZAT12-1
ω= 189.30; 2Δl= 87.15
ω= 999.00; 2Δl= 88.23
Mtr-ZAT12-1
ω= 999.00; 2Δl= 80.52
Gma-ZAT12-1
Ghi-ZAT12-1
ω= 999.00; 2Δl= 83.62
Gra-ZAT12-1
Gar-ZAT12-1
100
33
92
26
70
24
62
71
100
83
60
54
30
89
100
48
99
74

ZAT12 genes

The large separated supplementary Figures

Figure S8. The Ks analyses for cold-related genes (*CRGs*) and *NBS* genes in each species.

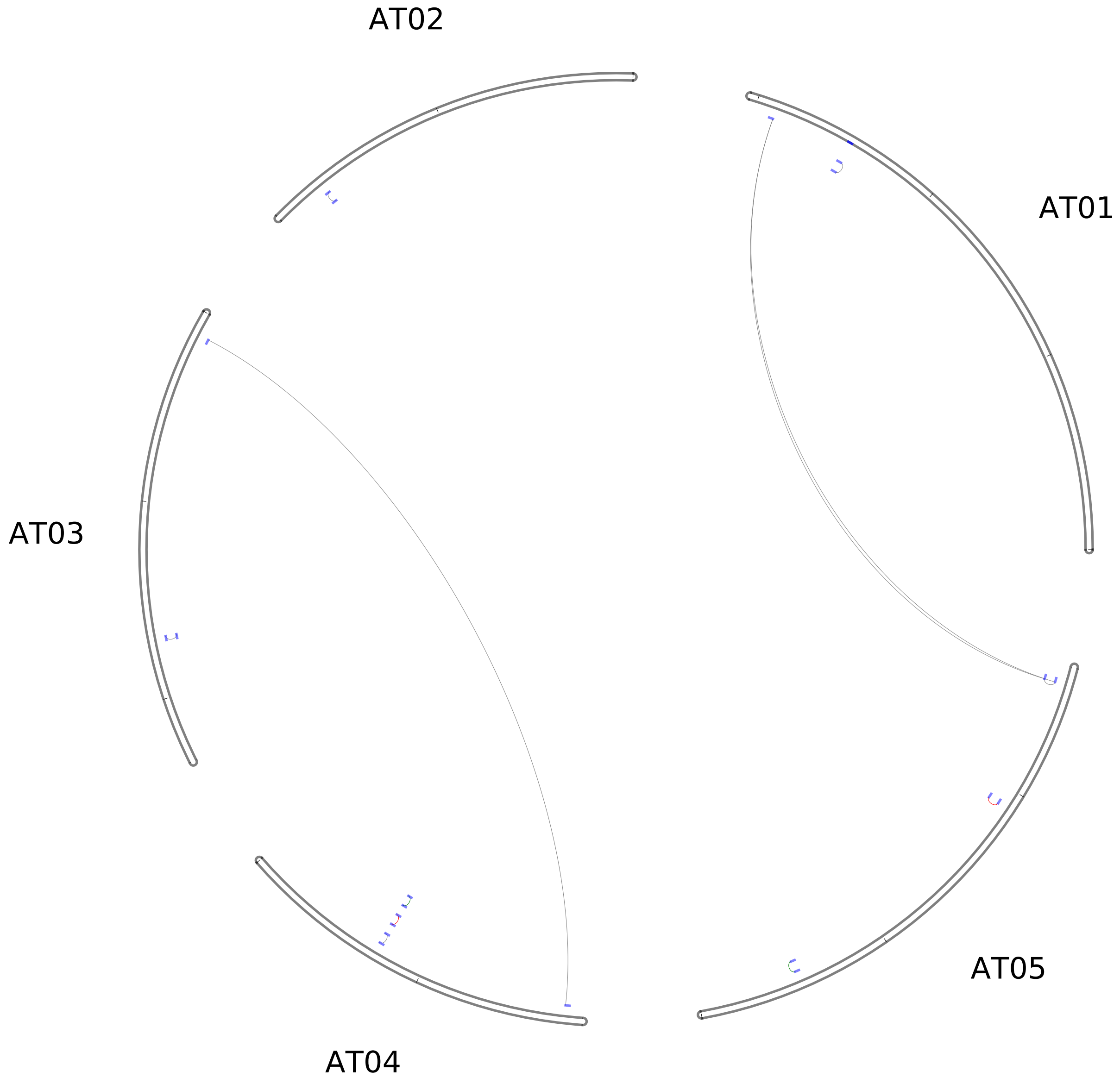
AT02
AT01
AT03
AT05
AT04

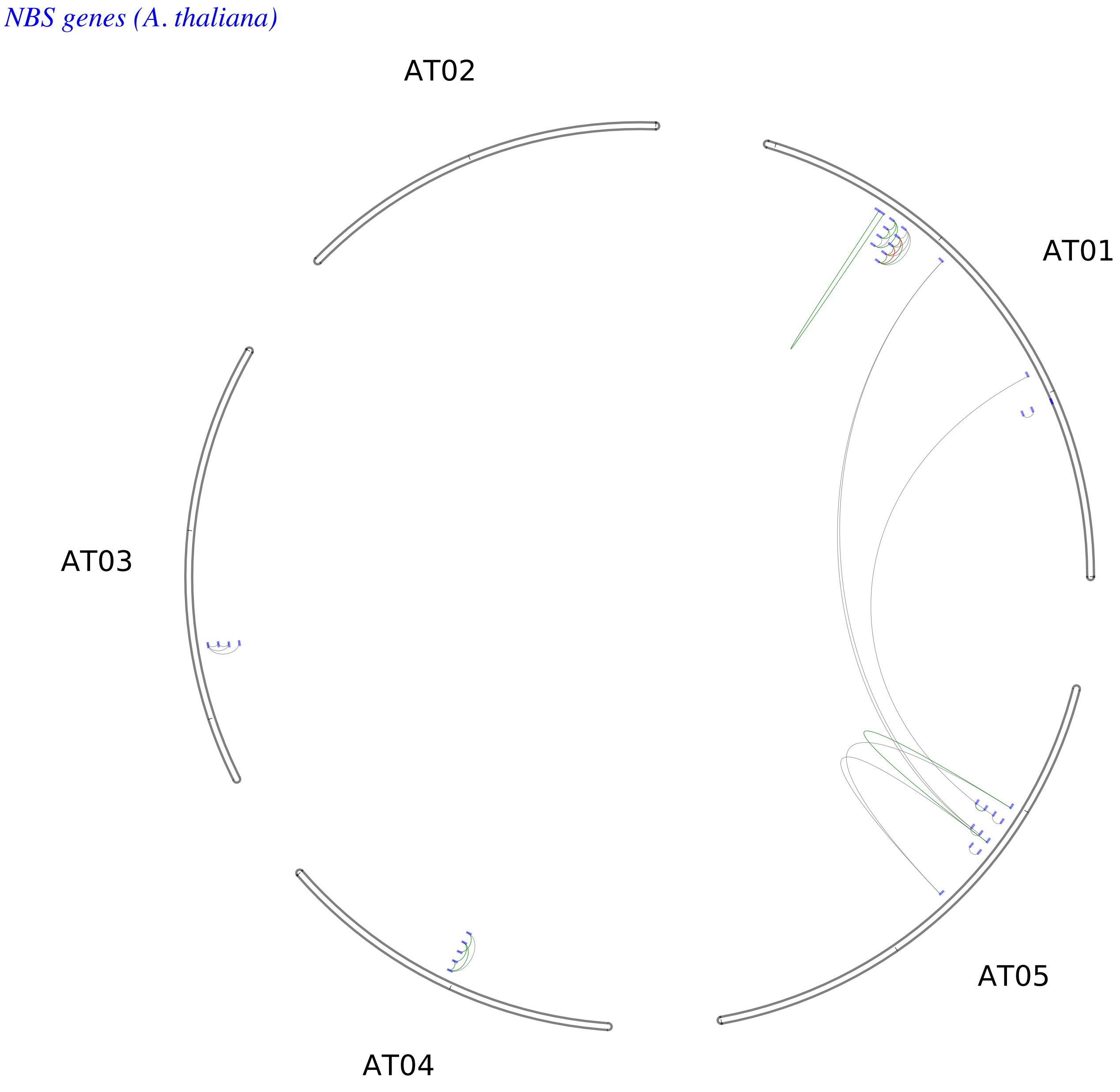
NBS genes (A. thaliana)
AT02
AT01
AT03
AT05
AT04

Cold genes (B. napus)

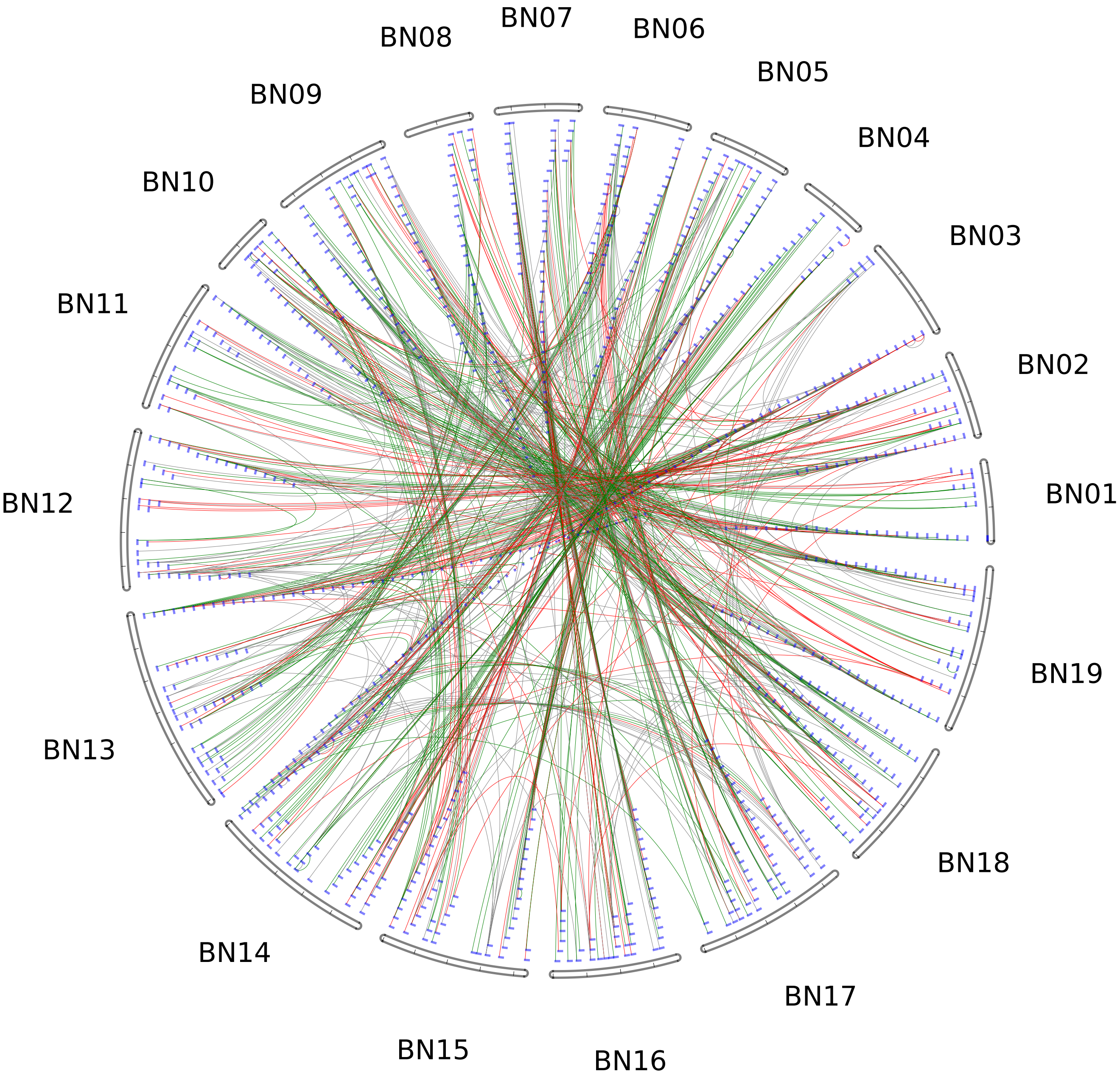

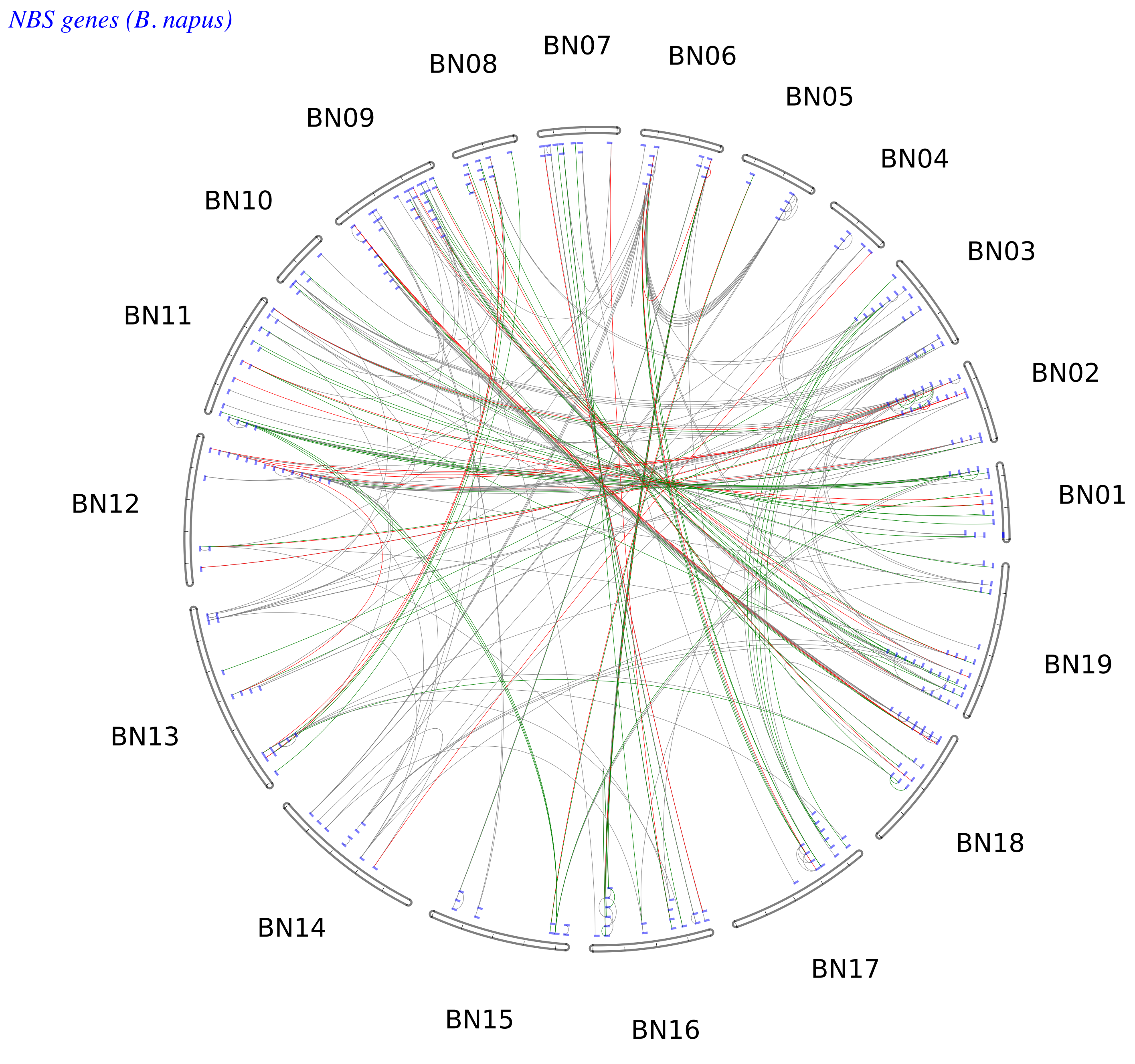
NBS genes (B. napus)
BN01
BN02
BN03
BN04
BN05
BN06
BN07
BN08
BN09
BN10
BN11
BN12
BN13
BN14
BN15
BN16
BN17
BN18
BN19

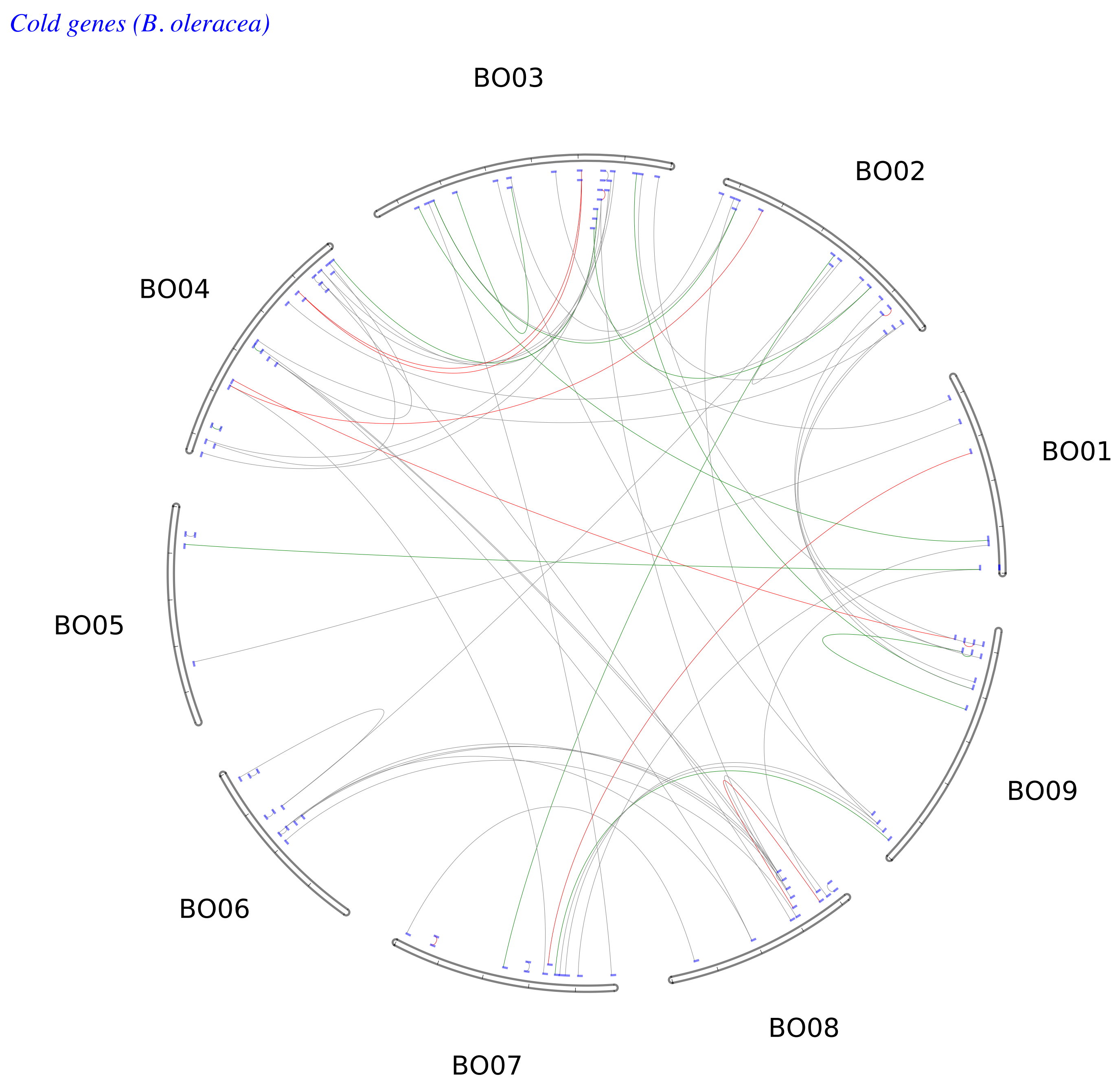
Cold genes (B. oleracea)
BO03
BO02
BO04
BO01
BO05
BO09
BO06
BO08
BO07

NBS genes (B. oleracea)

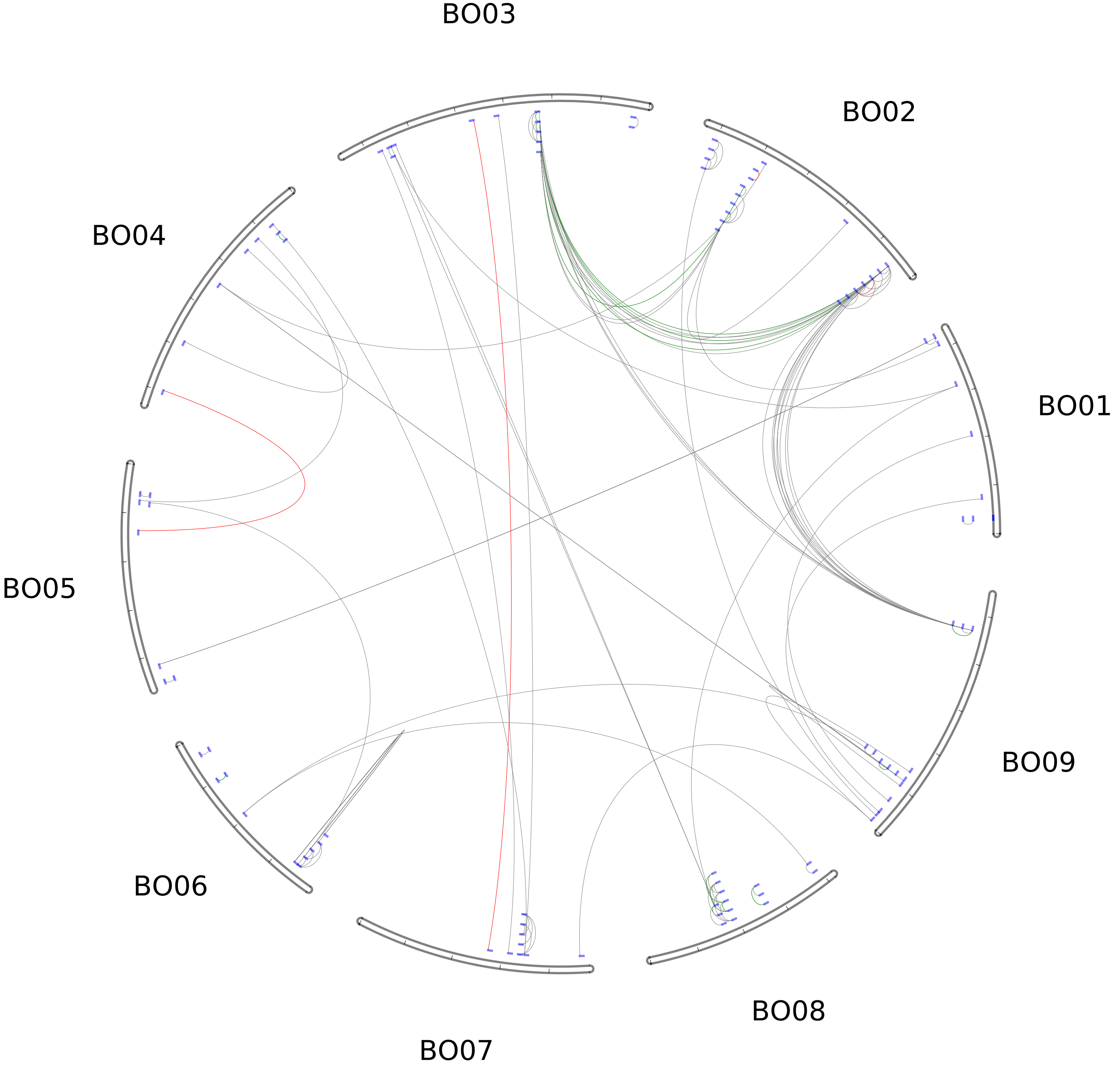

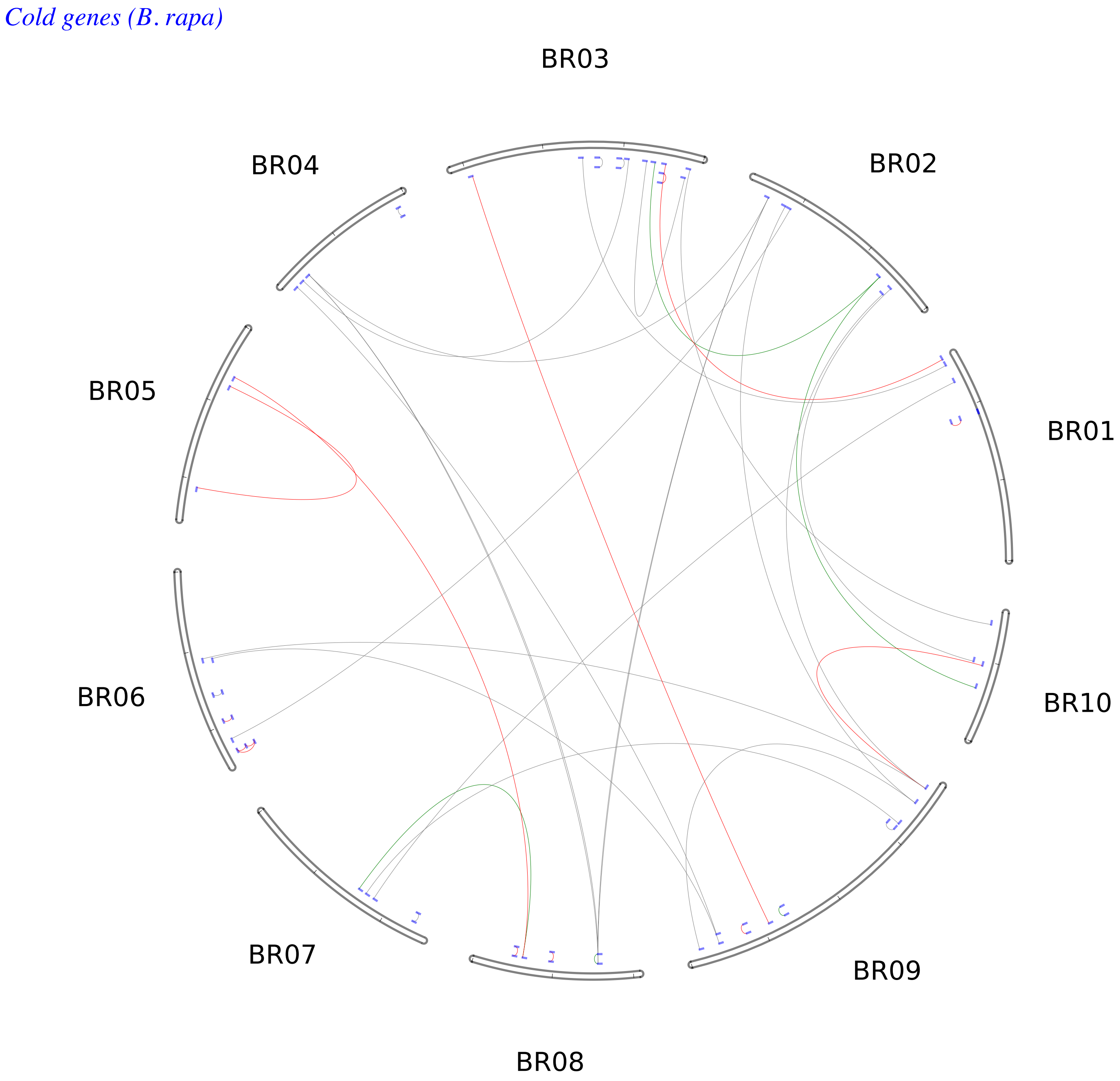
Cold genes (B. rapa)
BR03
BR04
BR02
BR05
BR01
BR06
BR10
BR07
BR09
BR08

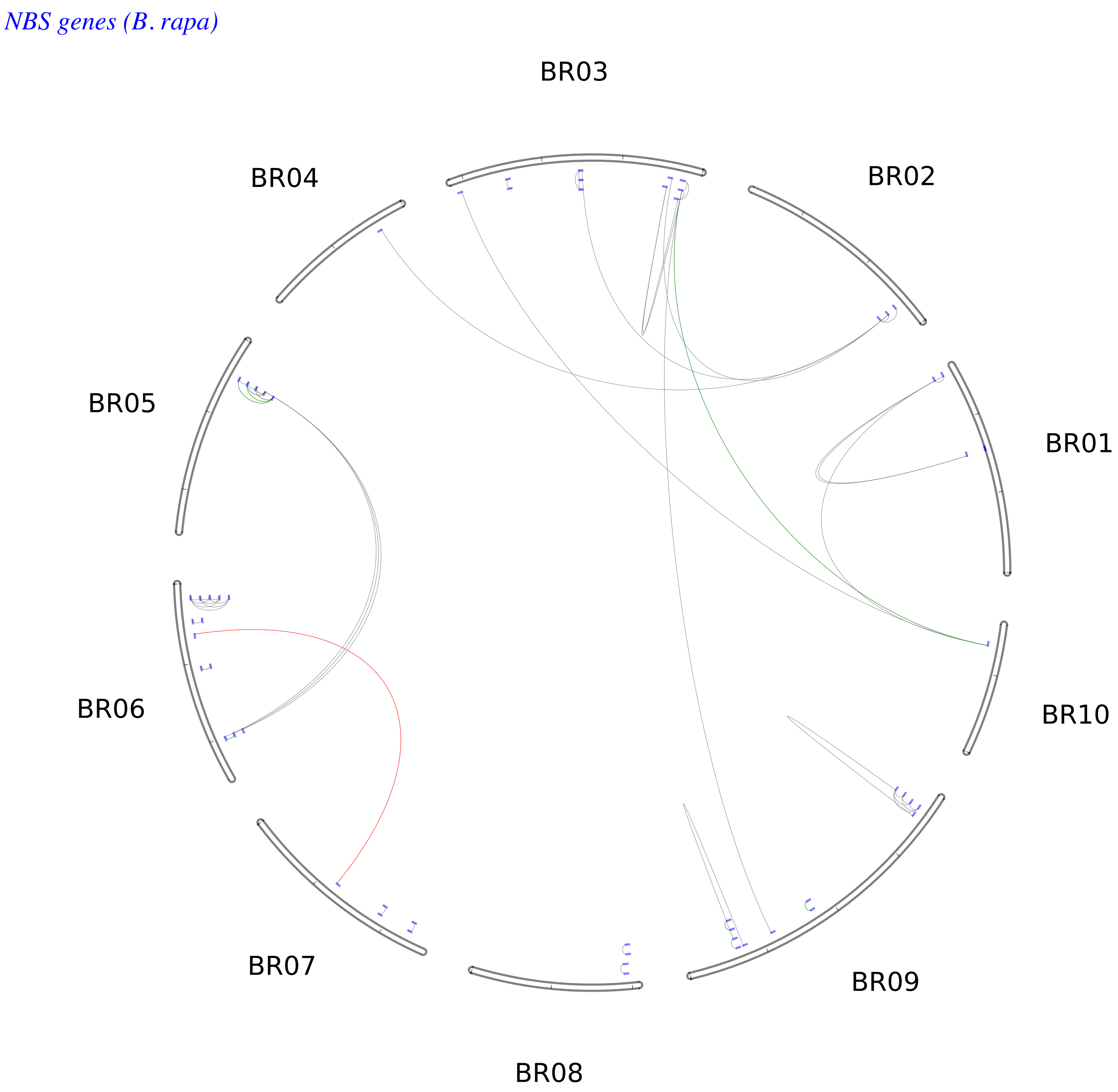
NBS genes (B. rapa)
BR03
BR04
BR02
BR05
BR01
BR06
BR10
BR07
BR09
BR08

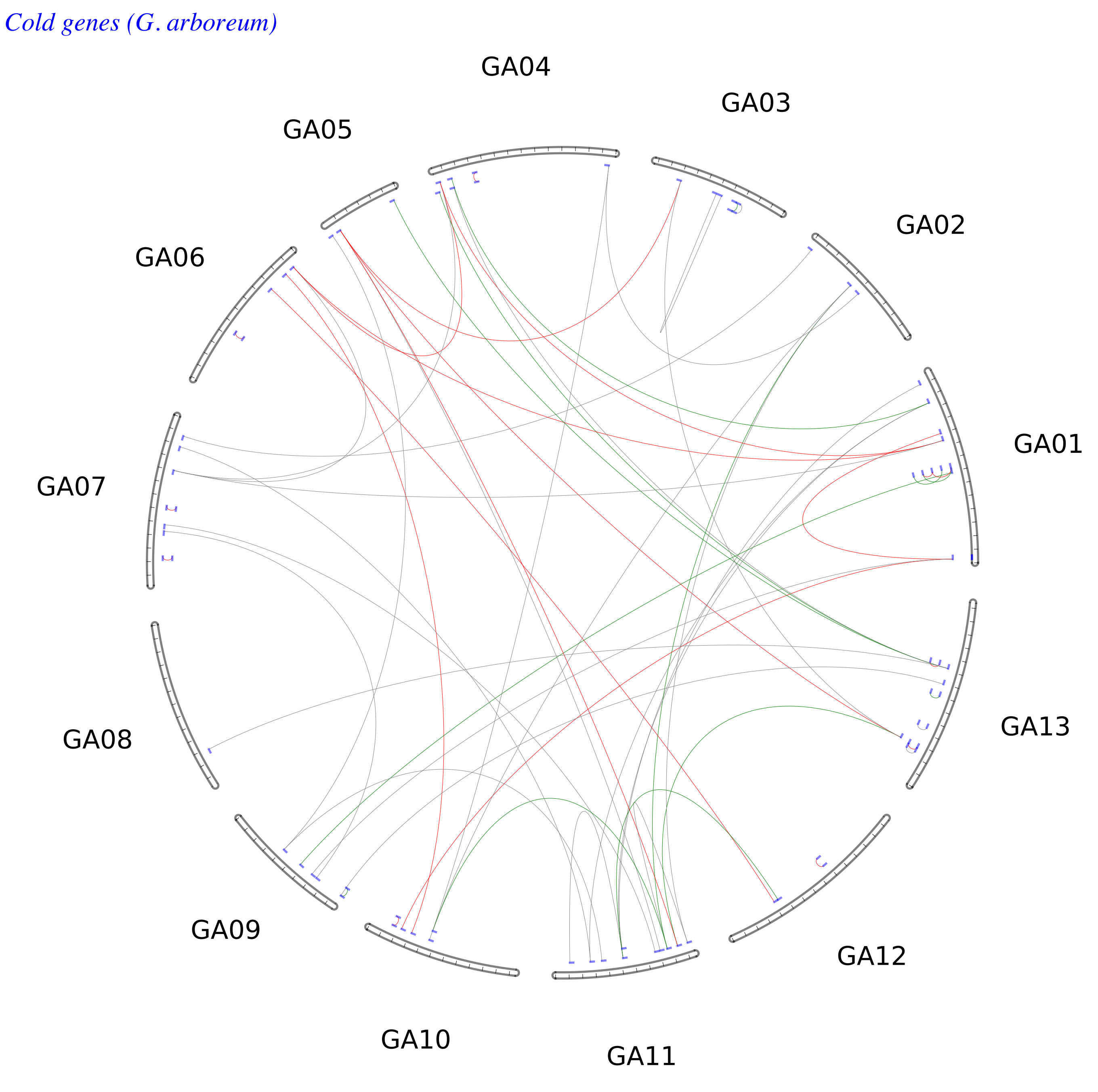

Cold genes (G. arboreum)
GA01
GA02
GA03
GA04
GA05
GA06
GA07
GA08
GA09
GA10
GA11
GA12
GA13

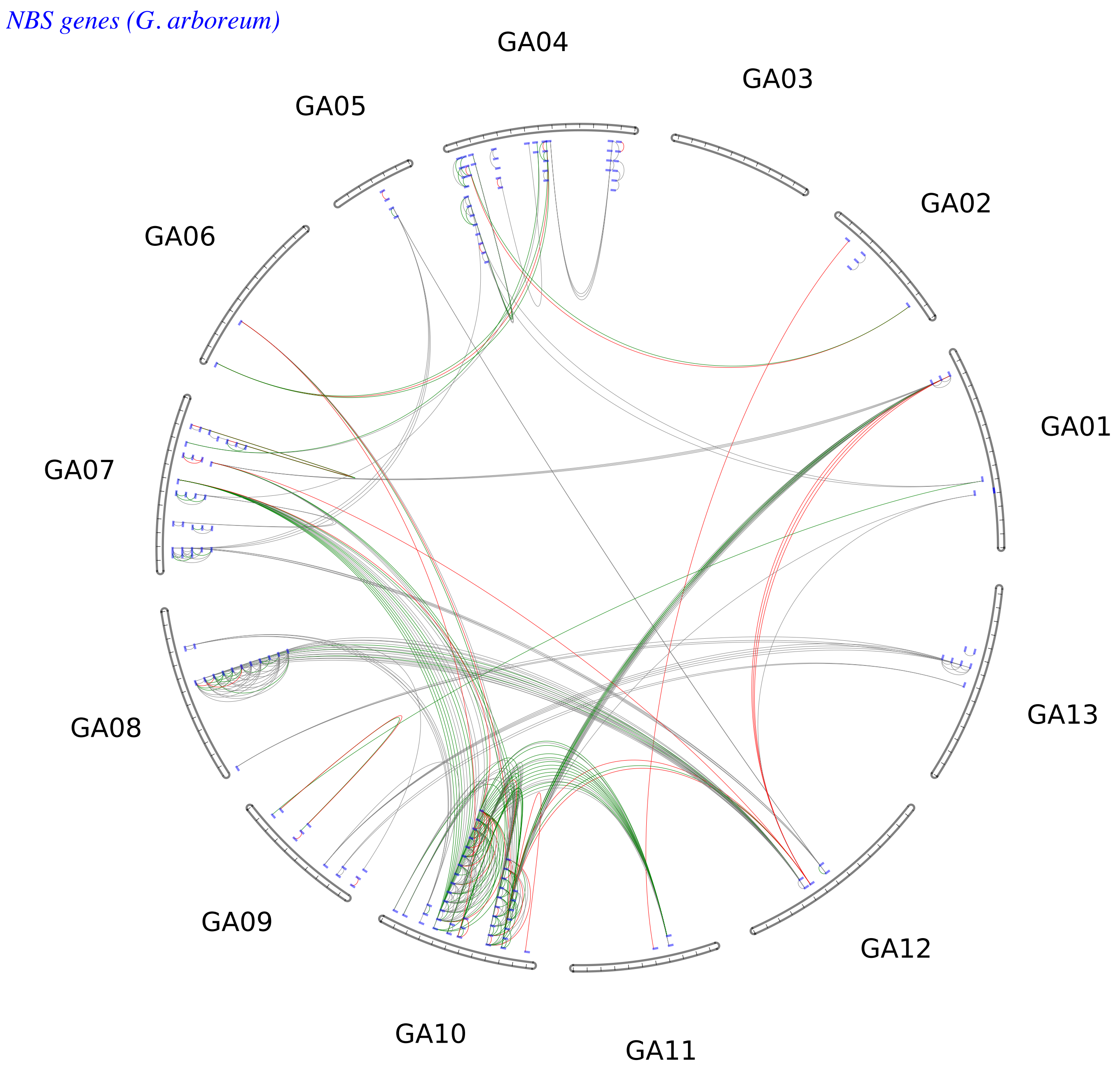
NBS genes (G. arboreum)
GA04
GA03
GA05
GA02
GA06
GA01
GA07
GA13
GA08
GA12
GA09
GA10
GA11

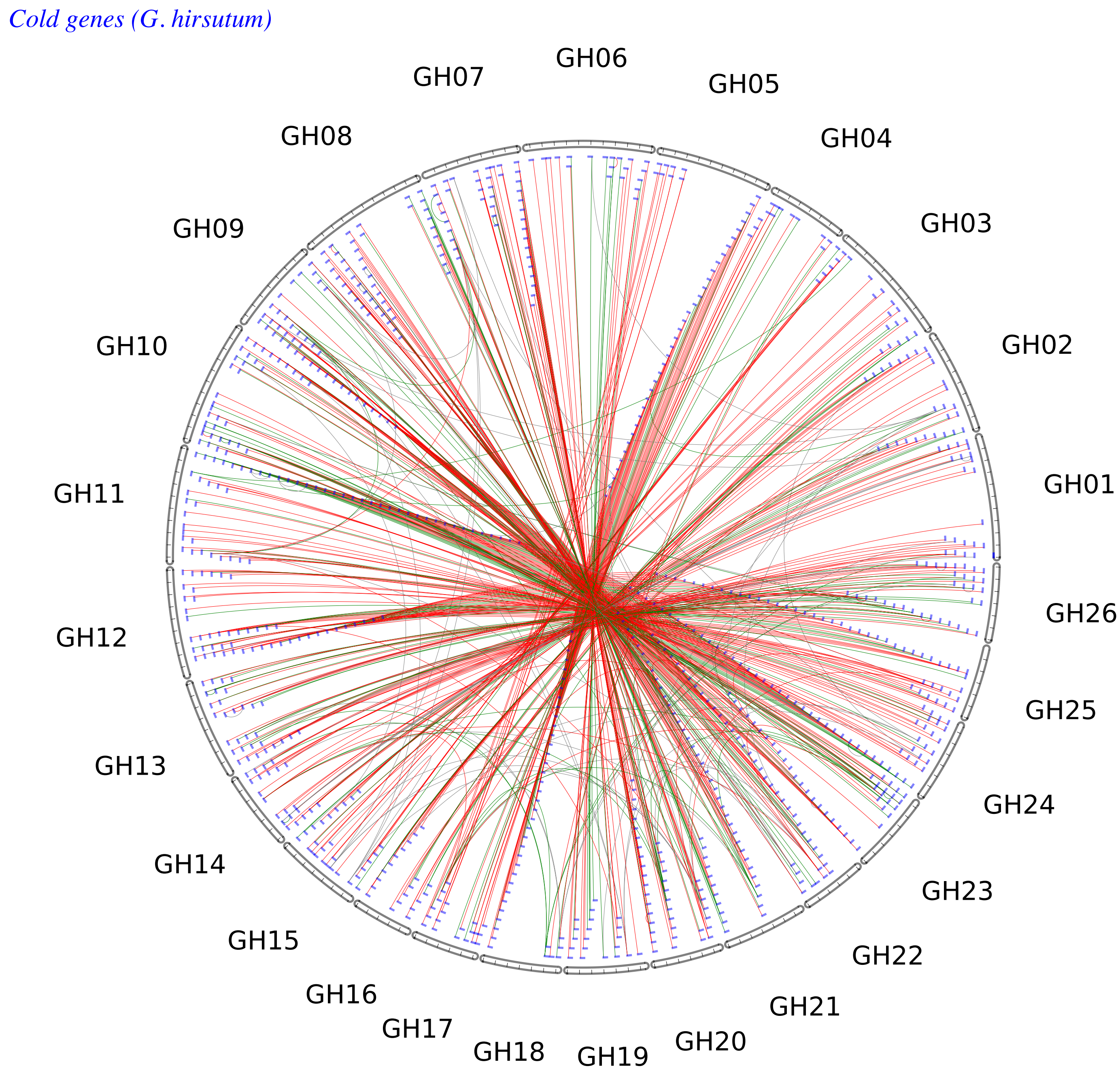

Cold genes (G. hirsutum)
GH06
GH07
GH05
GH08
GH04
GH09
GH03
GH10
GH02
GH11
GH01
GH26
GH12
GH25
GH13
GH24
GH14
GH23
GH15
GH22
GH16
GH21
GH17
GH20
GH18
GH19

NBS genes (G. hirsutum)

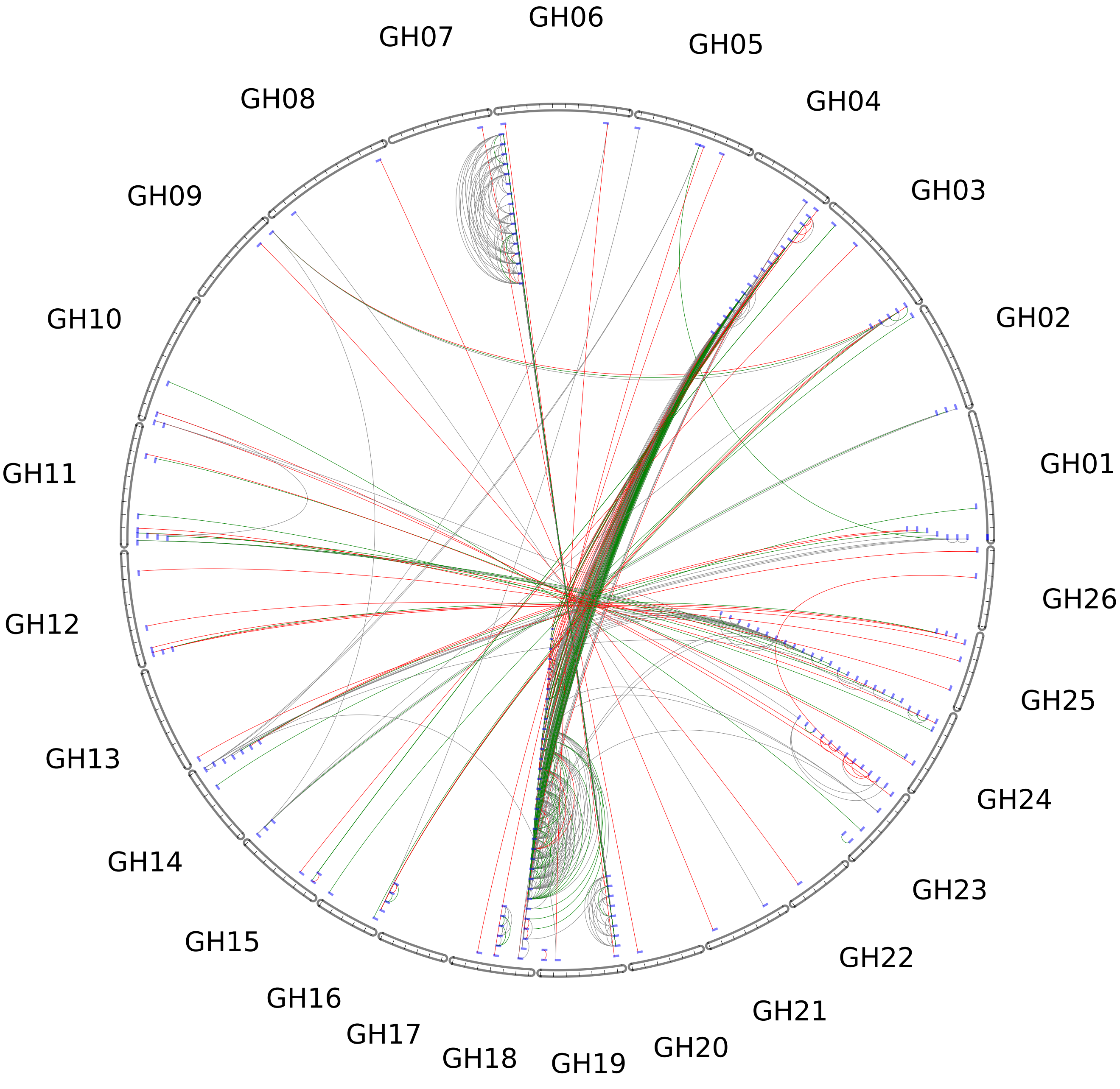

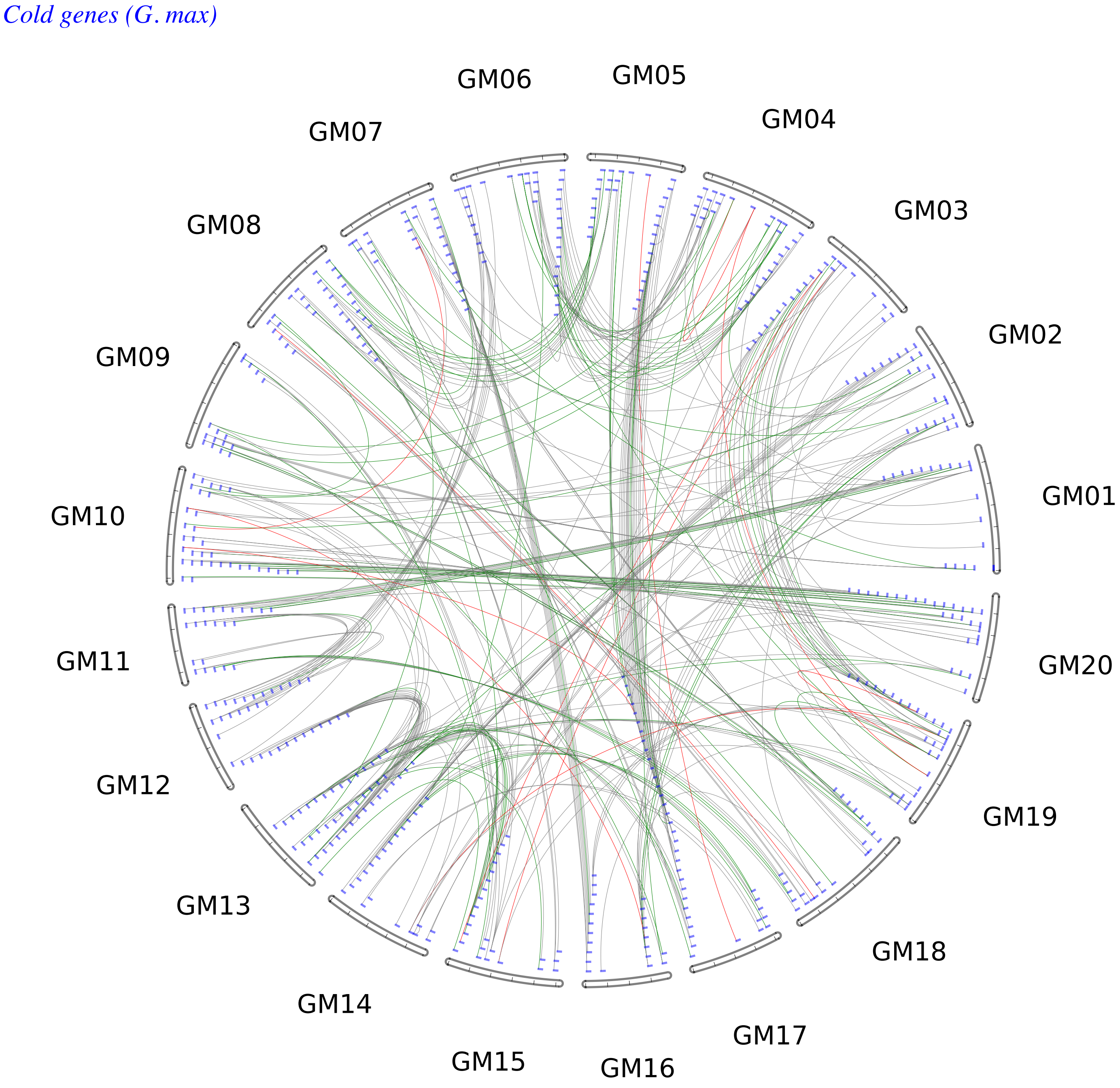

Cold genes (G. max)
GM01
GM02
GM03
GM04
GM05
GM06
GM07
GM08
GM09
GM10
GM11
GM12
GM13
GM14
GM15
GM16
GM17
GM18
GM19
GM20

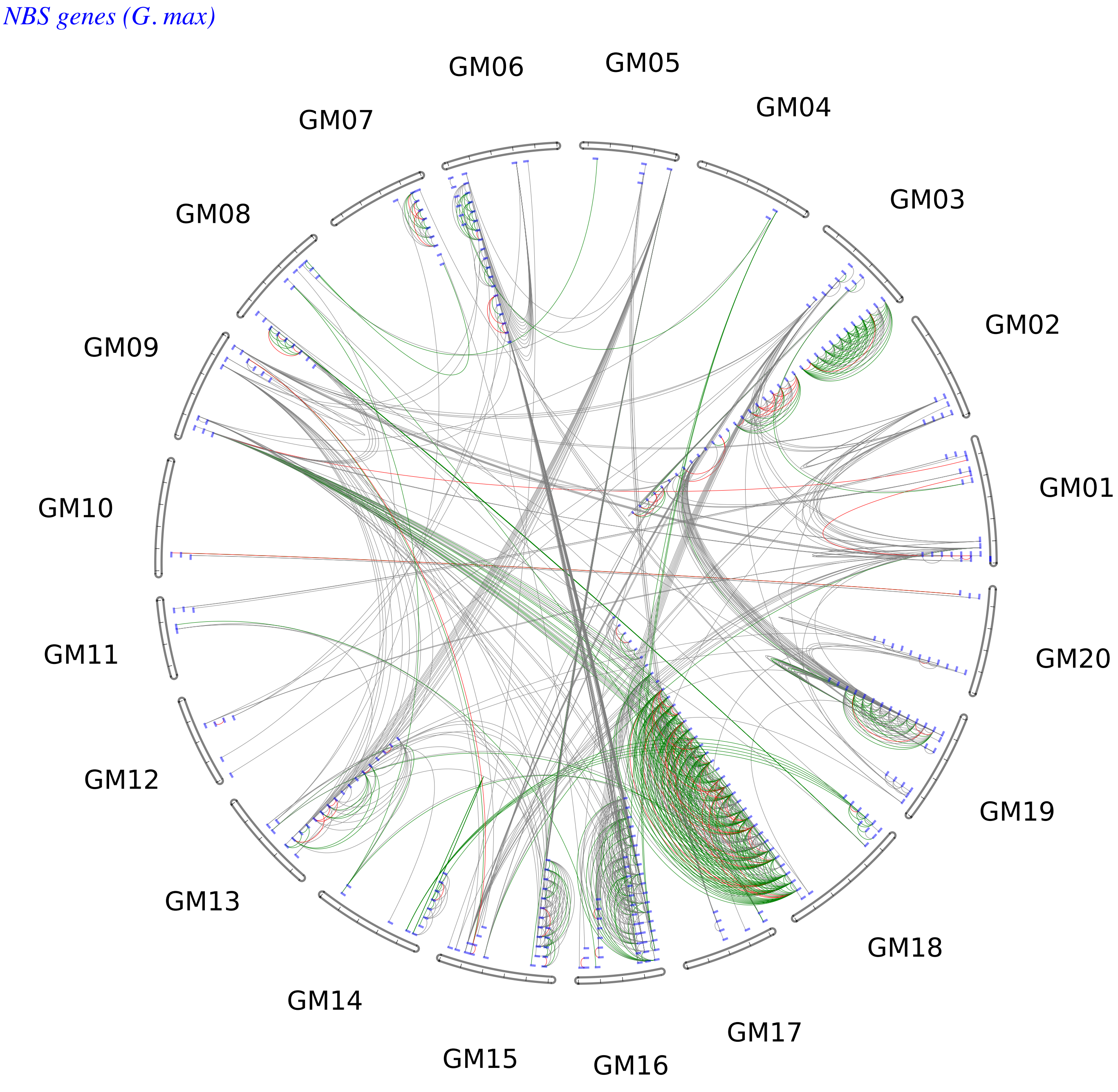
NBS genes (G. max)
GM01
GM02
GM03
GM04
GM05
GM06
GM07
GM08
GM09
GM10
GM11
GM12
GM13
GM14
GM15
GM16
GM17
GM18
GM19
GM20
